# Seco-Tetracenomycins from the Marine-Derived Actinomycete *Saccharothrix* sp. 10-10

**DOI:** 10.3390/md16100345

**Published:** 2018-09-20

**Authors:** Bin Liu, Jiao Li, Minghua Chen, Xiaomeng Hao, Fei Cao, Yi Tan, Yuhui Ping, Yiguang Wang, Chunling Xiao, Maoluo Gan

**Affiliations:** 1 Institute of Medicinal Biotechnology, Chinese Academy of Medical Sciences and Peking Union Medical College, Beijing 100050, China; bin0629bin@163.com (B.L.); jiaoli930911@126.com (J.L.); 15210165499@163.com (M.C.); xiaomhao@163.com (X.H.); tanyiyi123@126.com (Y.T.); 13552532543@163.com (Y.W.); xiaocl318@163.com (C.X.); 2 College of Pharmacy, Jiangxi University of Traditional Chinese Medicine, Nanchang 330004, China; pingyh@163.com; 3 Key Laboratory of Medicinal Chemistry and Molecular Diagnostic of Ministry of Education, College of Pharmacy, Hebei University, Baoding 071002, China; caofei542927001@163.com

**Keywords:** marine actinomycete, *Saccharothrix* sp., *seco*-tetracenomycin, saccharothrixone, structure-activity relationship

## Abstract

Six new tetracenomycin congeners, saccharothrixones E–I (**1**–**5**) and 13-de-*O*-methyltetracenomycin X (**6**), were isolated from the rare marine-derived actinomycete *Saccharothrix* sp. 10-10. Their structures were elucidated by spectroscopic analysis and time-dependent density functional theory (TDDFT)-electronic circular dichroism (ECD) calculations. Saccharothrixones G (**3**) and H (**4**) are the first examples of tetracenomycins featuring a novel ring-A-cleaved chromophore. Saccharothrixone I (**5**) was determined to be a *seco*-tetracenomycin derivative with ring-B cleavage. The new structural characteristics, highlighted by different oxidations at C-5 and cleavages in rings A and B, enrich the structural diversity of tetracenomycins and provide evidence for tetracenomycin biosynthesis. Analysis of the structure–activity relationship of these compounds confirmed the importance of the planarity of the naphthacenequinone chromophore and the methylation of the polar carboxy groups for tetracenomycin cytotoxicity.

## 1. Introduction

Aromatic polyketides constitute a large group of structurally diverse natural products biosynthesized by type II polyketide synthases (PKS II) [1]. Many of these natural products have been widely used as antibacterial, antifungal, and anticancer agents [2,3]. Based on the polyphenolic ring systems and their biosynthetic pathways, bacterial aromatic polyketides are classified as anthracyclines, angucyclines, aureolic acids, tetracyclines, tetracenomycins (Tcms), benzoisochromanequinones, and pentangular polyphenols [4]. Tcms, which have been isolated from *Streptomyces glaucescens* and *Streptomyces olivaceus*, represent a separate group of aromatic polyketides featuring a tetracyclic naphthacenequinone chromophore with highly hydroxylated cyclohexenone moiety, and exhibit moderate antibacterial and antitumor activities [5]. Terrestrial and marine actinomycetes are particularly rich sources of bioactive PKS II metabolites. With the advent of molecular tools and advances in biosynthetic-mechanism research, genetic-level investigation of bacterial aromatic polyketides has become possible [6]. PCR-based genetic screening has become a useful approach to identify novel metabolites with desired structural characteristics [7,8].

**Figure 1:**
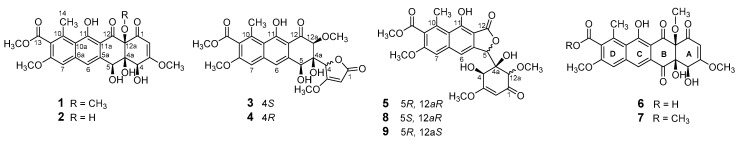
Structures of compounds **1**–**9**.

As part of our screening program for new antibiotics from marine-derived microorganisms [9,10,11], we previously identified Tcm X (**7**) and four Tcm analogs, saccharothrixones A, B (**8**), C (**9**), and D, from the rare actinomycete *Saccharothrix* sp. 10-10 by PCR screening [12,13]. To investigate the structural diversity of Tcms produced by strain 10-10 and, thereby, to explore structure–activity relationships, we further analyzed the LC-MS data of other fractions of the culture extracts and identified six new Tcm analogs, saccharothrixones E–I (**1**–**5**), and 13-de-*O*-methyltetracenomycin X (**6**, Figure 1). Saccharothrixone E (**1**) and F (**2**) were identified as 5-de-oxo-5-hydroxy derivatives of Tcm X and C, respectively. Saccharothrixones G (**3**) and H (**4**) were C-4 epimers of *seco*-tetracenomycins that featured a unique ring-A-cleaved chromophore. This paper describes the isolation and structural characterization of the six new tetracenomycin congeners **1**–**6,** as well as the structure–activity relationship of their cytotoxicity.

## 2. Results

Saccharothrixone E (**1**) was isolated as a yellow powder. Its molecular formula was determined to be C_24_H_24_O_11_ by HRESIMS, which is 2 mass units higher than Tcm X (**7**). The UV spectrum of **1** exhibited absorption maxima at 266, 277, and 382 nm. The ^1^H NMR spectrum in acetone-*d*_6_ (Table 1) displayed characteristic signals for two aromatic protons (δ_H_ 7.44 (s, H-6) and 7.20 (s, H-7)), an olefinic proton (δ_H_ 5.61 (s, H-2)), and two oxygenated methine protons (δ_H_ 4.77 (brs, H-5) and 4.62 (brs, H-4)). In addition, an olefinic methyl and four methoxy signals were observed at δ_H_ 2.79–3.94 ppm. In the ^1^H NMR spectrum recorded in DMSO-*d*_6_ (Table S1), characteristic feature was the presence of four exchangeable protons at δ_H_ 14.70 (brs), 6.07 (brs), 5.47 (d), and 5.23 (s). These spectroscopic data implied that the structure of **1** is closely related to **7**. The ^13^C NMR (Table 2) and HSQC spectra of compound **1** revealed the presence of 24 carbons, and also indicated its close similarity to **7** (Table S2). One of the three ketonic carbonyl resonances observed for **7** was missing in **1**, and, instead, an *O*-bearing methine carbon signal was observed at δ_C_ 69.6. This indicated that one of the ketone groups in **7** was replaced by a hydroxy-substituted carbon in **1**. A detailed comparison of the NMR data of **1** and **7** revealed the structural similarities in their A, C, and D rings.

**Figure 2:**
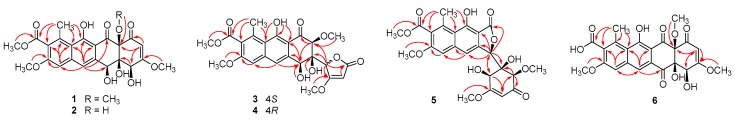
Key HMBC correlations of **1**–**6**.

The HMBC correlations (Figure 2) from H-7 to C-8 (δ_C_ 158.1), C-9, C-10 (δ_C_ 137.8), C-10a, and C-13 (δ_C_ 168.6), and from H-6 to C-5a, C-6a, C-10a, C-11 (δ_C_ 166.7), and C-11a confirmed that the naphthalene ring substitution pattern of **1** is identical to that of **7**. The correlation from H-2 to C-1 (δ_C_ 193.6) and the relatively weaker ^4^*J* correlation from H-6 to C-12 (δ_C_ 202.2) observed in the HMBC spectrum suggested that the ketone groups at C-1 and C-12 that are characteristic for tetracenomycins remained intact in **1**. The strong ^3^*J* HMBC correlation from H-6 to C-5 (δ_C_ 69.6) indicated that the carbonyl group at C-5 in **7** was replaced by an oxygenated methine group in **1**. The remaining HMBC correlations confirmed structure **1** as 5-de-oxo-5-hydroxytetracenomycin X.

**Figure 3:**
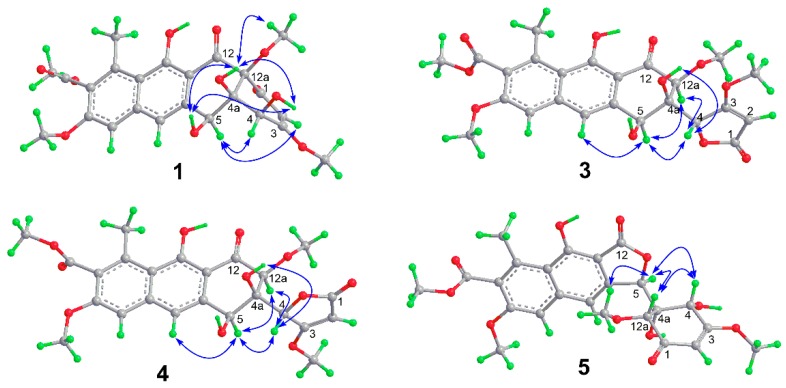
Key NOE correlations of **1** and **3**–**5**.

**Figure 4:**
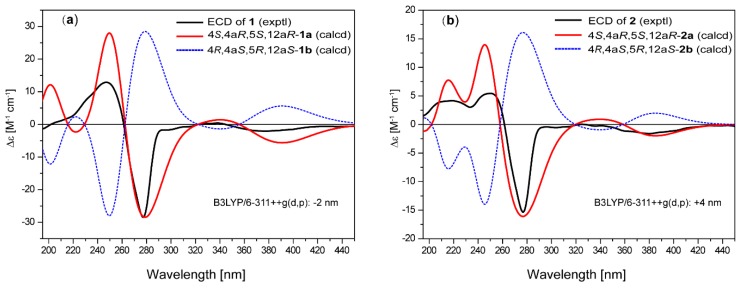
Comparison of experimental and calculated electronic circular dichroism (ECD) spectra of (**a**) **1** and (**b**) **2**.

The relative configuration of compound **1** was determined by interpretation of the ROESY data. The ROESY cross-peaks (Figure 3) of OH-4a with OH-4, OH-5, and 12a-OCH_3_, and of OH-4 with OH-5 indicated that they were all located on the same β-face of the ring. The ROESY correlations of H-5 with H-4 and H-2 indicated that H-4 and H-5 were on the α-face. Since the relative structure of ring A in **1** is consistent with those in Tcms C [5] and X (**7**) [14], it was deduced that compound **1** has the same absolute configuration as **7**. The absolute configuration of **1** was further confirmed using time-dependent density functional theory (TDDFT)-electronic circular dichroism (ECD) calculations [15]. The ECD spectra of the possible isomers of **1** obtained by geometry optimization were generated using TDDFT calculations at the B3LYP/6-311++G(d,p) level. The ECD spectrum calculated for the 4*S*,4a*R*,5*S*,12a*R*-**1a** isomer was in good agreement with the experimental ECD curve (Figure 4). Consequently, the absolute configuration of **1** was firmly assigned as 4*S*,4a*R*,5*S*,12a*R*.

The molecular formula of saccharothrixone F (**2**) was deduced to be C_23_H_22_O_11_ by HRESIMS, one CH_2_ unit less than that of **1**. The UV spectrum of **2** displayed absorption bands almost identical to those of **1**, indicating that **1** and **2** have the same chromophore. A comparison of the ^1^H and ^13^C NMR data of **1** and **2** revealed the absence of one of the four *O*-methyl groups of **1** in **2**. As compared with that of **1**, the resonance for C-12a in **2** was shifted by Δδ −5.3. These data suggested that **2** is the 12a-de-*O*-methyl analogue of **1**. This was further confirmed by 2D NMR experiments. The HMBC displayed cross-peaks from the three *O*-methyl protons at δ_H_ 3.88, 3.96, and 3.90 to C-3 (δ_C_ 176.6), C-8 (δ_C_ 158.0), and C-13 (δ_C_ 168.6), respectively, indicating that the methoxy groups were located at C-3, C-8, and C-13. The HMBC correlations of H-2 with C-12a (δ_C_ 82.5) confirmed the replacement of the 12a-*O*-methyl in **1** by a hydroxy group in **2**. The relative and absolute configurations of **2** were determined to be the same as **1** based on the similarity of the ROESY correlations and ECD spectra. This was also supported by TDDFT-ECD calculations for **2** (Figure 4).

Saccharothrixone G (**3**) was isolated as a yellow powder. The molecular formula was determined to be C_24_H_24_O_11_ by HRESIMS, which is the same as that of **1**. Its ^1^H and ^13^C NMR data (Table 1 and Table 2) differed significantly from those of **1**, suggesting a substantial structural change. Analysis of the ^1^H NMR data measured in DMSO-*d*_6_ (Table S1) indicated that one of the four exchangeable proton signals in **1** disappeared in **3**. Comparison of their ^13^C NMR/HSQC spectra revealed that the nonprotonated carbon (C-12a) in **1** was replaced by an *O*-bearing methine group in **3**, suggesting that the fused A and B rings were cleaved at C-12a. HMBC correlations of OH-4a with C-4a and C-12a, and OH-5 with C-5, indicated that the free hydroxy groups at C-4a and C-5 remained in **3**, whereas the free hydroxy group at C-4 was missing. Key HMBC correlations from H-4 to the carbonyl carbon C-1 (δ_C_ 172.2) suggested that the oxygenated C-4 was connected to C-1 via an oxygen atom to form a lactone ring. Finally, HMBC correlations from H-4 to C-4a, C-5, and C-12a indicated the connectivity of C-4 and C-4a, thus completing the establishment of the planar structure of **3**. 

**Figure 5:**
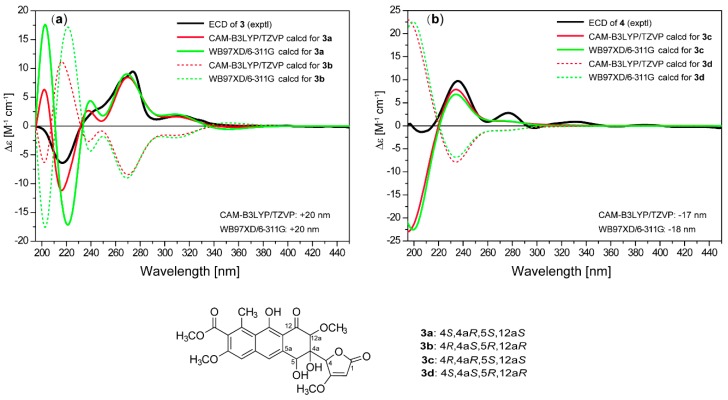
Comparison of experimental curve of (**a**) **3** and (**b**) **4**, and calculated ECD spectra for **3a**–**3d**.

The relative configuration of **3** was established by analysis of the NOE correlations. The NOE enhancement between H-12a and H-5 revealed their 1,3-diaxial positions and a half-chair conformation for ring B (Figure 3). The NOE correlations of H-4 with H-5 and H-12a indicated that the methine group C-4 was in an equatorial orientation in ring B while OH-4a was axial. The ECD spectra of four possible isomers were then calculated using the TDDFT-ECD method. The calculated curves for 4*S*,4a*R*,5*S*,12a*S*-**3a** (Figure 5) at both CAM-B3LYP/TZVP and WB97XD/6-311++G(d,p) levels were in good agreement with the experimental spectrum, thus determining the absolute configuration of **3** to be 4*S*,4a*R*,5*S*,12a*S.*

Saccharothrixone H (**4**) was determined to be a diastereoisomer of **3** based on their identical molecular formula (C_24_H_24_O_11_) and their similar NMR data. This was verified by HMBC correlations (Figure 2). In addition, similar correlations (Figure 3) observed in the ROESY spectra of **4** revealed that the relative configuration of ring B in **4** was also identical to that of **3**. This was suggestive of a 4-epimer of **3**. The calculated ECD spectrum of 4*R*,4a*R*,5*S*,12a*S*-**3c** fit well with the experimental spectrum of **4**. Therefore, the structure of **4** was assigned and named saccharothrixone H.

**Figure 6:**
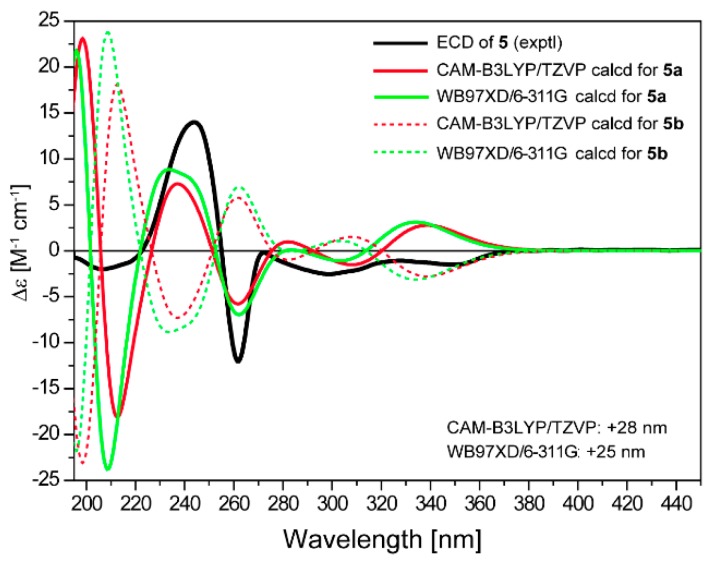
Comparison of experimental curve of **5** and calculated ECD spectra for 4*S*,4a*R*,5*R*,12a*R-***5a** and 4*R*,4a*S*,5*S*,12a*S-***5b**.

Saccharothrixone I (**5**) was obtained as a white powder. The HRESIMS data established its molecular composition as C_24_H_24_O_11_, identical with the molecular formulae of **3**, **4**, and saccharothrixones B (**8**) and C (**9**) [12]. The UV, ^1^H NMR, and ^13^C NMR spectra were very similar to those of **8** (Table S3) except for the chemical shifts of the protons and carbon signals in rings A and B, indicating that **5** was a diastereoisomer of **8**. The 2D NMR data of **5** (Figure 2) confirmed it had the same planar structure as **8**. The ROESY correlation between H-4 and H-12a indicated a 1,3-diaxial interaction. ROESY correlations of H-5 with H-4 and H-12a illustrated an equatorial orientation of the methine group C-5 in ring A, and thus an axial position for OH-4a. Therefore, the relative configuration of ring A in **5** was determined to be identical to that of **8**, suggesting that **5** is a 5-epimer of **8**. The ECD spectrum of **5** exhibited negative Cotton effects (CEs) at 262 nm and between 299 and 350 nm, and a positive CE at 244 nm. The observed negative CE around 262 nm ascribed for the n→π* transition of the α,β-unsaturated γ-lactone [16], was opposite to that of **8** (Figure S1), indicating that the configuration of C-5 in the lactone ring B of **5** was opposite to that of **8**. This was further confirmed by the TDDFT-ECD calculation, which showed good agreement of the calculated spectrum for 4*S*,4a*R*,5*R*,12a*R*-**5a** (Figure 6) with the experimental curve. Therefore, the absolute configuration of **5** was assigned as 4*S*,4a*R*,5*R*,12a*R.*

Compound **6** has the molecular formula C_23_H_20_O_11_, one CH_2_ unit less than Tcm X (**7**), as determined by HRESIMS and NMR data. The NMR data of **6** were similar to those of **7** except for the absence of one methoxy group signal in **6**. The HMBC correlations from the *O*-methyl protons at δ_H_ 3.80, 4.01, and 3.56 to C-3 (δ_C_ 174.8), C-8 (δ_C_ 159.3), and C-12a (δ_C_ 89.0), respectively, located the methoxy groups at C-3, C-8 and C-12a, suggesting that the methoxy group at C-13 in **7** was absent in **6**. Therefore, the structure of **6** was determined to be 13-de-*O*-methyltetracenomycin X. 

The identification of compounds **1** and **2** supports our previously proposed biosynthetic pathway for the ring-B cleaved tetracenomycin derivatives saccharothrixones A–C [12]. In that biosynthetic pathway, saccharothrixones A–C are derived from the intermediate **2** and its epimer of C-5, which undergo an intramolecular nucleophilic addition from OH-5 to 12-oxo group and simultaneous cleavage of ring B. Similarly, the nucleophilic addition from OH-4 to 1-oxo group of **1** would result in a divergent pathway committed to the formation of saccharothrixones G (**3**) and H (**4**) (Scheme S1) [12]. The coisolation of the different C-4, C-5, and C-12a epimers of *seco*-tetracenomycins indicated that the cleavage of rings A and B by the intramolecular nucleophilic addition was not stereocontrolled. 

In a previous study [12], we reported that Tcm X and its isomer, saccharothrixone D, showed moderate cytotoxicity (5.4–20.8 µM) against the HepG2, MCF-7, and K562 human cancer cell lines, whereas the B-ring-cleaved derivatives saccharothrixones A–C were inactive at a concentration of 100 μM. To further evaluate the structure–activity relationship of these tetracenomycin congeners, we examined the cytotoxicity of compounds **1**–**6** against the cancer cell lines mentioned above. All the compounds were found to be inactive at 100 µM. The action mechanism of tetracenomycins was assumed to be intercalation with DNA, requiring flat structural moiety to move between the base pairs [17]. These results confirmed that the naphthacenequinone chromophore and the planarity of the molecules are vital for their cytotoxicity. The cleaved naphthacenequinone chromophore with a large substituent (ring A) in saccharothrixones A–C and G–I (**3**–**5**) probably blocks the intercalation with DNA, causing loss of cytotoxicity. The replacement of the ketone group by the hydroxy group at C-5 in compounds **1** and **2** changes the planarity of ring B, which could lower the effectiveness of intercalation. Compound **6**, which differs from Tcm X only at the C-13 substituent (carboxy vs. methoxycarbonyl), showed no effect at 100 µM, indicating that the free carboxy group caused a significantly negative interaction with the DNA. Rohr et al. previously reported that tetracenomycin derivatives with a free hydroxy group at C-8 (elloramycinone) or C-12a (Tcm C) were less active than Tcm X, which has methoxy groups at C-8 and C-12a [17]. Our results further confirmed the importance of the methylation of the polar hydroxy and carboxy groups for their cytotoxic activity. In additions, **1**–**6** were also evaluated for antibacterial activity, but were found to be inactive (MIC > 64 µg/mL).

## 3. Materials and Methods

### 3.1. General Experimental Procedures

Optical rotations were measured on a Perkin-Elmer model 343 polarimeter (Perkin-Elmer Inc., Waltham, MA, USA). UV and CD spectra were acquired on a JASCO-815 CD spectrometer (Jasco Inc., Tokyo, Japan). IR spectra were measured on a Nicolet 5700 FT-IR microscope spectrometer (Thermo Electron Corp., Madison, WI, USA) (FT-IR microscope transmission). 1D- and 2D-NMR spectra were acquired at 500 or 600 MHz for ^1^H and 125 or 150 MHz for ^13^C, respectively, on a Bruker AVANCE III HD 600 MHz spectrometer (Bruker Corp., Karlsruhe, Germany) in acetone-*d*_6_, CD_3_OD, and DMSO-*d*_6_ using tetramethylsilane as an internal reference. ESIMS data were recorded with an Agilent 1100 LC/MSD (Agilent Technologies, Ltd., Santa Clara, CA, USA) with a G1956B single quadrupole mass spectrometer. HRESIMS data were obtained using a Thermo LTQ Orbitrap XL mass spectrometer (Thermo Fisher Scientific, Waltham, MA, USA). Flash chromatography was performed on an Ez Purifier (Suzhou Lisure Science Co., Ltd., Suzhou, China). TLC analysis was carried out using glass precoated silica-gel GF254 plates (Qingdao Marine Chemical Inc., Qingdao, China). Spots were visualized under UV light and by spraying with 7% H_2_SO_4_ in 95% aqueous EtOH followed by heating. Column chromatography was carried out using silica gel (200–300 mesh, Qingdao Marine Chemical Inc., Qingdao, China) and Sephadex LH-20 (GE Healthcare Biosciences Co., Uppsala, Sweden). HPLC analysis was conducted on a Shimadzu HPLC system (Kyoto, Japan) equipped with an LC-20AD pump and an SPD-M20A diode array detector. Preparative HPLC separation was performed with a Shimadzu LC-20AP binary pump (Kyoto, Japan) equipped with an SPD-M20A diode array detector. 

### 3.2. Fermentation and Isolation

Strain *Saccharothrix* sp. 10-10 was isolated from a marine-sediment sample and fermentation was performed as described previously [12]. The fermentation broth (30 L) was loaded onto the Diaion HP-20 and eluted with 50% and 80% acetone. The combined aqueous acetone-eluting fractions were concentrated and extracted with EtOAc to afford a residue. The residue was subjected to silica gel column chromatography (CC) eluting with a step-gradient of CH_2_Cl_2_–MeOH (100:0–0:100, *v*/*v*) to give 12 fractions (F_1_–F_12_). Fraction F_5_ was separated on Sephadex LH-20 CC eluting with 95% MeOH to afford 11 subfractions (F_5-1_–F_5-11_). Subfraction F_5-4_ was purified on a semipreparative C_18_ RP-HPLC column (Cosmosil 5μm, 10 mm × 250 mm, 2 mL/min, 55% MeOH) to yield **3** (2.6 mg), **4** (1.5 mg), and **5** (18 mg). Subfraction F_5-7_ was separated by RP C18 flash chromatography with a gradient system of 5%–70% MeOH and then purified by RP-HPLC (Cosmosil C18-MSII 5μm, 10 mm × 250 mm, 2 mL/min, 52% MeOH) to give **1** (22 mg). Fraction F_6_ was subjected to CC on Sephadex LH-20 CC (CH_2_Cl_2_–MeOH, 1:1) to give 8 subfractions (F_6-1_–F_6-8_). Subfraction F_6-8_ was separated by C18 flash chromatography (30–60% MeOH) and followed by RP C_18_ HPLC (Cosmosil C18-MSII 5 µm, 10 mm × 250 mm, 34%MeCN containing 0.1%TFA, 2 mL/min) to afford **2** (8.5 mg). Fraction F_11_ was subjected to RP-HPLC (Capcell AQ C18 5μm, 20 mm × 250 mm, 40% MeOH containing 0.1% formic acid, 8 mL/min) to yield **6** (14 mg).

Saccharothrixone E (**1**): Yellow amorphous powder; [α]${}_{D}^{\mathrm{20}}$ −121.5 (*c* 0.31, MeOH); UV (MeOH) *λ*_max_ (log*ε* 239 (4.30), 266 (4.50), 277 (4.54), 317 (3.80), 328 (3.75), 382 (3.82) nm; ECD (5.6 × 10^−4^ M, MeOH) *λ*_max_ (Δ*ε*) 247 (+12.89), 277 (−28.45), 334 (+0.31), 377 (−2.08) nm; IR *v*_max_ (cm^−1^): 3469, 2948, 1730, 1659, 1606, 1374, 1221, 1089, 946, 839; ^1^H NMR (acetone-*d*_6_, 600 MHz) data, Table 1; ^13^C NMR (acetone-*d*_6_, 150 MHz) data, Table 2; HRESI-MS *m/z* 489.1391 [M + H]^+^ (calcd for C_24_H_25_O_11_ 489.1391).

Saccharothrixone F (**2**): Yellow amorphous powder; [α]${}_{D}^{\mathrm{20}}$ −120.6 (*c* 0.15, MeOH); UV (MeOH) *λ*_max_ (log*ε*) 232 (4.06), 268 (4.28), 277 (4.38), 318 (3.60), 330 (3.53), 382 (3.67) nm; ECD (4.2 × 10^−4^ M, MeOH) *λ*_max_ (Δ*ε*) 218 (+4.16), 250 (+5.45), 276 (−15.33), 381 (−1.60) nm; IR *v*_max_ (cm^−1^): 3199, 2921, 1680, 1604, 1205, 1140, 840, 801, 723; ^1^H NMR (acetone-*d*_6_, 600 MHz) data, Table 1; ^13^C NMR (acetone-*d*_6_, 150 MHz) data, Table 2; HRESI-MS *m/z* 475.1236 [M + H]^+^ (calcd for C_23_H_23_O_11_ 475.1255); 497.1053 [M + Na]^+^ (calcd for C_23_H_2__2_O_11_Na 497.1054).

Saccharothrixone G (**3**): Yellow amorphous powder; [α]${}_{D}^{\mathrm{20}}$ + 52.3 (*c* 0.17, MeOH); UV (MeOH) *λ*_max_ (log*ε*) 227 (4.28), 275 (4.51), 315 (3.65), 328 (3.56), 377 (3.75) nm; ECD (5.3 × 10^−4^ M, MeOH) *λ*_max_ (Δ*ε*) 217 (−6.40), 274 (+9.45), 315 (+1.74) nm; IR *v*_max_ (cm^−1^): 3367, 2947, 1735, 1622, 1368, 1221, 1087, 1053, 993, 948; ^1^H NMR (acetone-*d*_6_, 600 MHz) data, Table 1; ^13^C NMR (acetone-*d*_6_, 150 MHz) data, Table 2; HRESI-MS *m/z* 487.1229 [M − H]^−^ (calcd for C_24_H_23_O_11_ 487.1235).

Saccharothrixone H (**4**): Yellow amorphous powder; [α]${}_{D}^{\mathrm{20}}$ + 48.3 (*c* 0.05, MeOH); UV (MeOH) *λ*_max_ (log*ε*) 226 (4.26), 278 (4.38), 316 (3.68), 328 (3.63), 382 (3.71) nm; ECD (6.8 × 10^−4^ M, MeOH) *λ*_max_ (Δ*ε*) 206 (−1.35), 236 (+9.71), 277 (+2.79), 331 (+0.87) nm; IR *v*_max_ (cm^−1^): 3383, 2927, 1736, 1621, 1375, 1218, 1092, 989, 802; ^1^H NMR (acetone-*d*_6_, 600 MHz) data, Table 1; ^13^C NMR (acetone-*d*_6_, 150 MHz) data, Table 2; HRESI-MS *m/z* 487.1226 [M − H]^−^ (calcd for C_24_H_23_O_11_ 487.1235).

Saccharothrixone I (**5**): White amorphous powder; [α]${}_{D}^{\mathrm{20}}$ + 12.7 (*c* 0.98, MeOH); UV (MeOH) *λ*_max_ (log*ε*) 258 (4.60), 263 (4.68), 352 (3.71) nm; ECD (5.2 × 10^−4^ M, MeOH) *λ*_max_ (Δ*ε*) 206 (−2.01), 244 (+14.00), 262 (−12.01), 299 (−2.54), 350 (−1.51) nm; IR *v*_max_ (cm^−1^): 3346, 2952, 1730, 1677, 1615, 1366, 1203, 1105, 994, 723; ^1^H NMR (acetone-*d*_6_, 600 MHz) data, Table 1; ^13^C NMR (acetone-*d*_6_, 150 MHz) data, Table 2; HRESI-MS *m/z* 487.1226 [M − H]^−^ (calcd for C_24_H_23_O_11_ 487.1235).

13-De-*O*-methyltetracenomycin X (**6**): Yellow amorphous powder; [α]${}_{D}^{\mathrm{20}}$ + 38.8 (*c* 1.13, MeOH); UV (MeOH) *λ*_max_ (log*ε*) 216 (4.47), 240 (4.51), 291 (4.76), 397 (4.19), 414 (4.23) nm; ECD (5.3 × 10^−4^ M, MeOH) *λ*_max_ (Δ*ε*) 201 (+13.60), 227 (−4.05), 262 (+28.51), 350 (−5.55), 420 (−1.09) nm; IR *v*_max_ (cm^−1^): 3357, 2934, 1679, 1602, 1369, 1236, 1122, 838, 601; ^1^H NMR (CD_3_OD, 600 MHz), Table 1; ^13^C NMR (CD_3_OD, 150 MHz) data, Table 2; HRESI-MS *m/z* 473.1101 [M + H]^+^ (calcd for C_23_H_21_O_11_ 473.1078),495.0923 [M + Na]^+^ (calcd for C_23_H_20_O_11_Na 495.0898).

### 3.3. ECD Calculations

Conformational analysis was carried out via Monte Carlo searching in the MMFF94 molecular mechanics force field on Molecular Operating Environment (MOE) software [18]. The lowest energy conformers within 10 kcal/mol were subjected to further DFT calculations. The geometry of the conformers were optimized at the B3LYP/6-31+G(d,p) level with the polarizable continuum model (PCM) in MeOH using the Gaussian 09 program [19]. The B3LYP/6-31+G(d,p)-optimized conformers within 4 kcal/mol were then reoptimized at the B3LYP/6-311+G(d,p) level in MeOH. The harmonic vibrational frequencies were calculated at the same level to confirm their stability and to provide their relative thermal free energy (ΔG), which are used to assess their equilibrium populations. TDDFT-ECD calculation of the low-energy conformers (>1%) were performed using the TDDFT methodology at the B3LYP/6-311++G(d,p) level for **1** and **2** and the CAM-B3LYP/TZVP and WB97XD/6-311++G(d,p) levels for **3**–**5** in MeOH with the PCM model. ECD spectrum of each conformer was simulated by the SpecDis program [20] using a Gaussian function band width σ = 0.30 eV. Final ECD spectra for 4*S*,4s*R*,5*S*,12a*R*-**1a**, 4*S*,4a*R*,5*S*,12a*R*-**2a**, 4*S*,4a*R*,5*S*,12a*S*-**3a**, 4*R*,4a*R*,5*S*,12a*S*-**3c**, and 4*S*,4a*R*,5*R*,12a*R-***5a** were generated by averaging the calculated data of the lowest energy conformers for each structure according to Boltzmann distribution theory at 298 K based on Gibbs free energies. The corresponding theoretical ECD spectra of 4*R*,4a*S*,5*R*,12a*S*-**1b**, 4*R*,4a*S*,5*R*,12a*S*-**2b**, 4*R*,4a*S*,5*R*,12a*R*-**3b**, 4*S*,4a*S*,5*R*,12a*R*-**3d**, and 4*R*,4a*S*,5*S*,12a*S-***5b** were depicted by inverting those of **1a**, **2a**, **3a**, **3c**, and **5a**, respectively.

### 3.4. Biological Assays

The cytotoxicities of the tested compounds against the human cancer cells HepG2 (hepatocellular carcinoma), MCF-7 (breast adenocarcinoma), and K562 (leukemia) were evaluated by the sulforhodamine B (SRB) assay as described previously [12]. The antibacterial assay was performed by using the agar dilution method [12].

## 4. Conclusions

Six new tetracenomycin derivatives including three *seco*-tetracenomycins were isolated from the rare marine-derived actinomycete *Saccharothrix* sp. 10-10. Saccharothrixones G (**3**) and H (**4**) are the first examples of tetracenomycins featuring a novel ring-A-cleaved chromophore. Saccharothrixone I (**5**), together with previously identified saccharothrixones A–C from the same cultures, are the only *seco*-tetracenomycins with a cleaved ring-B skeleton isolated from microbial natural products. This finding not only adds diversity of tetracenomycins, but also provides evidence for tetracenomycin-related polyketides biosynthesis. The structure-activity relationship study indicated that the planarity of the chromophore and methylation of the polar carboxy groups are important for tetracenomycin cytotoxicity.

## Figures and Tables

**Table 1:** ^1^H NMR spectroscopic data for compounds **1**–**6** (δ_H_, mult. (*J* in Hz)) ^a^.

No.	1 (acetone-*d_6_*)	2 (acetone-*d_6_*)	3 (acetone-*d_6_*)	4 (acetone-*d_6_*)	5 (acetone-*d_6_*)	6 (CD_3_OD)
2	5.61, s	5.51, s	5.29, d (1.2)	5.30, d (1.2)	5.32, d (1.0)	5.53, d (1.8)
4	4.62, brs	4.93, s	5.27, s	5.44, d (1.2)	4.65, d (1.0)	4.85, brs
5	4.77, brs	5.32, s	5.01, d (1.2)	5.41, d (1.8)	5.98, s	
6	7.44, s	7.46, s	7.49, d (1.2)	7.51, d (18)	7.61, s	8.01, s
7	7.20, s	7.24, s	7.20, s	7.24, s	7.27, s	7.46, s
12a			4.33, s	3.70, s	3.93, s	
14	2.79, s	2.79, s	2.79, s	2.78, s	2.81, s	2.88, s
3-OCH_3_	3.91, s	3.88, s	3.97, s	3.96, s	3.72, s	3.80, s
8-OCH_3_	3.94, s	3.96, s	3.95, s	3.96, s	3.93, s	4.01, s
12a-OCH_3_	3.73, s		3.74, s	3.34, s	3.51, s	3.56, s
13-OCH_3_	3.90, s	3.90, s	3.90, s	3.89, s	3.90, s	

^a^ The ^1^H NMR data were measured at 500 MHz for **1** and **5,** and 600 MHz for **2**–**4** and **6**.

**Table 2:** ^13^C NMR spectroscopic data for Compounds **1**–**6** (δ_C_, mult) ^a^.

No.	1 (acetone-*d_6_*)	2 (acetone-*d_6_*)	3 (acetone-*d_6_*)	4 (acetone-*d_6_*)	5 (acetone-*d_6_*)	6 (CD_3_OD)
1	193.6, C	192.8, C	172.2, C	172.3, C	194.9, C	193.9, C
2	101.5, CH	100.4, CH	89.5, CH	89.1, CH	100.3, CH	101.9, CH
3	177.0, C	176.6, C	183.9, C	182.0, C	173.1, C	174.8, C
4	70.1, CH	74.2, CH	77.8, CH	80.3, CH	68.3, CH	70.9, CH
4a	78.6, C	79.3, C	82.0, C	78.8, C	80.3, C	86.1, C
5	69.6, CH	70.5, CH	68.4, CH	68.0, CH	81.0, CH	194.4, C
5a	142.2, C	139.9, C	140.8, C	140.8, C	143.0, C	141.9, C
6	118.2, CH	118.8, CH	118.0, CH	117.7, CH	115.0, CH	121.8, CH
6a	139.8, C	142.3, C	142.1, C	142.4, C	143.3, C	128.7, C
7	105.9, CH	105.9, CH	105.7, CH	105.9, CH	106.2, CH	108.7, CH
8	158.1, C	158.0, C	157.7, C	158.0, C	156.9, C	159.3, C
9	127.8, C	127.9, C	127.7, C	127.6, C	127.7, C	132.8, C
10	137.8, C	137.5, C	137.4, C	137.5, C	136.1, C	138.0, C
10a	118.2, C	117.9, C	117.9, C	118.0, C	118.0, C	121.9, C
11	166.7, C	165.5, C	165.2, C	167.1, C	158.5, C	167.5, C
11a	110.1, C	109.6, C	109.4, C	109.0, C	106.2, C	110.5, C
12	202.2, C	200.2, C	203.0, C	199.1, C	173.4, C	197.6, C
12a	87.8, C	82.5, C	84.2, CH	82.8, CH	84.1, CH	89.0, C
13	168.6, C	168.6, C	168.7, C	168.7, C	168.6, C	171.8, C
14	21.0, CH_3_	21.0, CH_3_	21.0, CH_3_	21.0, CH_3_	20.4, CH_3_	21.1, CH_3_
3-OMe	57.4, CH_3_	57.4, CH_3_	60.2, CH_3_	60.2, CH_3_	56.8, CH_3_	57.5, CH_3_
8-OMe	56.4, CH_3_	56.4, CH_3_	56.4, CH_3_	56.4, CH_3_	56.3, CH_3_	56.7, CH_3_
12a-OMe	56.6, CH_3_		61.5, CH_3_	58.8, CH_3_	60.6, CH_3_	56.7, CH_3_
13-OMe	52.5, CH_3_	52.5, CH_3_	52.5, CH_3_	52.5, CH_3_	52.5, CH_3_	

^a^ The ^13^C NMR data were measured at 125 MHz for **1** and **5,** and 150 MHz for **2**–**4** and **6**.

## Supplementary Materials

UV, ECD, IR, MS, 1D, and 2D NMR spectra and ECD calculation details of **1**−**6** are available online at http://www.mdpi.com/1660-3397/16/10/345/s1.

## References

[1] Das A., Khosla C. (2009). Biosynthesis of aromatic polyketides in bacteria. Acc. Chem. Res..

[2] O’Hagan D. (1991). The Polyketide Metabolites.

[3] Cragg G.M., Kingston D.G.I., Newman D.J. (2005). Anticancer Agents from Natural Products.

[4] Hertweck C. (2009). The biosynthetic logic of polyketide diversity. Angew. Chem. Int. Ed..

[5] Egert E., Noltemeyer M., Siebers J., Rohr J., Zeeck A. (1992). The structure of tetracenomycin C. J. Antibiot..

[6] Hertweck C., Luzhetskyy A., Rebets Y., Bechthold A. (2007). Type II polyketide synthases: Gaining a deeper insight into enzymatic teamwork. Nat. Prod. Rep..

[7] Gontang E.A., Gaudêncio S.P., Fenical W., Jensen P.R. (2010). Sequence-based analysis of secondary-metabolite biosynthesis in marine actinobacteria. Appl. Environ. Microbiol..

[8] Zhang W., Liu Z., Li S., Yang T., Zhang Q., Ma L., Tian X., Zhang H., Huang C., Zhang S. (2012). Spiroindimicins A–D: New bisindole alkaloids from a deep-sea-derived actinomycete. Org. Lett..

[9] Hu Y., Wang M., Wu C., Tan Y., Li J., Hao X., Duan Y., Guan Y., Shang X., Wang Y. (2018). Identification and proposed relative and absolute configurations of niphimycins C–E from the marine-derived *Streptomyces* sp. IMB7-145 by genomic analysis. J. Nat. Prod..

[10] Wang Q., Zhang Y., Wang M., Tan Y., Hu X., He H., Xiao C., You X., Wang Y., Gan M. (2017). Neo-actinomycins A and B, natural actinomycins bearing the 5H-oxazolo[4,5-b]phenoxazine chromophore, from the marine-derived streptomyces sp. IMB094. Sci. Rep..

[11] Wu C., Tan Y., Gan M., Wang Y., Guan Y., Hu X., Zhou H., Shang X., You X., Yang Z. (2013). Identification of elaiophylin derivatives from the marine-derived actinomycete *Streptomyces* sp. 7-145 using PCR-based screening. J. Nat. Prod..

[12] Gan M., Liu B., Tan Y., Wang Q., Zhou H., He H., Ping Y., Yang Z., Wang Y., Xiao C. (2015). Saccharothrixones A–D, tetracenomycin-type polyketides from the marine-derived actinomycete *Saccharothrix* sp. 10-10. J. Nat. Prod..

[13] Liu B., Tan Y., Gan M., Zhou H., Wang Y., Ping Y., Li B., Yang Z., Xiao C. (2014). Identification of tetracenomycin X from a marine-derived *Saccharothrix* sp. guided by genes sequence analysis. Acta Pharm. Sin..

[14] Anderson M.G., Khoo C.L., Rickards R.W. (1989). Oxidation processes in the biosynthesis of the tetracenomycin and elloramycin antibiotics. J. Antibiot..

[15] Bringmann G., Bruhn T., Maksimenka K., Hemberger Y. (2009). The assignment of absolute stereostructures through quantum chemical circular dichroism calculations. Eur. J. Org. Chem..

[16] Legrand M., Rougier M.J., Kagan H.B. (1977). Stereochemistry: Fundamentals and Methods.

[17] Rohr J., Zeeck A. (1990). Structure-activity relationships of elloramycin and tetracenomycin C. J. Antibiot..

[18] (2009). Molecular Operating Environment (MOE).

[19] Frisch M.J., Trucks G.W., Schlegel H.B., Scuseria G.E., Robb M.A., Cheeseman J.R., Scalmani G., Barone V., Mennucci B., Petersson G.A. (2009). Gaussian 09, Revision a.1.

[20] Bruhn T., Schaumlöffel A., Hemberger Y., Bringmann G. (2013). Specdis: Quantifying the comparison of calculated and experimental electronic circular dichroism spectra. Chirality.

