# Supplementary material for: Seco-Tetracenomycins from the Marine-Derived Actinomycete Saccharothrix sp. 10-10
Source: Mar Drugs. 2018 Sep 20;16(10):345. doi: 10.3390/md16100345 (PMC6213009; doi:10.3390/md16100345)
Supplement: Supplementary file 1 [file marinedrugs-16-00345-s001.zip › marinedrugs-357309-supplementary.pdf]

Supplementary Information

Seco-tetracenomycins from the marine-derived actinomycete *Saccharothrix* sp. 10-10

Bin Liu [1,2,†], Jiao Li [1,†], Minghua Chen [1], Xiaomeng Hao [1], Fei Cao [3], Yi Tan [1], Yuhui Ping [2], Yiguang Wang [1], Chunling Xiao [1] and Maoluo Gan [1,*]

[1] Institute of Medicinal Biotechnology, Chinese Academy of Medical Sciences and Peking Union Medical College, Beijing 100050, China; bin0629bin@163.com (B.L.); jiaoli930911@126.com (J.L.); 15210165499@163.com (M.C.); xiaomhao@163.com (X.H.); tanyiyi123@126.com (Y.T.); 13552532543@163.com (Y.W.); xiaocl318@163.com (C.X.)

[2] College of Pharmacy, Jiangxi University of Traditional Chinese Medicine, Nanchang 330004, China; pingyh@163.com

[3] Key Laboratory of Medicinal Chemistry and Molecular Diagnostic of Ministry of Education, College of Pharmacy, Hebei University, Baoding 071002, China; caofei542927001@163.com

* Correspondence: ganml@imb.pumc.edu.cn; Tel.: +86-10-6316-5277

† These authors contributed equally to this work.

Supporting Information

Contents

Table S1. NMR Spectroscopic Data for **1** and **3–5** in DMSO-d_6 .... S4
Table S2. NMR Spectroscopic Data for 13-de-*O*-methyltetracenomycin X (**6**) and tetracenomycin X (**7**) in CD_3OD S5
Table S3. NMR Spectroscopic Data for Saccharothrixones I (**5**), B (**8**), and C (**9**).......................... S6
Figure S1. The experimental ECD and UV spectra of saccharothrixones E–I, B, C (**1–5**, **8**, **9**), 13-de-*O*-methyltetracenomycin X (**6**) and Tcm X (**7**) recorded in MeOH. S7
Scheme 1. Plausible biosynthesis pathway for saccharothrixones A, B (**8**), C (**9**) and E–I (**1–5**)..... S8
Figure S2. The (+)-HRESIMS spectrum of saccharothrixone E (**1**)... S9
Figure S3. The IR spectrum of saccharothrixone E (**1**). S10
Figure S4. The ^{1}H NMR spectrum of saccharothrixone E (**1**) in acetone-d_6 (500 MHz). S11
Figure S5. The ^{1}H NMR spectrum of saccharothrixone E (**1**) in DMSO-d_6 (600 MHz)................ S12
Figure S6. The ^{13}C NMR spectrum of saccharothrixone E (**1**) in acetone-d_6 (125 MHz). S13
Figure S7. The HSQC spectrum of saccharothrixone E (**1**) in acetone-d_6 (500 MHz)................... S14
Figure S8. The HMBC spectrum of saccharothrixone E (**1**) in acetone-d_6 (500 MHz).................. S15
Figure S9. The ROESY spectrum of saccharothrixone E (**1**) in acetone-d_6 (500 MHz). S16
Figure S10. The ROESY spectrum of saccharothrixone E (**1**) in DMSO-d_6 (600 MHz). S17
Figure S11. The (+)-HRESIMS spectrum of saccharothrixone F (**2**).. S18
Figure S12. The IR spectrum of saccharothrixone F (**2**).. S19
Figure S13. The ^{1}H NMR spectrum of saccharothrixone F (**2**) in acetone-d_6 (600 MHz). S20
Figure S14. The ^{13}C NMR spectrum of saccharothrixone F (**2**) in acetone-d_6 (150 MHz). S21
Figure S15. The HSQC spectrum of saccharothrixone F (**2**) in acetone-d_6 (600 MHz). S22
Figure S16. The HMBC spectrum of saccharothrixone F (**2**) in acetone-d_6 (600 MHz)................ S23
Figure S17. The (–)-HRESIMS spectrum of saccharothrixone G (**3**). S24
Figure S18. The IR spectrum of saccharothrixone G (**3**).. S25
Figure S19. The ^{1}H NMR spectrum of saccharothrixone G (**3**) in acetone-d_6 (600 MHz)............. S26
Figure S20. The ^{1}H NMR spectrum of saccharothrixone G (**3**) in DMSO-d_6 (600 MHz). S27
Figure S21. The ^{13}C NMR spectrum of saccharothrixone G (**3**) in acetone-d_6 (150 MHz)............ S28
Figure S22. The HSQC spectrum of saccharothrixone G (**3**) in acetone-d_6 (600 MHz). S29
Figure S23. The HMBC spectrum of saccharothrixone G (**3**) in acetone-d_6 (600 MHz). S30
Figure S24. The ROESY spectrum of saccharothrixone G (**3**) in acetone-d_6 (600 MHz).............. S31
Figure S25. The1D NOE spectrum of saccharothrixone G (**3**) in DMSO-d_6 (600 MHz)............... S32
Figure S26. The1D NOE spectrum of saccharothrixone G (**3**) in DMSO-d6 (600 MHz).............. S33
Figure S27. The (–)-HRESIMS spectrum of saccharothrixone H (**4**). S34
Figure S28. The IR spectrum of saccharothrixone H (**4**).. S35
Figure S29. The ^{1}H NMR spectrum of saccharothrixone H (**4**) in acetone-d_6 (600 MHz)............. S36
Figure S30. The ^{13}C NMR spectrum of saccharothrixone H (**4**) in acetone-d_6 (150 MHz)............ S37
Figure S31. The HSQC spectrum of saccharothrixone H (**4**) in acetone-d_6 (600 MHz). S38
Figure S32. The HMBC spectrum of saccharothrixone H (**4**) in acetone-d_6 (600 MHz). S39
Figure S33. The ROESY spectrum of saccharothrixone H (**4**) in acetone-d_6 (600 MHz).............. S40

Figure S34. The (–)-HRESIMS spectrum of saccharothrixone I (**5**). S41
Figure S35. The IR spectrum of saccharothrixone I (**5**). S42
Figure S36. The ^{1}H NMR spectrum of saccharothrixone I (**5**) in acetone-d_6 (500 MHz). S43
Figure S37. The 1H NMR spectrum of saccharothrixone I (**5**) in DMSO-d6 (600 MHz)........ S44
Figure S38. The ^{13}C NMR spectrum of saccharothrixone I (**5**) in acetone-d_6 (125 MHz). S45
Figure S39. The HSQC spectrum of saccharothrixone I (**5**) in acetone-d_6 (500 MHz). S46
Figure S40. The HMBC spectrum of saccharothrixone I (**5**) inacetone-d_6 (500 MHz). S47
Figure S41. The ROESY spectrum of saccharothrixone I (**5**) in acetone-d_6 (600 MHz). S48
Figure S42. The ROESY spectrum of saccharothrixone I (**5**) in DMSO-d_6 (600 MHz). S49
Figure S43. The (+)-HR ESIMS spectrum of 13-de-*O*-methyltetracenomycin X (**6**). S50
Figure S44. The IR spectrum of 13-de-*O*-methyltetracenomycin X (**6**). S51
Figure S45. The ^{1}H NMR spectrum of 13-de-*O*-methyltetracenomycin X (**6**) in CD_3OD. S52
Figure S46. The ^{13}C NMR spectrum of 13-de-*O*-methyltetracenomycin X (**6**) in CD_3OD. S53
Figure S47. The HSQC spectrum of 13-de-*O*-methyltetracenomycin X (**6**) in CD_3OD S54
Figure S48. The HMBC spectrum of 13-de-*O*-methyltetracenomycin X (**6**) in CD_3OD. S55
Figure S49. Optimized conformers (≥ 1%) of (4*S*,4a*R*,5*S*,12a*R*)-**1a** at the B3LYP/6-311+G(d,p) level with PCM model in MeOH. S56
Figure S50. Optimized conformers (≥ 1%) of (4*S*,4a*R*,5*S*,12a*R*)-**2a** at the B3LYP/6-311+G(d,p) level with PCM model in MeOH. S56
Figure S51. Optimized conformers (≥ 1%) of (4*S*,4a*R*,5*S*,12a*S*)-**3a** at the B3LYP/6-311+G(d,p) level with PCM model in MeOH. S56
Figure S52. Optimized conformers (≥ 1%) of (4*R*,4a*R*,5*S*,12a*S*)-**3c** at the B3LYP/6-311+G(d,p) level with PCM model in MeOH. S56
Figure S53. Optimized conformers (≥ 1%) of (4*S*,4a*R*,5*R*,12a*R*)-**5a** at the B3LYP/6-311+G (d,p) level with PCM model in MeOH. S57

Table S1. NMR Spectroscopic Data for **1** and **3**–**5** in DMSO-d_6[a]

no	**1** (DMSO-d_6)	**3** (DMSO-d_6)		**4** (DMSO-d_6)		**5** (DMSO-d_6)	
No.	δ_H, mult. (J in Hz)	δ_C	δ_H, mult. (J in Hz)	δ_C	δ_H, mult. (J in Hz)	δ_C	δ_H, mult. (J in Hz)
1		171.9		172.0		194.8	
2	5.62, s	88.6	5.38, d (1.2)	88.3	5.42, s	99.1	5.29, d (1.2)
3		183.4		181.3		173.2	
4	4.54, d (3.0)	77.0	5.16, d (1.2)	79.1	6.10, s	67.6	4.54, d (7.8)
4a		81.3		77.7		80.0	
5	4.58, d (3.0)	67.6	4.85, d (10.2)	66.5	5.18, d (9.6)	79.1	5.81, d (1.2)
5a		140.6		141.0		143.1	
6	7.41, s	116.7	7.42, s	116.6	7.42, s	113.8	7.52, d (1.2)
6a		140.6		141.0		141.2	
7	7.31, s	105.1	7.26, s	105.4	7.30, s	105.4	7.25, s
8		156.2		156.5		155.0	
9		126.0		125.9		125.9	
10		135.9		136.0		134.8	
10a		116.4		116.4		117.3	
11		163.9		164.0		156.9	
11a		108.6		107.8		105.6	
12		202.8		203.0		170.9	
12a		83.7	4.42, s	81.6	3.56, s	83.6	3.82, s
13		167.8		167.7		167.9	
14	2.73, s	20.5	2.72, s	20.6	2.72, s	20.4	2.73, s
3-OCH_3	3.83, s	59.7	3.84, s	59.8	3.87, s	56.4	3.64, s
8-OCH_3	3.89, s	56.1	3.90, s	56.2	3.91, s	55.9	3.88, s
12a-OCH_3	3.60, s	60.6	3.63, s	58.4	3.25, s	59.7	3.36, s
13-OCH_3	3.86, s	52.4	3.86, s	52.4	3.86, s	52.4	3.86, s
4-OH	5.47, d (3.0)						5.37, d (7.8)
4a-OH	5.23, s		5.26, s		5.31, s		5.74, s
5-OH	6.07, brs		5.81, d (10.2)		5.86, d (9.6)		
11-OH	14.70, brs		14.48, brs		n.o.		

[a] ^{1}H NMR and ^{13}C NMR data were recorded at 600 and 150 MHz, respectively. The assignments were based on HSQC and HMBC experiments. n.o.: not observed.

Table S2. NMR Spectroscopic Data for 13-de-*O*-methyltetracenomycin X (**6**) and tetracenomycin X (**7**) in CD_3OD [a]

No.	**6** (CD_3OD)		**7** (CD_3OD)	
	δ_C, type	δ_H, m	δ_C	δ_H, m
1	193.9, C		188.3	
2	101.9, CH	5.53, d (1.8)	101.4	5.32, brs
3	174.8, C		178.8	
4	70.9, CH	4.85, brs	70.2	4.88, brs
4a	86.1, C		84.8	
5	194.4, C		195.4	
5a	141.9, C		141.4	
6	121.8, CH	8.01, s	115.8	7.42, s
6a	128.7, C		127.2	
7	108.7, CH	7.46, s	108.0	7.20, s
8	159.3, C		157.8	
9	132.8, C		130.6	
10	138.0, C		140.2	
10a	121.9, C		127.2	
11	167.5, C		170.1	
11a	110.5, C		112.1	
12	197.6, C		195.6	
12a	89.0, C		88.9	
13	171.8, C		173.5	
14	21.1, CH_3	2.88, s	20.5	2.63, s
3-OCH_3	57.5, CH_3	3.80, s	56.4	3.85, s
8-OCH_3	56.7, CH_3	4.01, s	55.7	3.91, s
12a-OCH_3	56.7, CH_3	3.56, s	55.6	3.48, s
13-OCH_3			52.2	3.58, s

Table S3. NMR Spectroscopic Data for Saccharothrixones I (**5**), B (**8**), and C (**9**) [a]

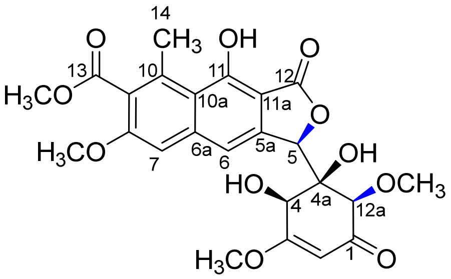

5

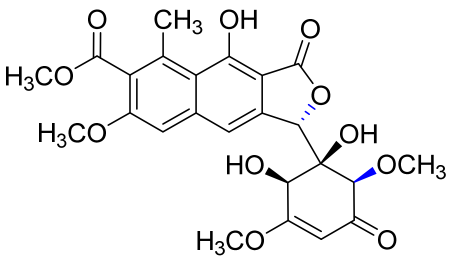

8

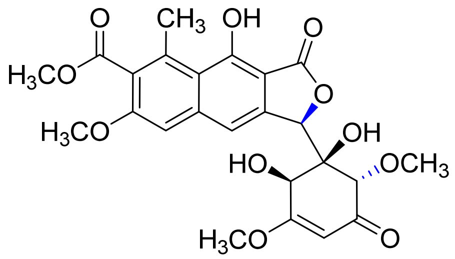

9

No.	**5** (acetone-d_6)		**8** (acetone-d_6)[b]		**9** (acetone-d_6)[b]	
	δ_C, type	δ_H, m	δ_C, type	δ_H, m	δ_C, type	δ_H, m
1	194.9, C		196.0, C		193.6, C	
2	100.3, CH	5.32, d (1.0)	100.7, CH	5.26, s	100.6, CH	5.30, s
3	173.1, C		174.4, C		174.0, C	
4	68.3, CH	4.65, d (1.0)	72.2, CH	3.88, s	69.0, CH	4.62, s
4a	80.3, C		77.1, C		78.4, C	
5	81.0, CH	5.98, s	83.1, CH	5.81, s	84.7, CH	5.92, s
5a	143.0, C		142.1, C		142.4, C	
6	115.0, CH	7.61, s	114.3, CH	7.57, s	115.1, CH	7.61, s
6a	143.3, C		142.8, C		143.1, C	
7	106.2, CH	7.27, s	106.3, CH	7.37, s	106.3, CH	7.32, s
8	156.9, C		156.8, C		157.0, C	
9	127.7, C		127.7, C		127.9, C	
10	136.1, C		136.0, C		136.2, C	
10a	118.0, C		117.7, C		118.0, C	
11	158.5, C		158.2, C		158.6, C	
11a	106.2, C		107.7, C		105.9, C	
12	173.4, C		173.7, C		173.3, C	
12a	84.1, CH	3.93, s	81.6, CH	4.42, s	83.8, CH	3.84, s
13	168.6, C		168.7, C		168.5, C	
14	20.4, CH_3	2.81, s	20.4, CH_3	2.82, s	20.4, CH_3	2.81, s
3-OCH_3	56.8, CH_3	3.72, s	56.7, CH_3	3.65, s	56.9, CH_3	3.75, s
8-OCH_3	56.3, CH_3	3.93, s	56.4, CH_3	3.97, s	56.4, CH_3	3.97, s
12a-OCH_3	60.6, CH_3	3.51, s	61.1, CH_3	3.73, s	59.4, CH_3	3.28, s
13-OCH_3	52.5, CH_3	3.90, s	52.5, CH_3	3.91, s	52.5, CH_3	3.91, s

[a] ^{1}H NMR data were measured for **5, 8** and **9** at 500, 800, and 600 MHz, respectively. ^{13}C NMR data were measured for for **5, 8** and **9** at 125, 200, and 1500 MHz. The assignments were based on HSQC and HMBC experiments. [b] The NMR data were reported in our previous work: Gan, M. et al. *J. Nat. Prod*. 2015, 78, 2260-2265.

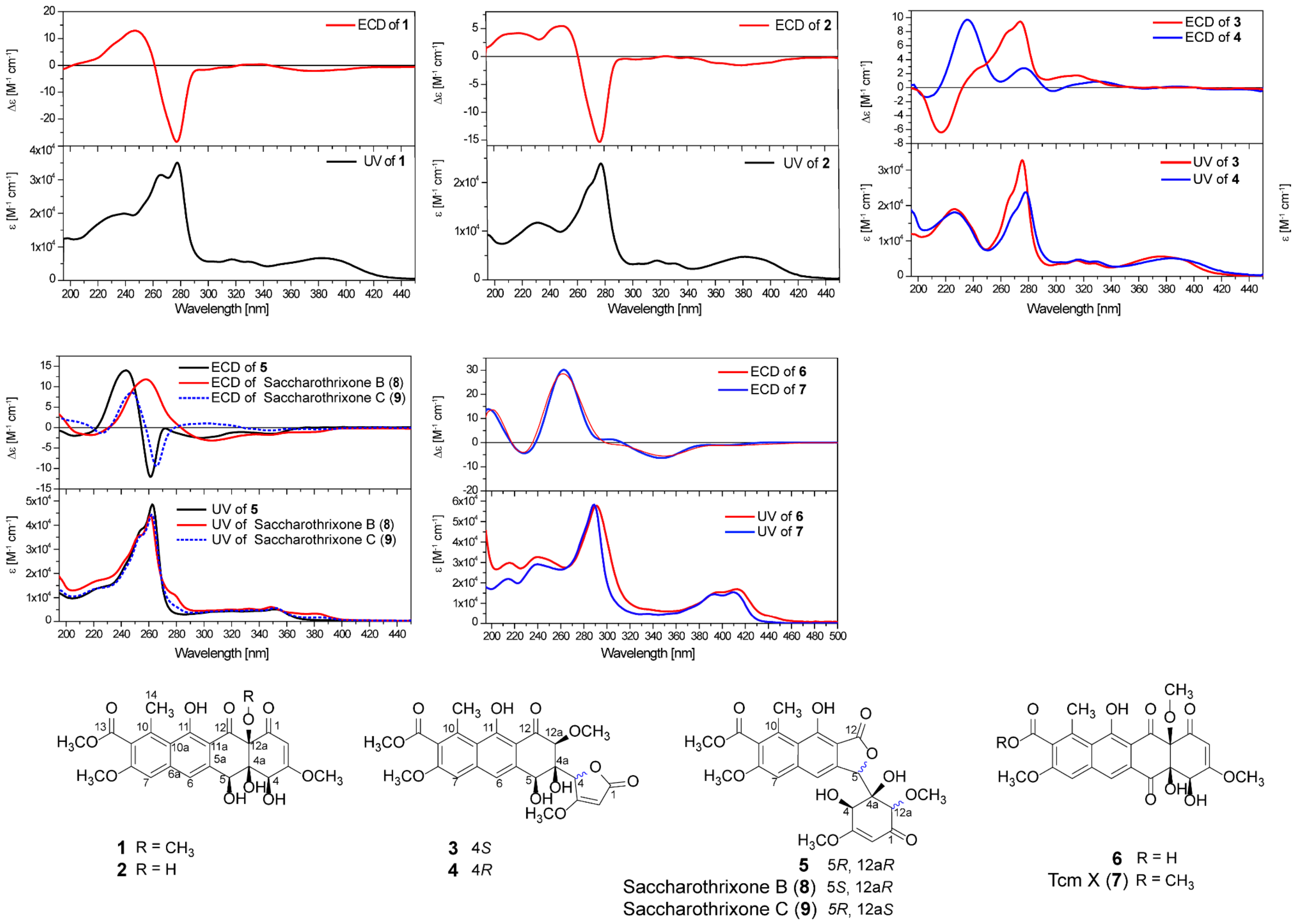

Figure S1. The experimental ECD and UV spectra of saccharothrixones E–I, B, C (**1**–**5**, **8**, **9**), 13-de-*O*-methyltetracenomycin X (**6**) and Tcm X (**7**) recorded in MeOH.

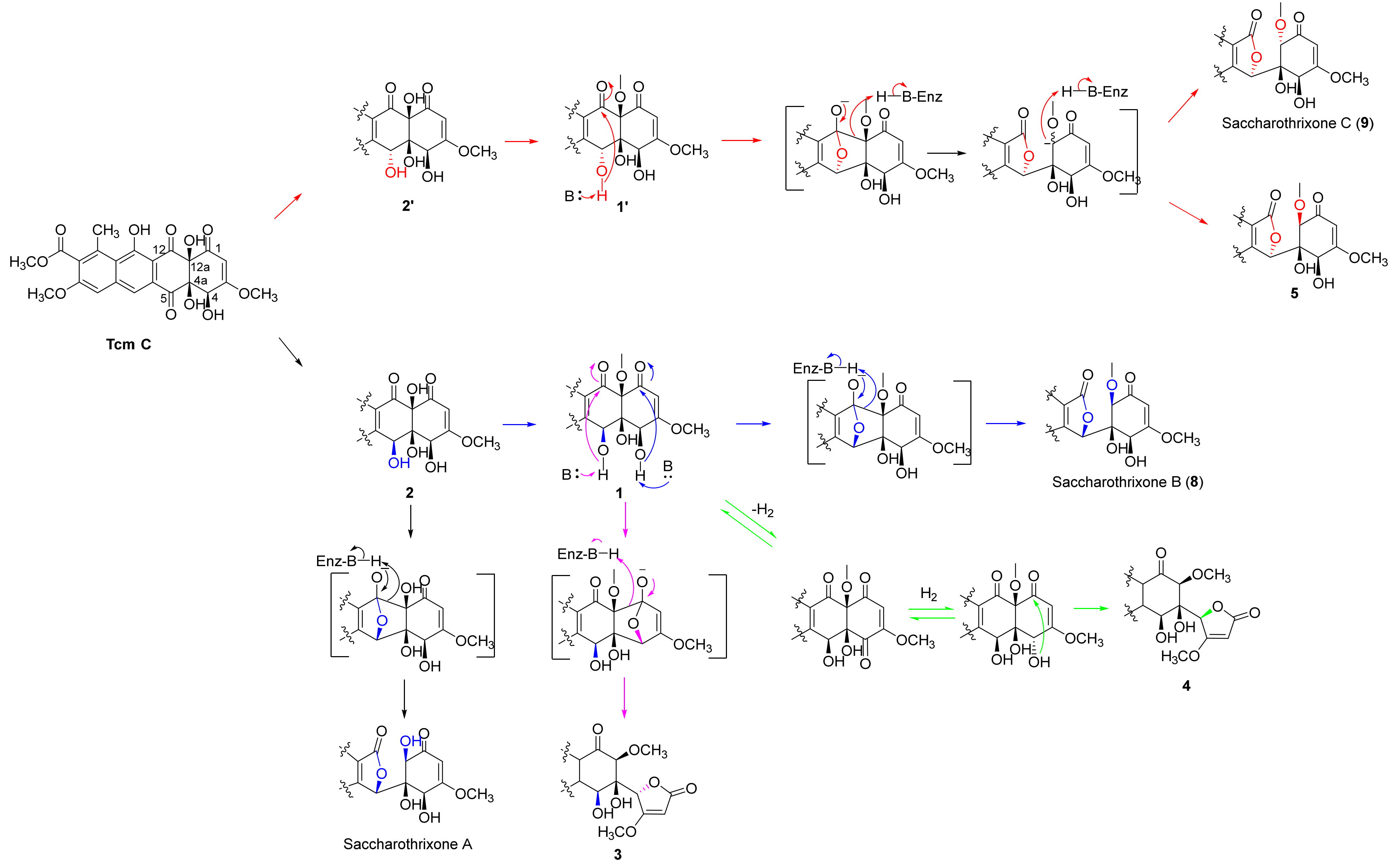

Scheme 1. Plausible biosynthesis pathway for saccharothrixones A, B (**8**), C (**9**) and E–I (**1**–**5**).

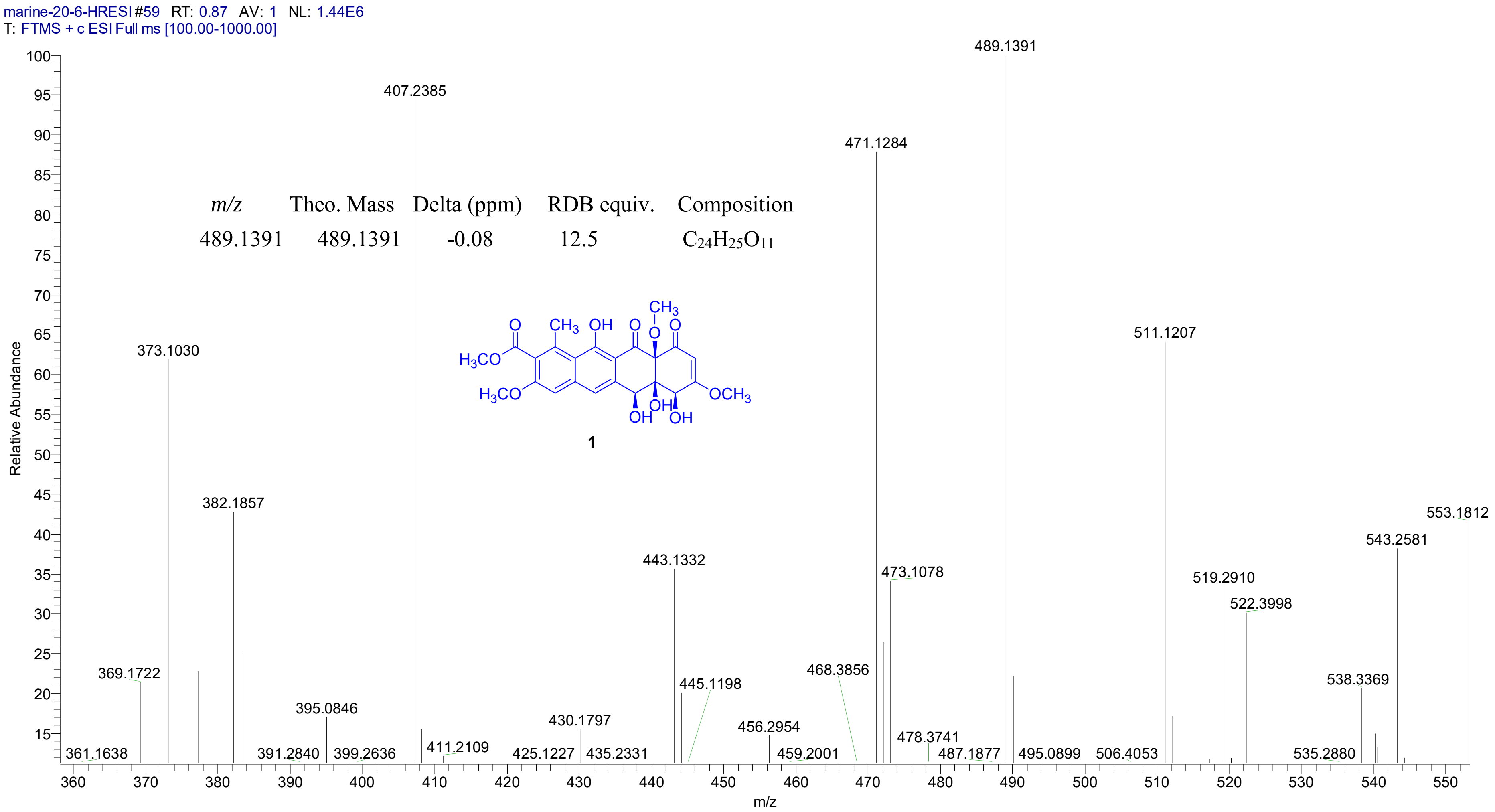

Figure S2. The (+)-HRESIMS spectrum of saccharothrixone E (**1**).

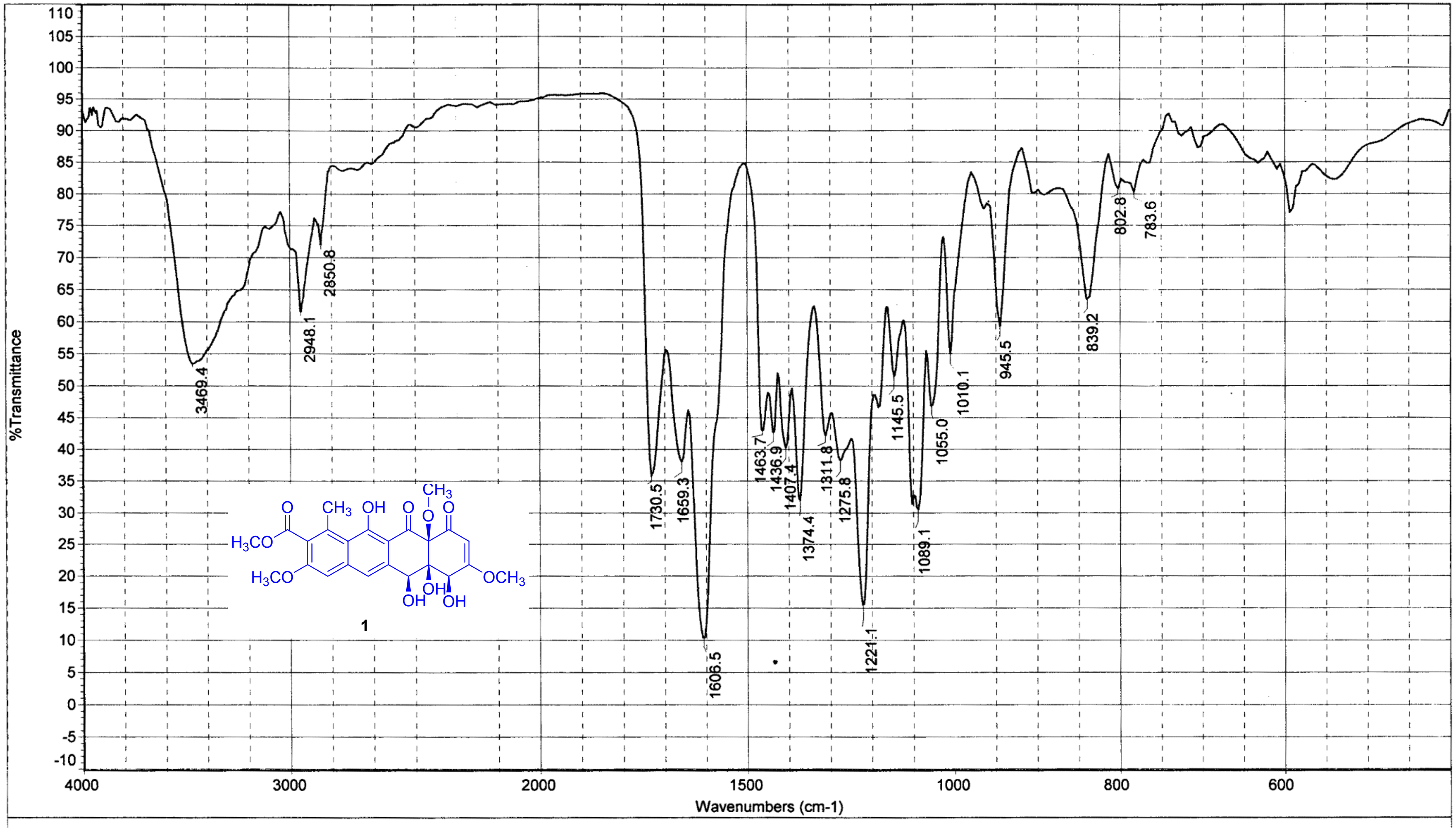

Figure S3. The IR spectrum of saccharothrixone E (**1**).

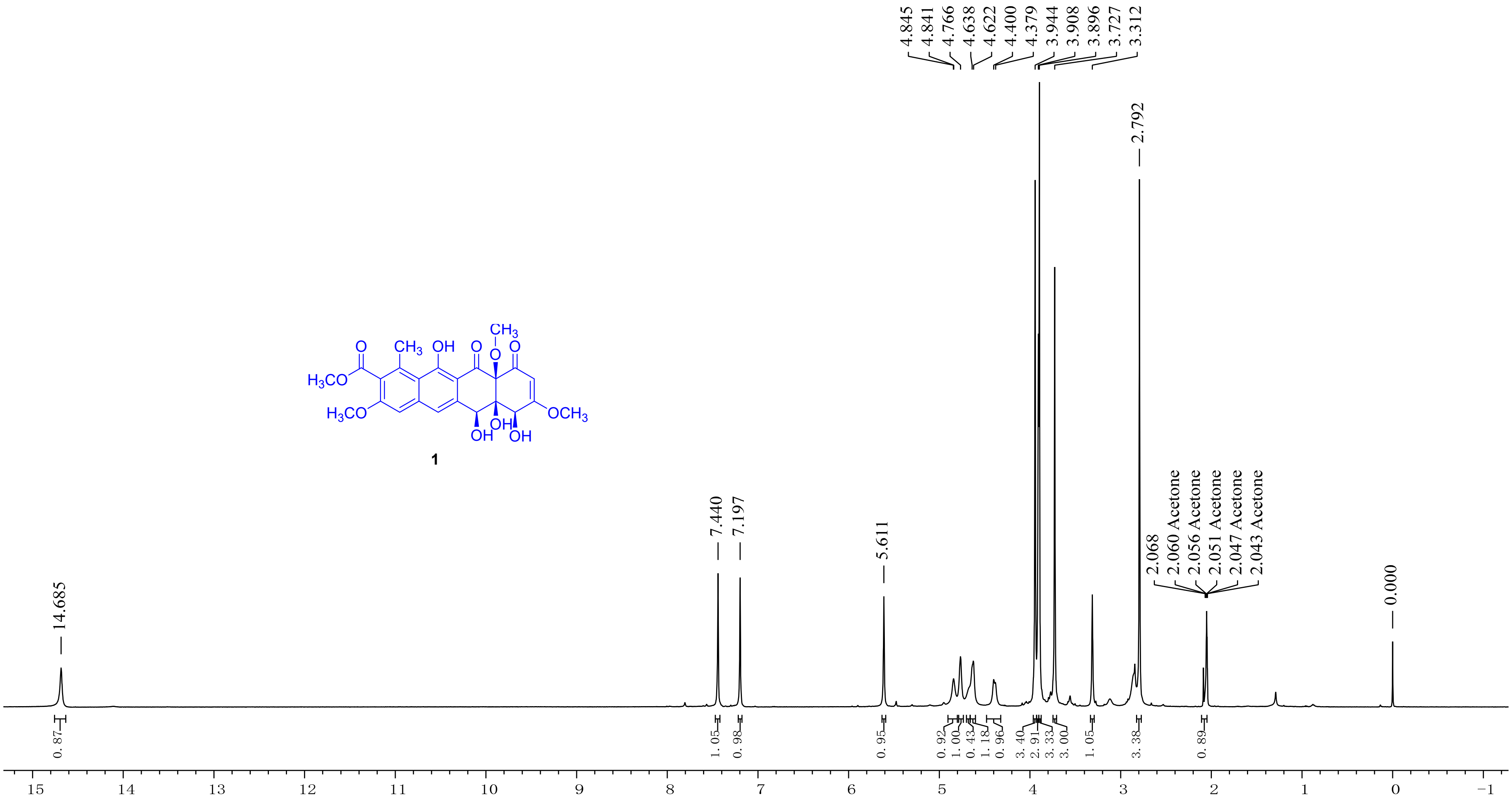

Figure S4. The ^{1}H NMR spectrum of saccharothrixone E (**1**) in acetone-d_6 (500 MHz).

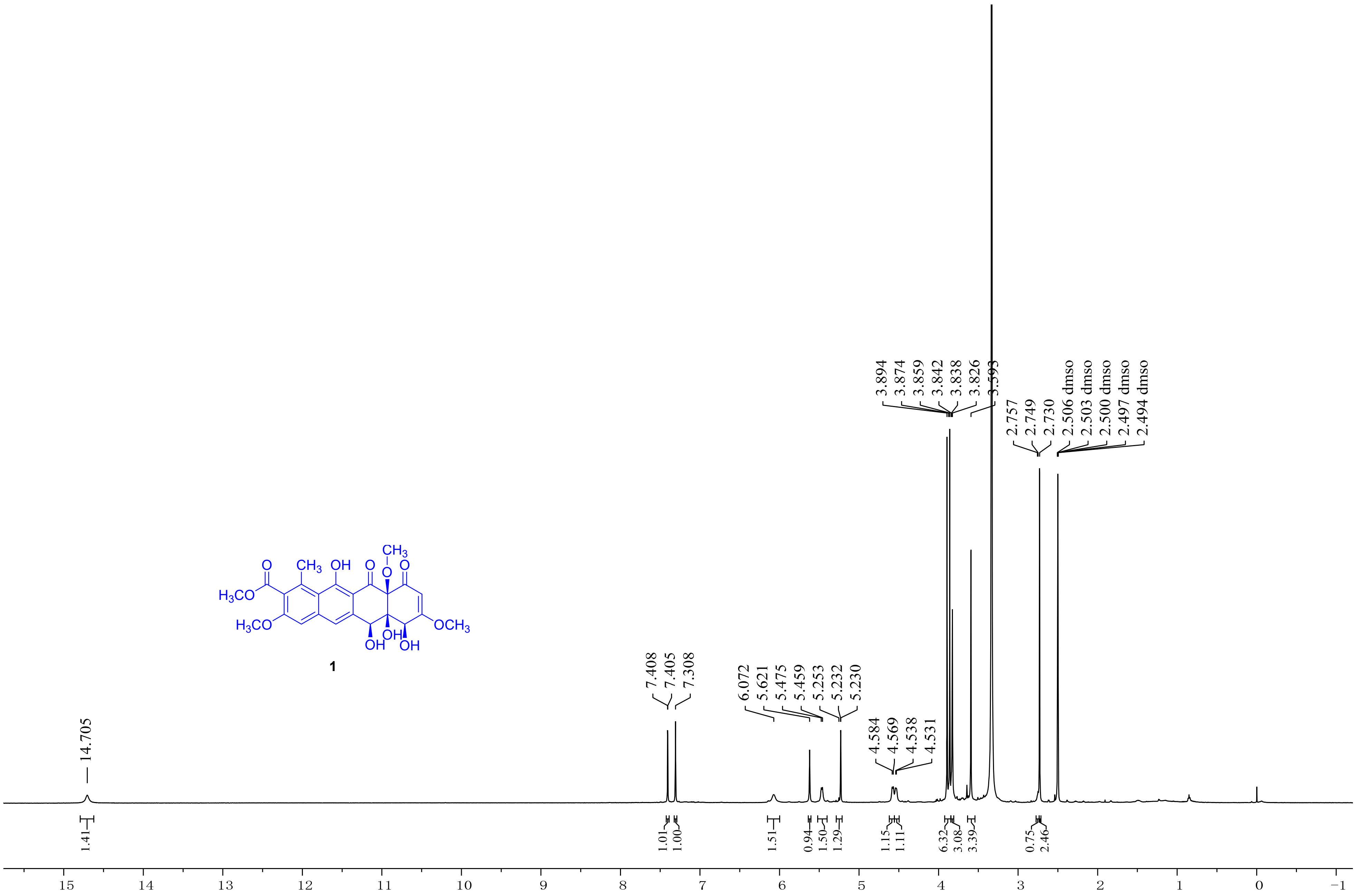

Figure S5. The ^{1}H NMR spectrum of saccharothrixone E (**1**) in DMSO-d_6 (600 MHz).

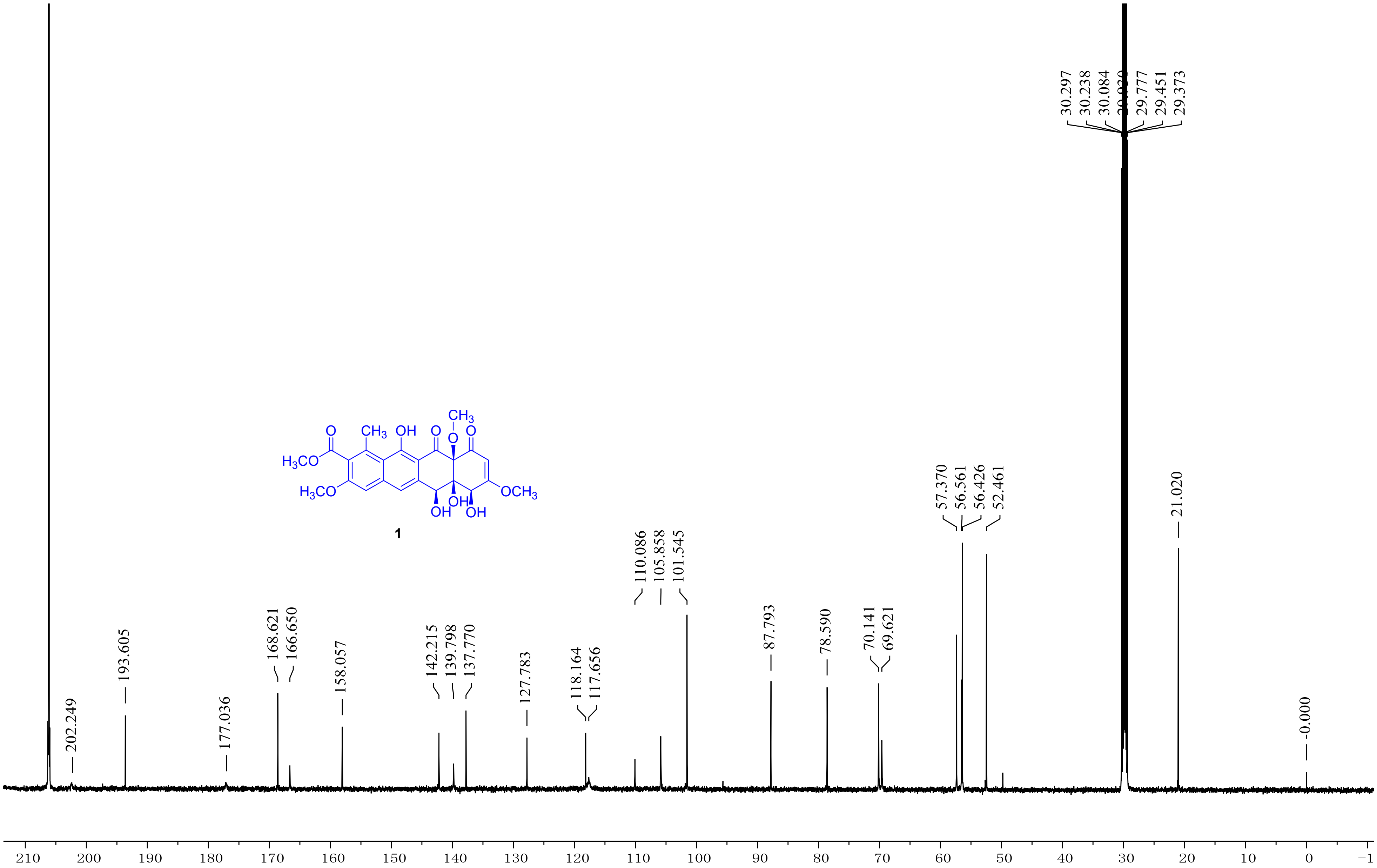

Figure S6. The ${}^{13}C$ NMR spectrum of saccharothrixone E (**1**) in acetone-d_6 (125 MHz).

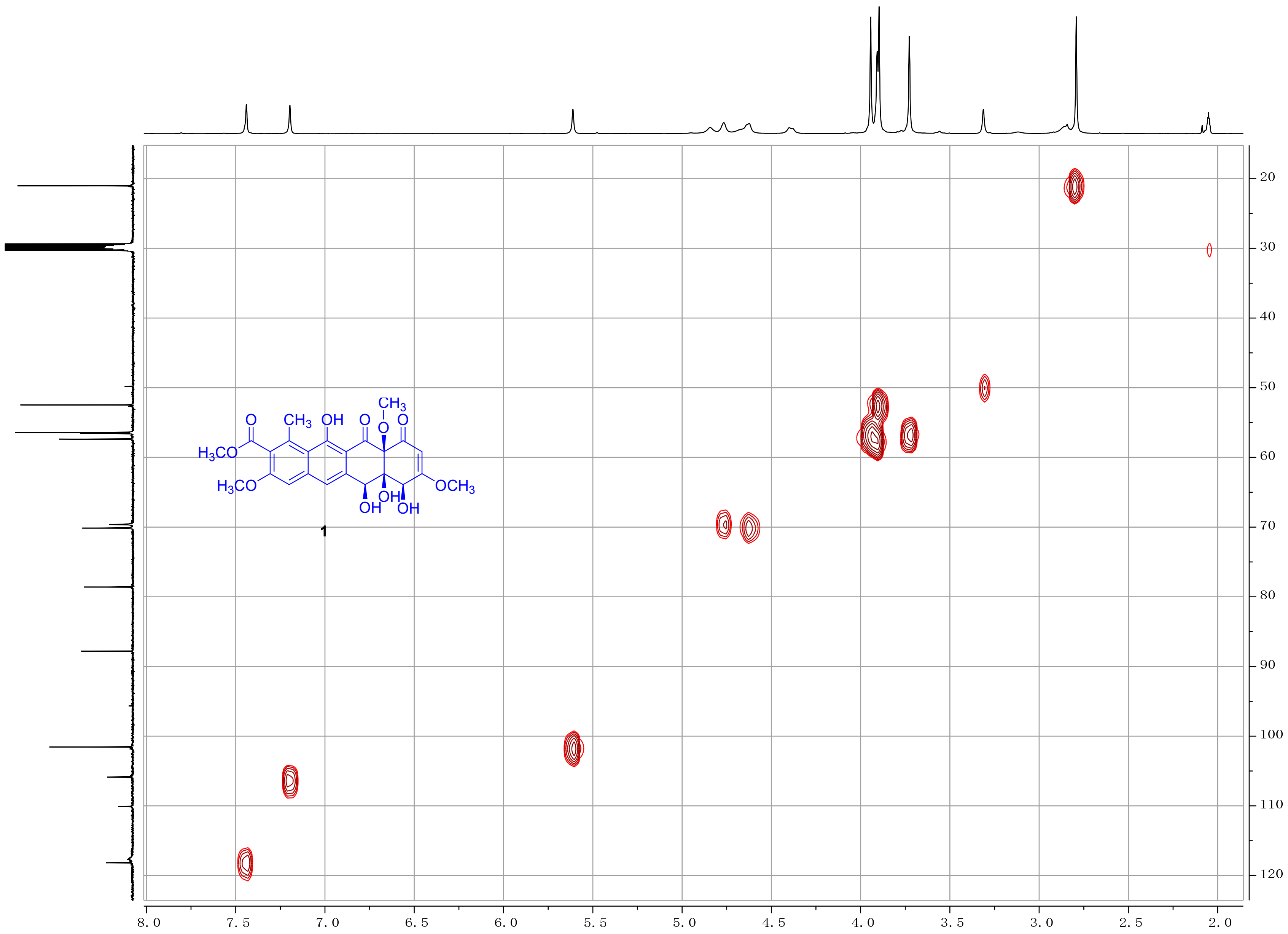

Figure S7. The HSQC spectrum of saccharothrixone E (**1**) in acetone-d_6 (500 MHz).

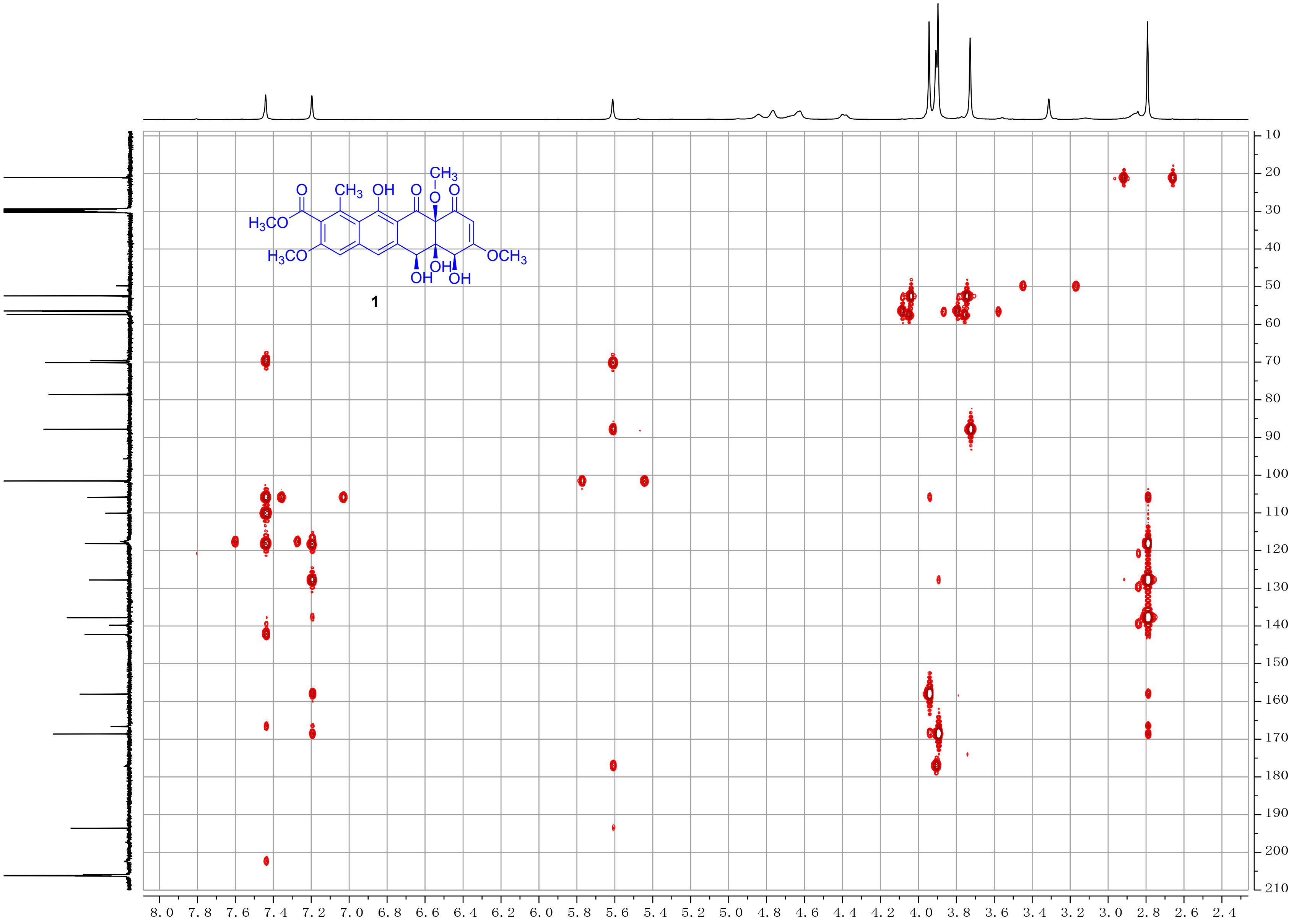

Figure S8. The HMBC spectrum of saccharothrixone E (**1**) in acetone-d_6 (500 MHz).

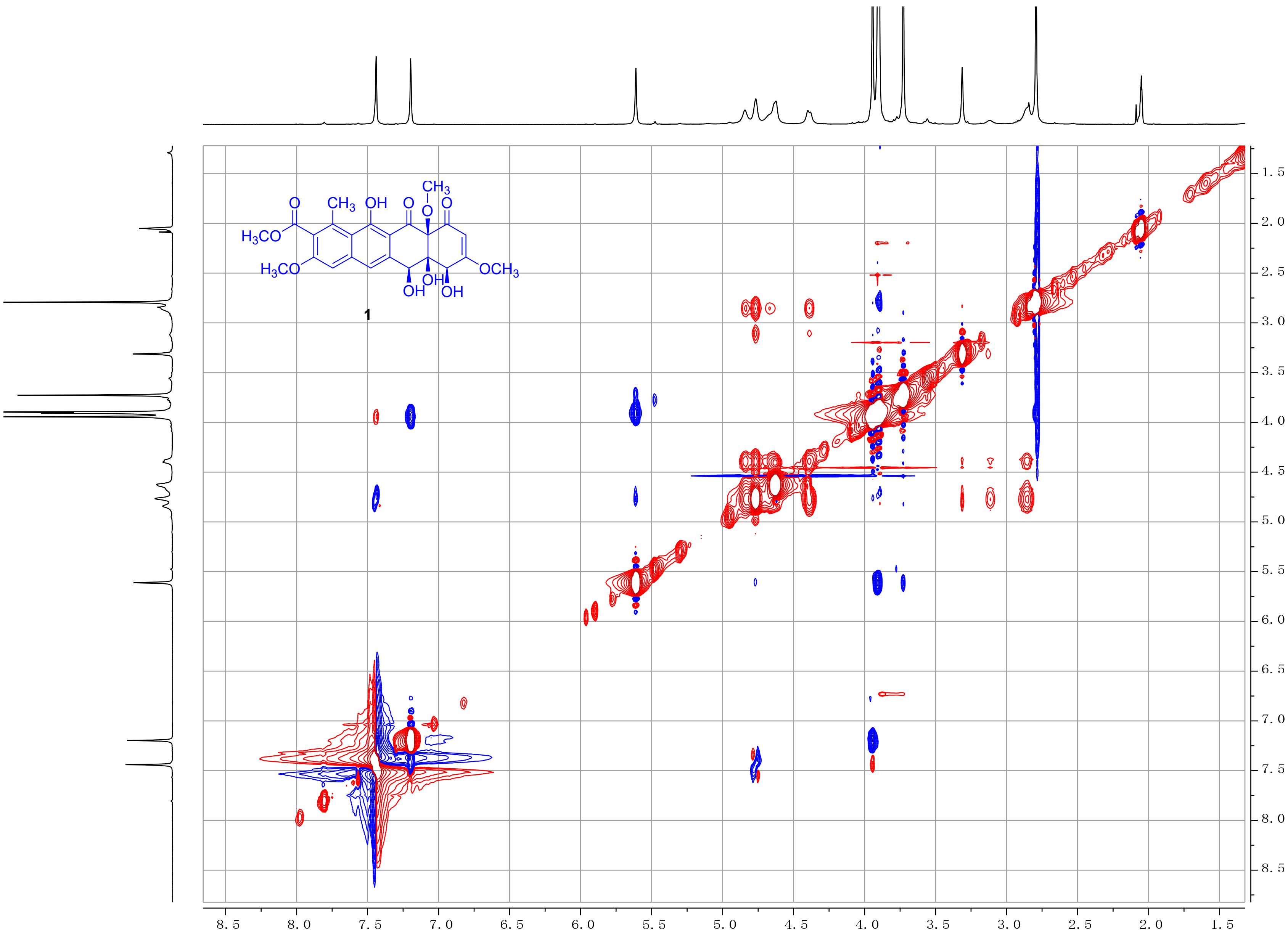

Figure S9. The ROESY spectrum of saccharothrixone E (**1**) in acetone-d_6 (500 MHz).

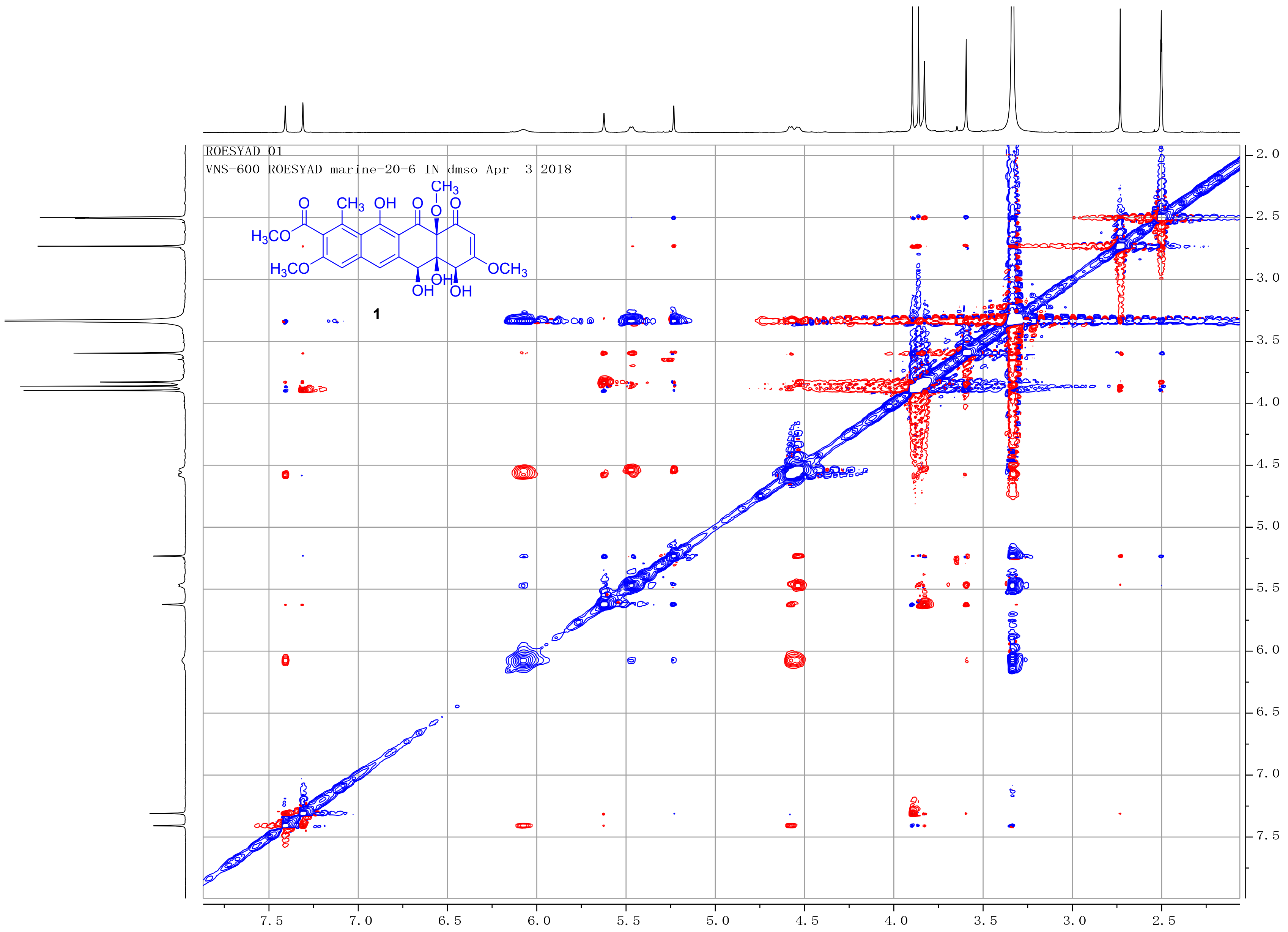

Figure S10. The ROESY spectrum of saccharothrixone E (**1**) in DMSO-d_6 (600 MHz).

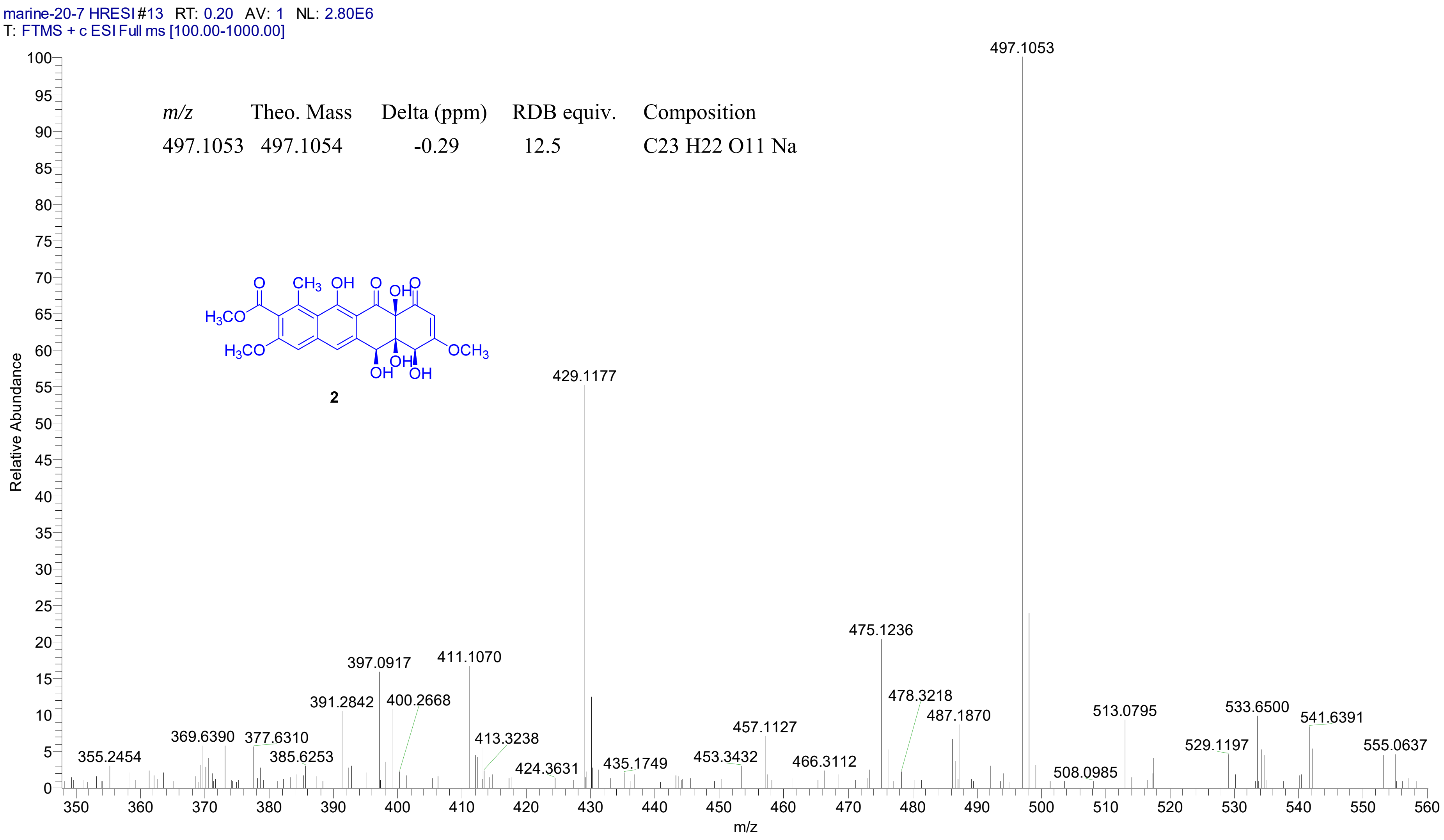

m/z	Theo. Mass	Delta (ppm)	RDB equiv.	Composition
497.1053	497.1054	-0.29	12.5	C23 H22 O11 Na

Figure S11. The (+)-HRESIMS spectrum of saccharothrixone F (**2**).

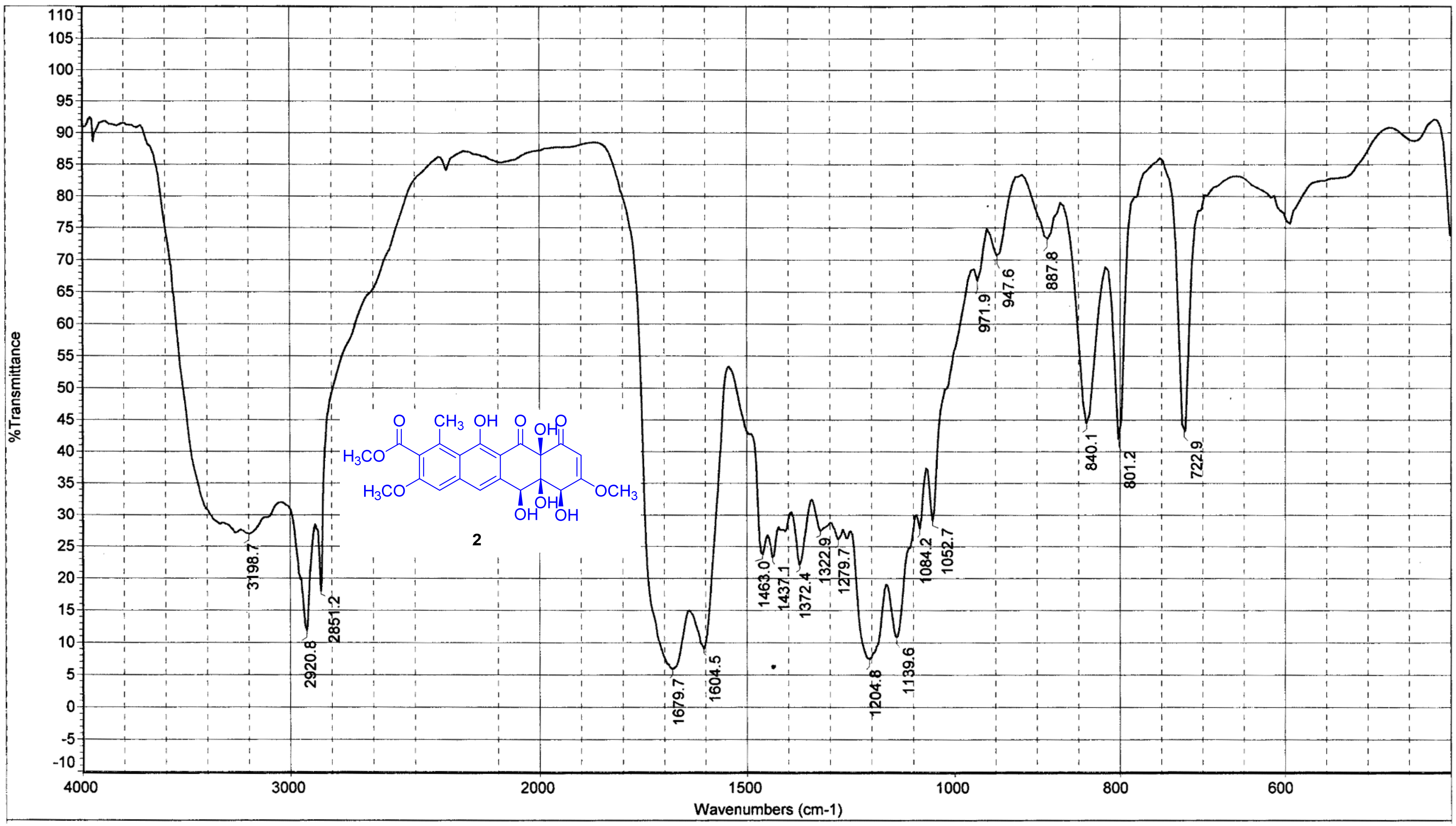

Figure S12. The IR spectrum of saccharothrixone F (**2**).

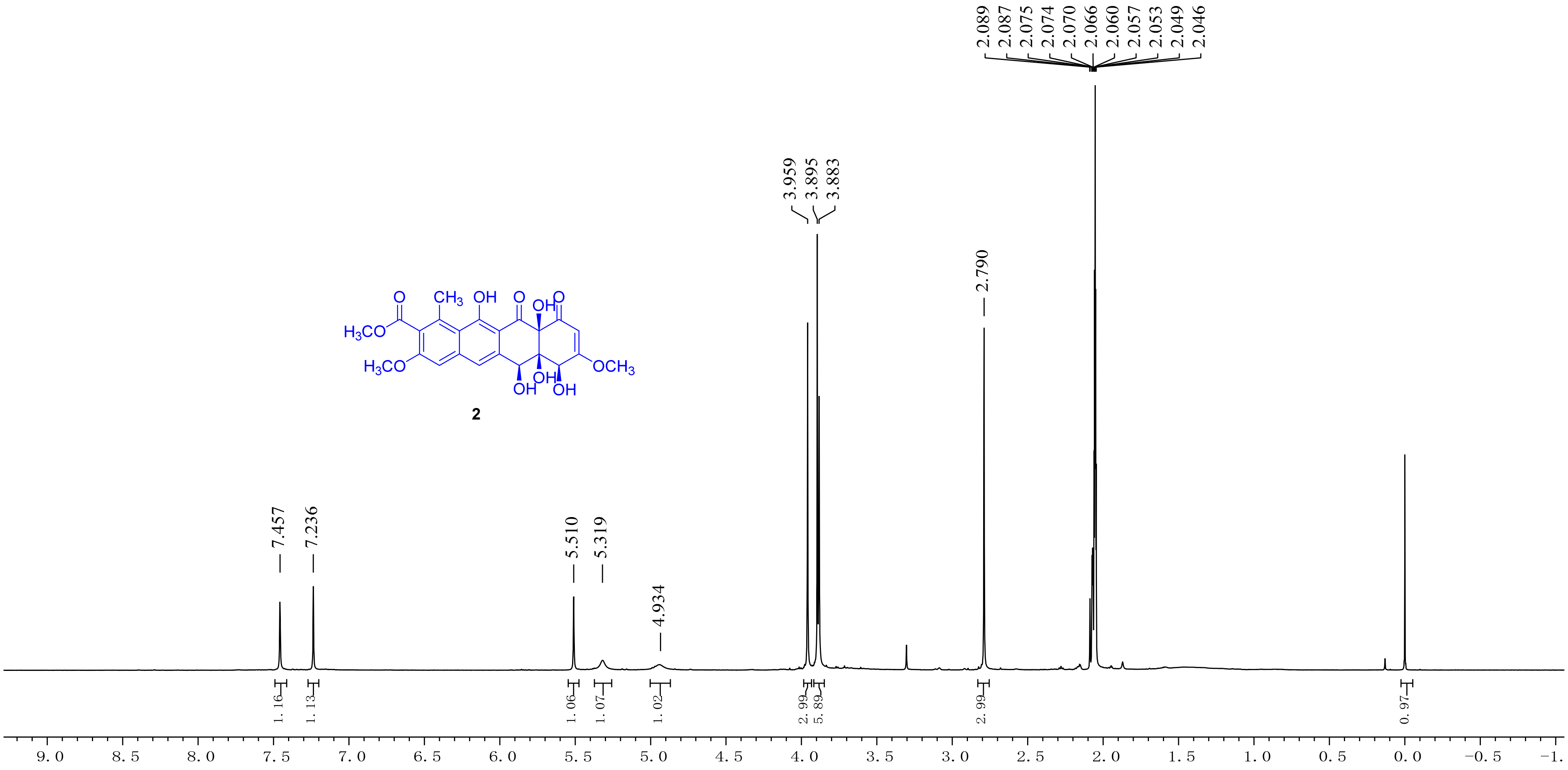

Figure S13. The ^{1}H NMR spectrum of saccharothrixone F (**2**) in acetone-d_6 (600 MHz).

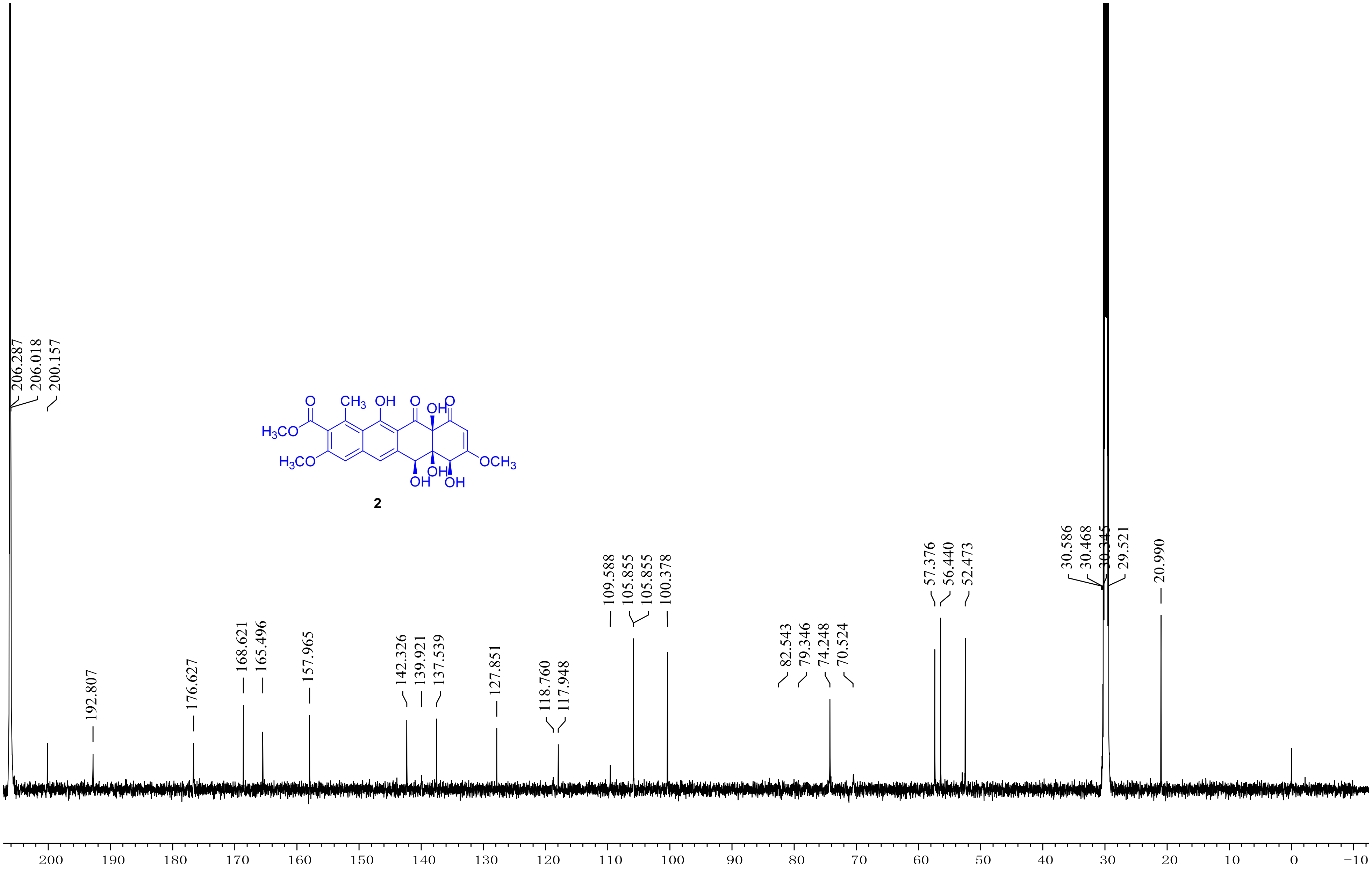

Figure S14. The ^{13}C NMR spectrum of saccharothrixone F (**2**) in acetone-d_6 (150 MHz).

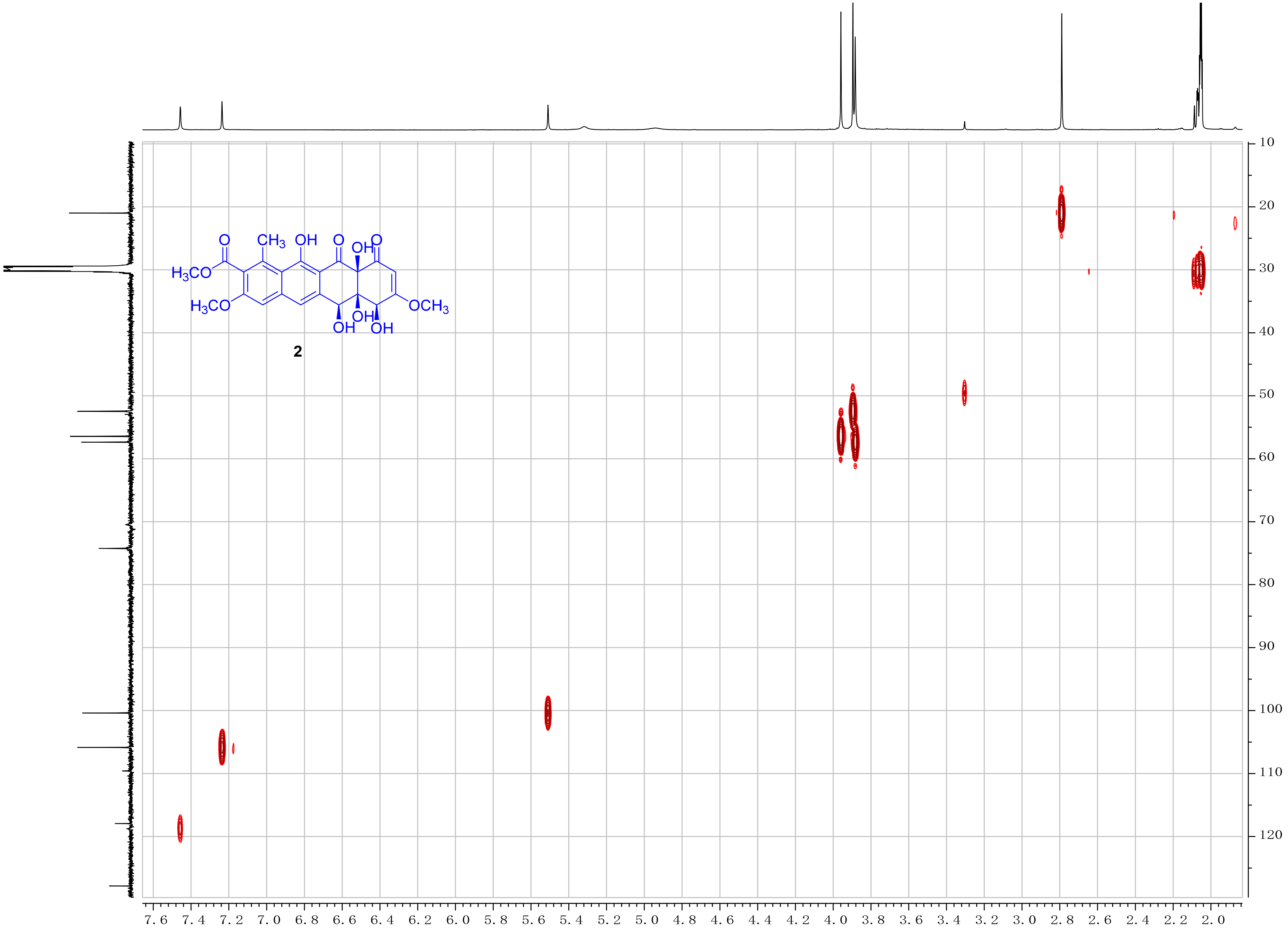

Figure S15. The HSQC spectrum of saccharothrixone F (**2**) in acetone-d_6 (600 MHz).

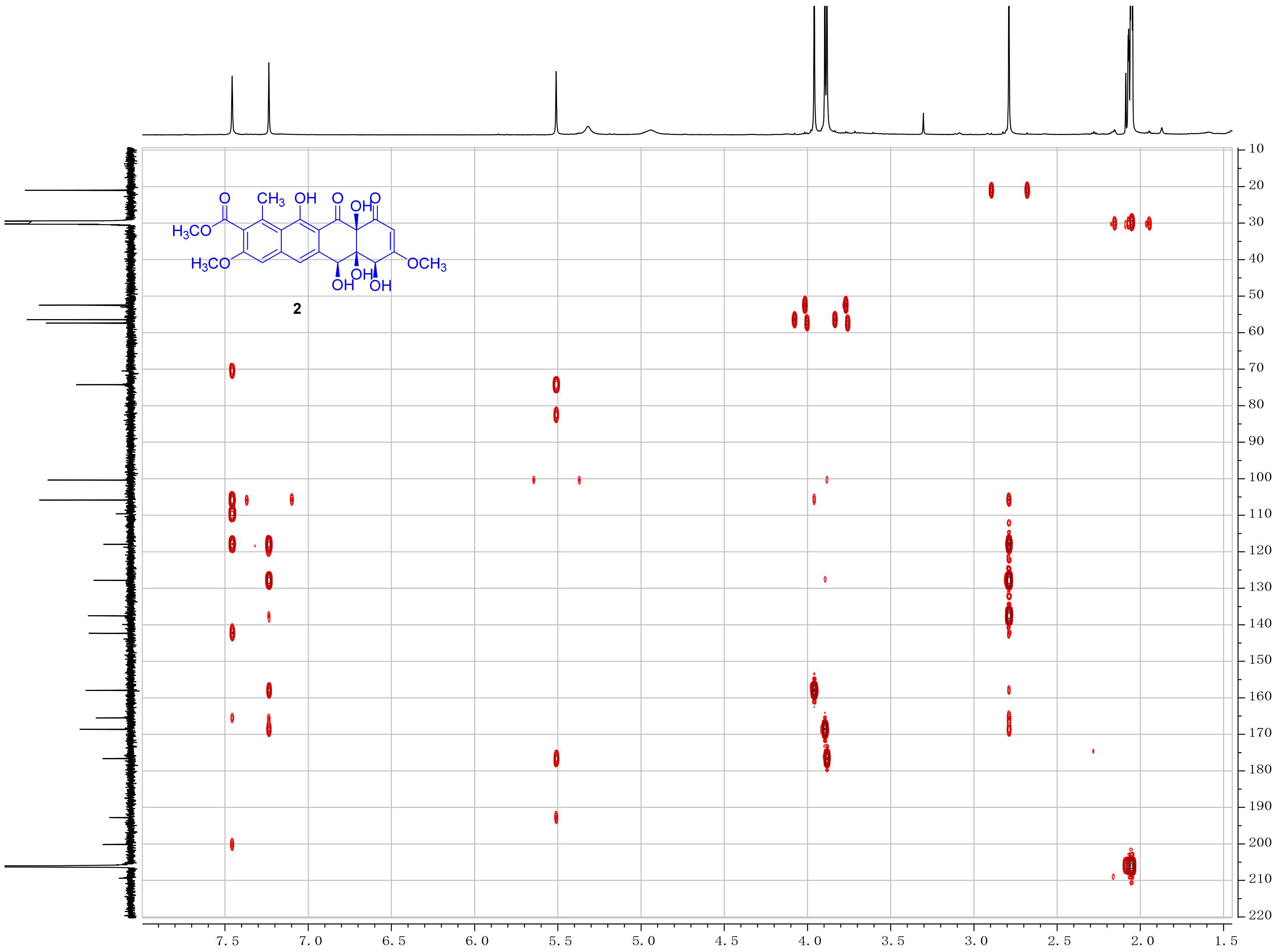

Figure S16. The HMBC spectrum of saccharothrixone F (**2**) in acetone-d_6 (600 MHz).

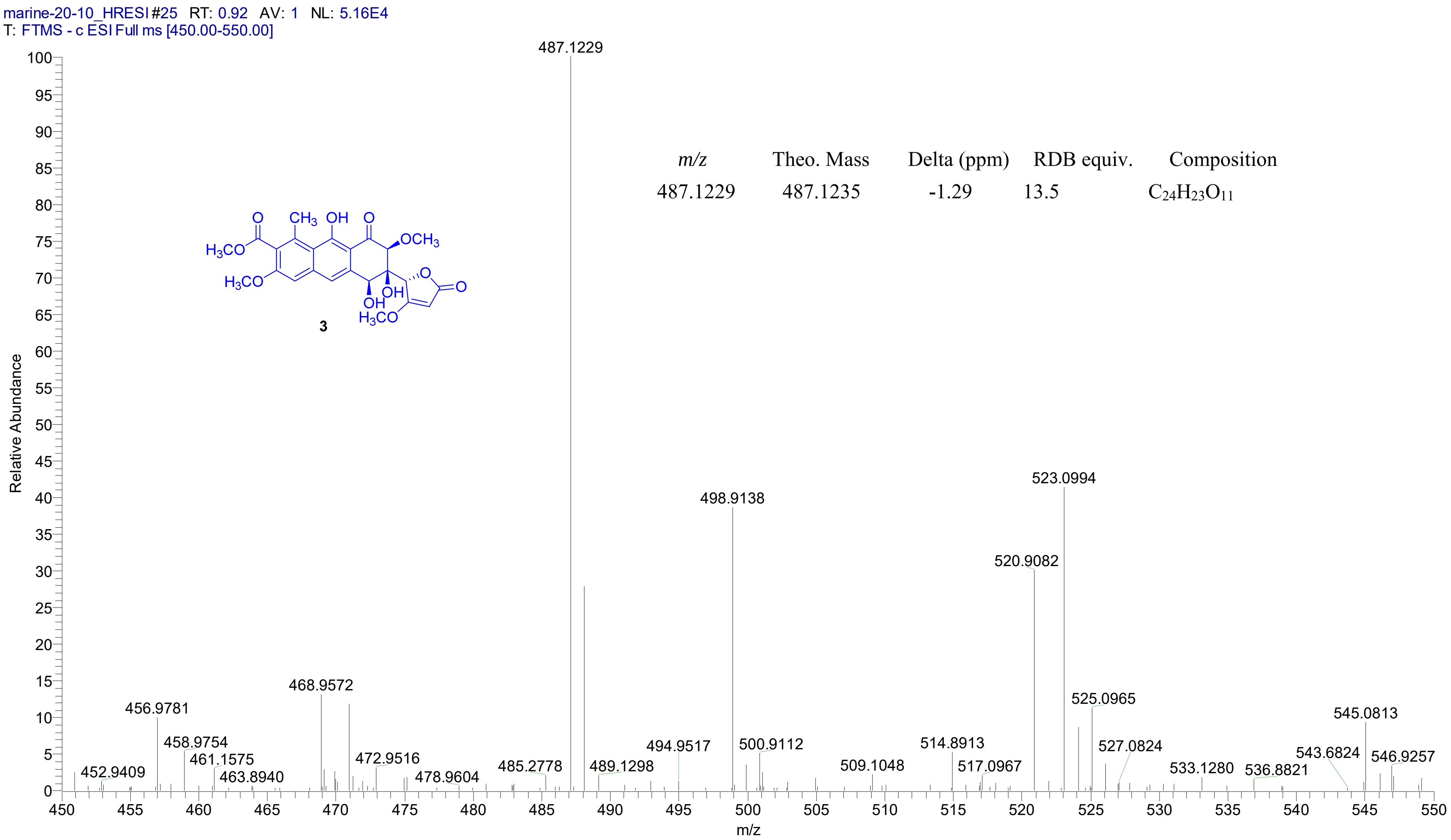

m/z	Theo. Mass	Delta (ppm)	RDB equiv.	Composition
487.1229	487.1235	-1.29	13.5	$C_{24}H_{23}O_{11}$

Figure S17. The (−)-HRESIMS spectrum of saccharothrixone G (**3**).

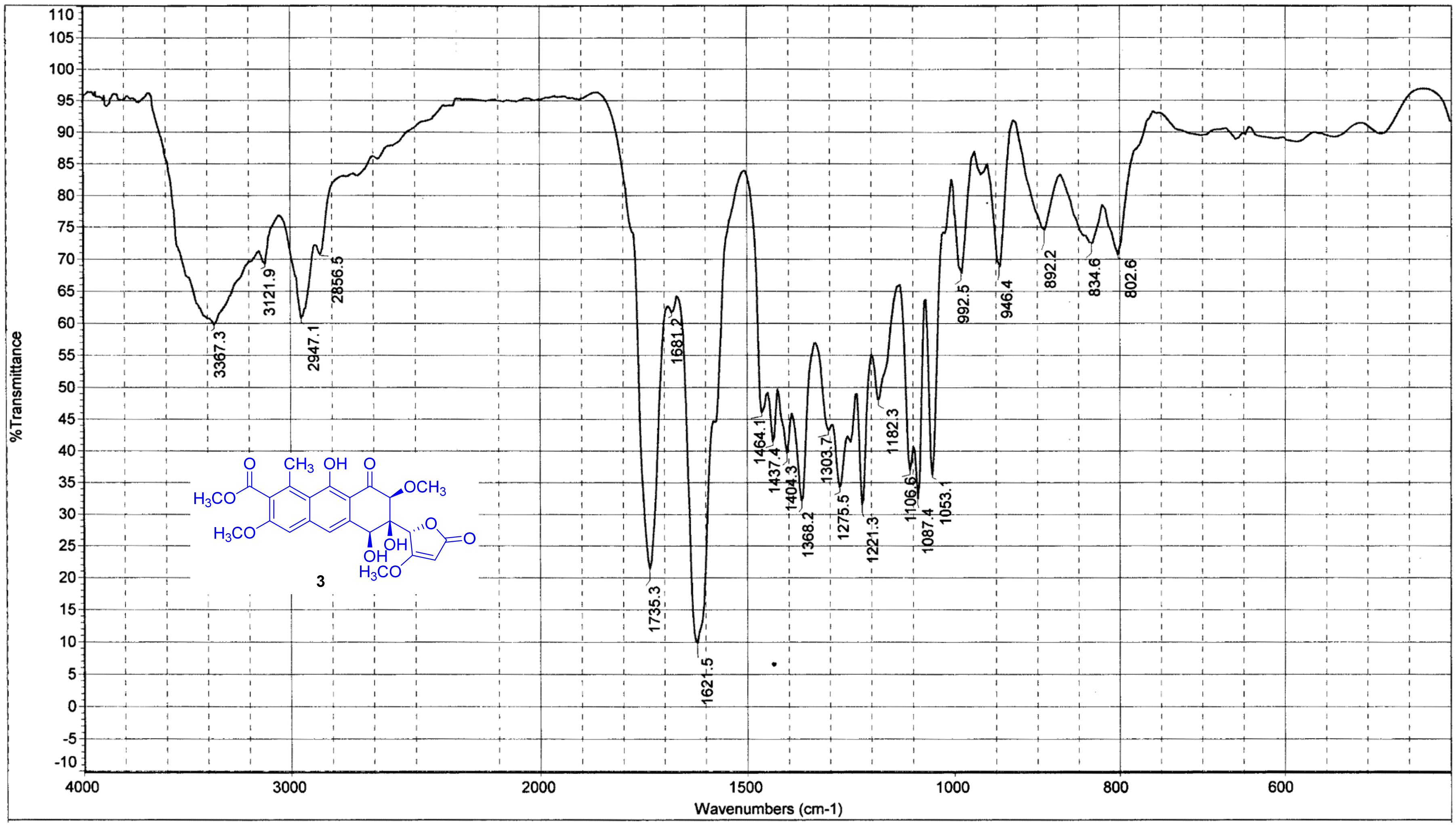

Figure S18. The IR spectrum of saccharothrixone G (**3**).

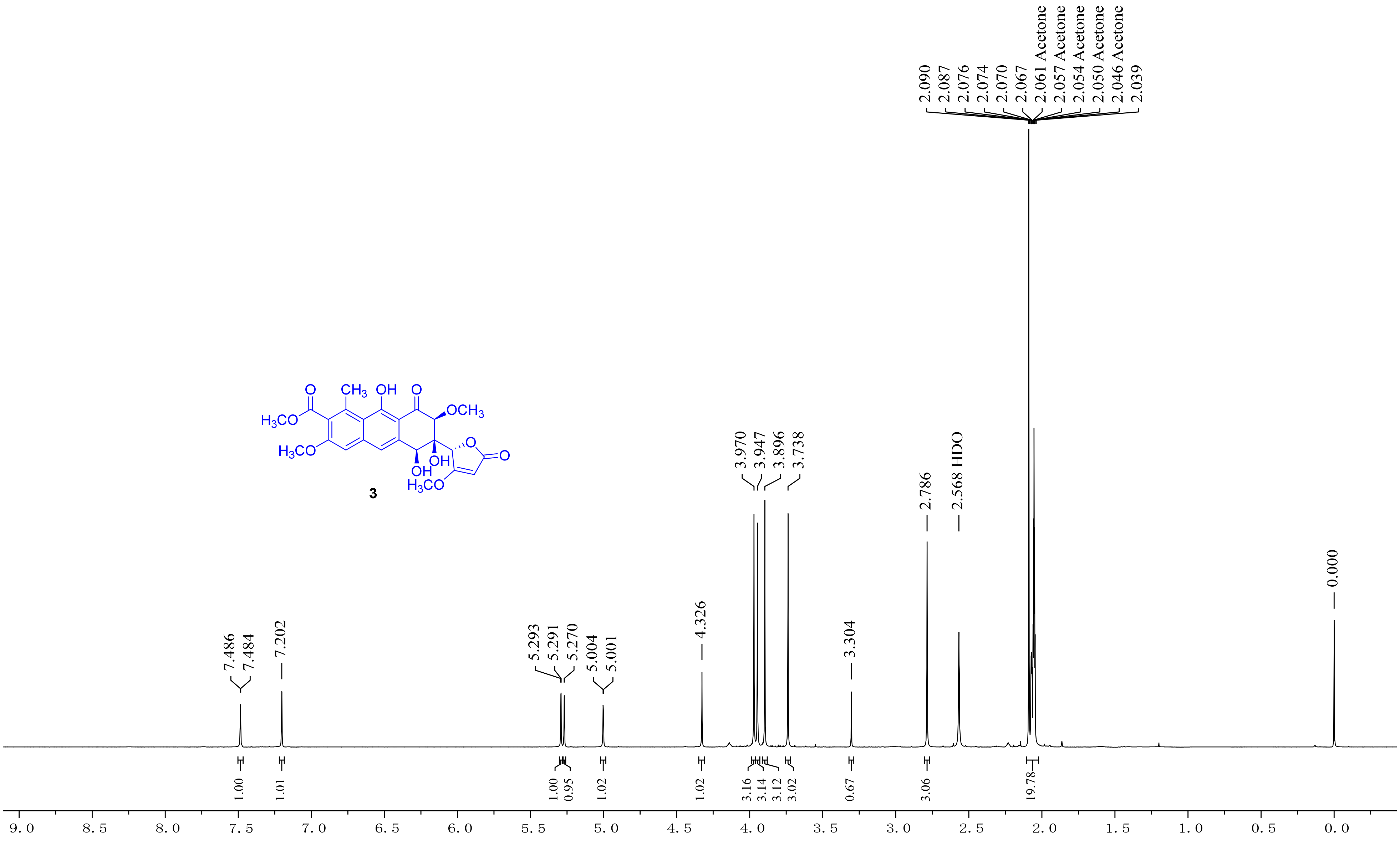

Figure S19. The ^{1}H NMR spectrum of saccharothrixone G (**3**) in acetone-d_6 (600 MHz).

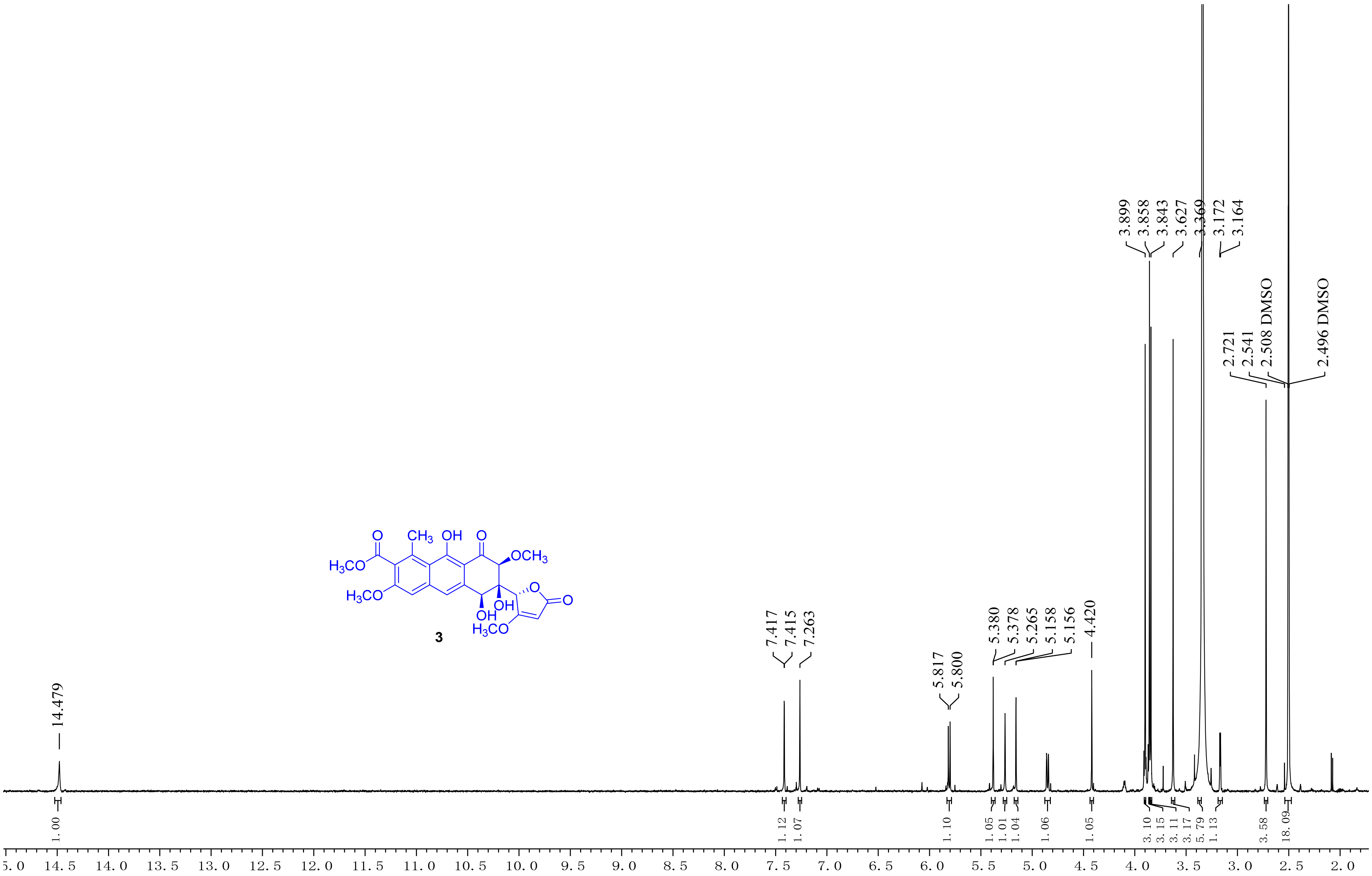

Figure S20. The ^{1}H NMR spectrum of saccharothrixone G (**3**) in DMSO-d_6 (600 MHz).

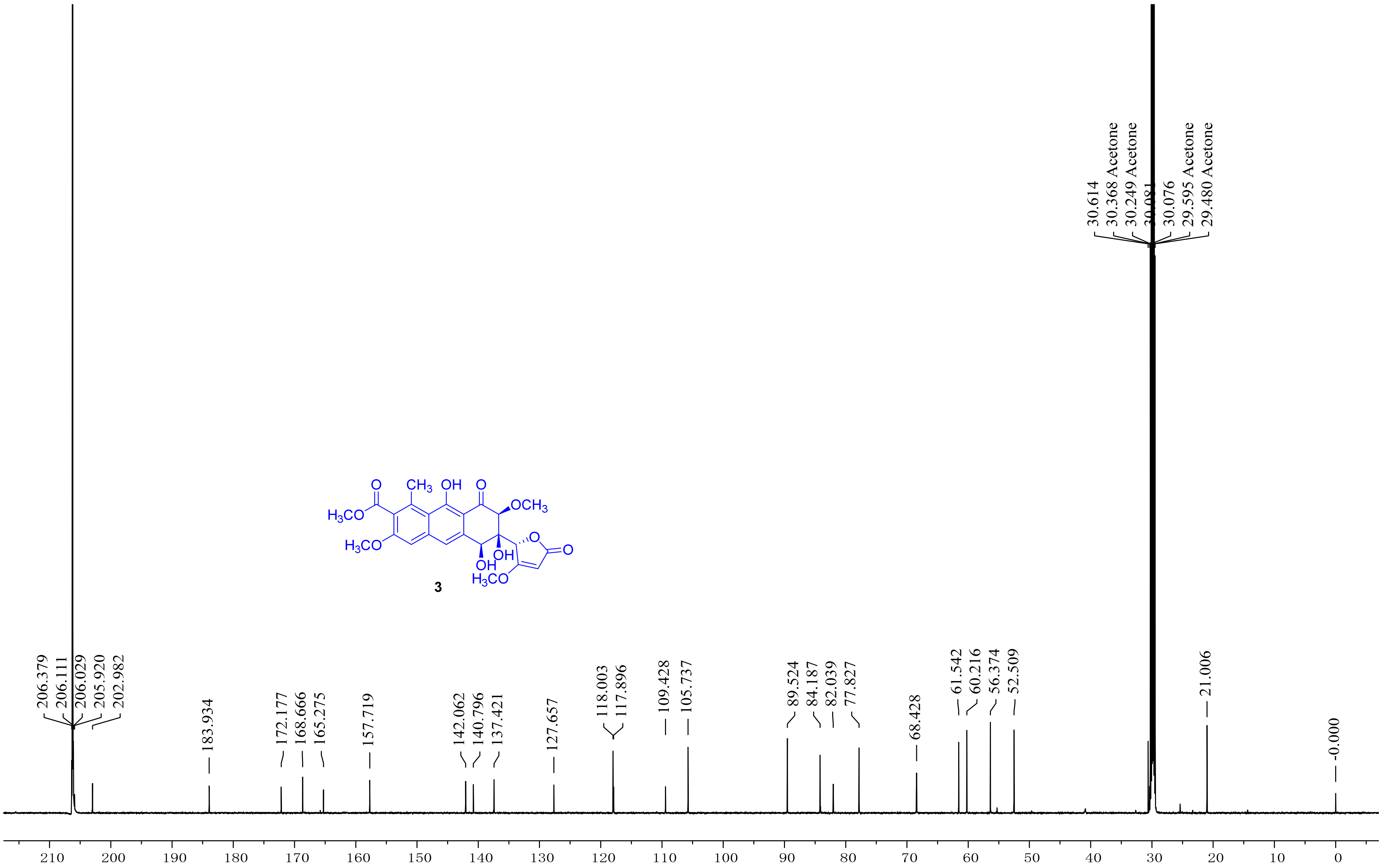

Figure S21. The ^{13}C NMR spectrum of saccharothrixone G (**3**) in acetone-d_6 (150 MHz).

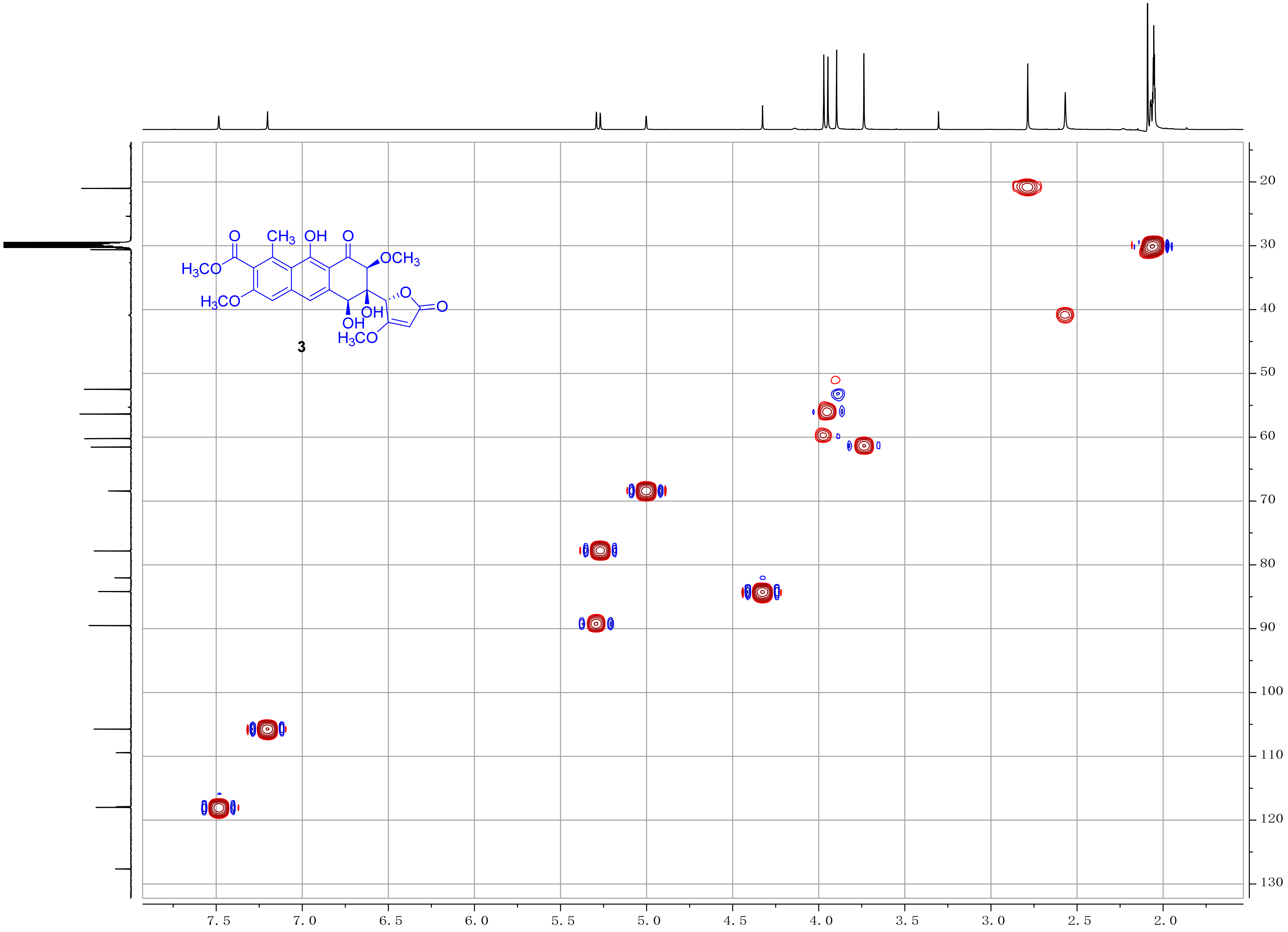

Figure S22. The HSQC spectrum of saccharothrixone G (**3**) in acetone-d_6 (600 MHz).

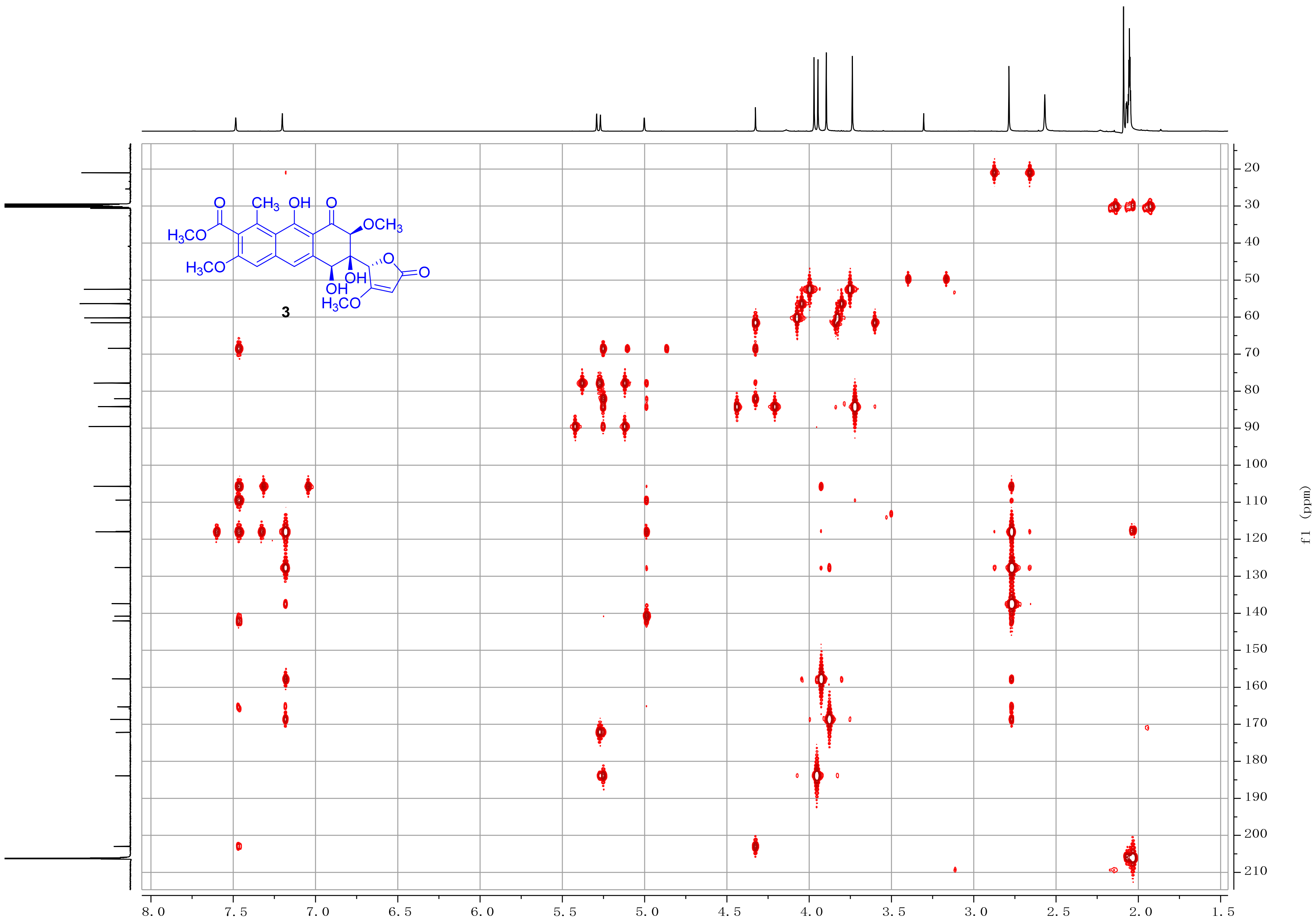

Figure S23. The HMBC spectrum of saccharothrixone G (**3**) in acetone-d_6 (600 MHz).

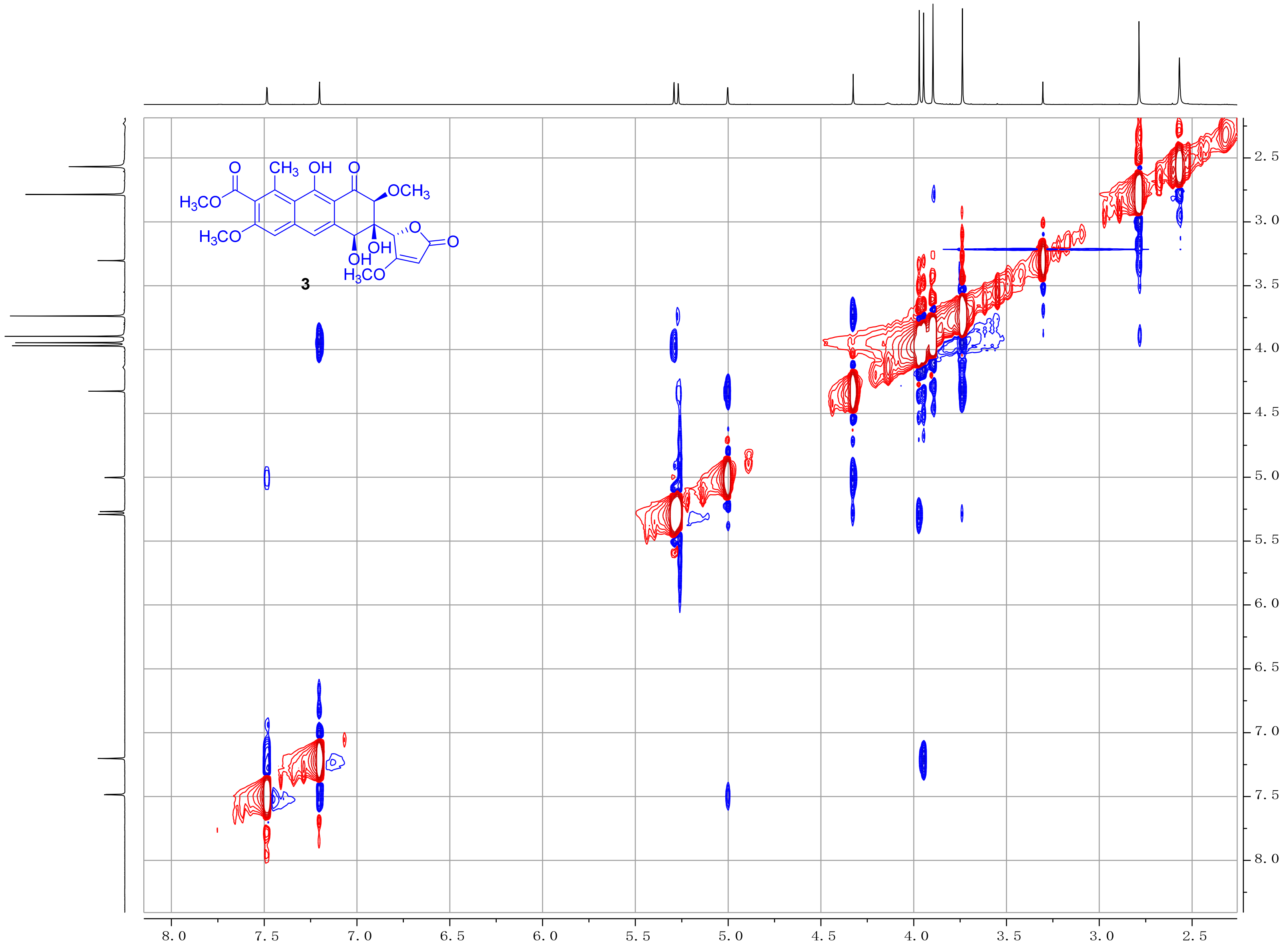

Figure S24. The ROESY spectrum of saccharothrixone G (**3**) in acetone-d_6 (600 MHz).

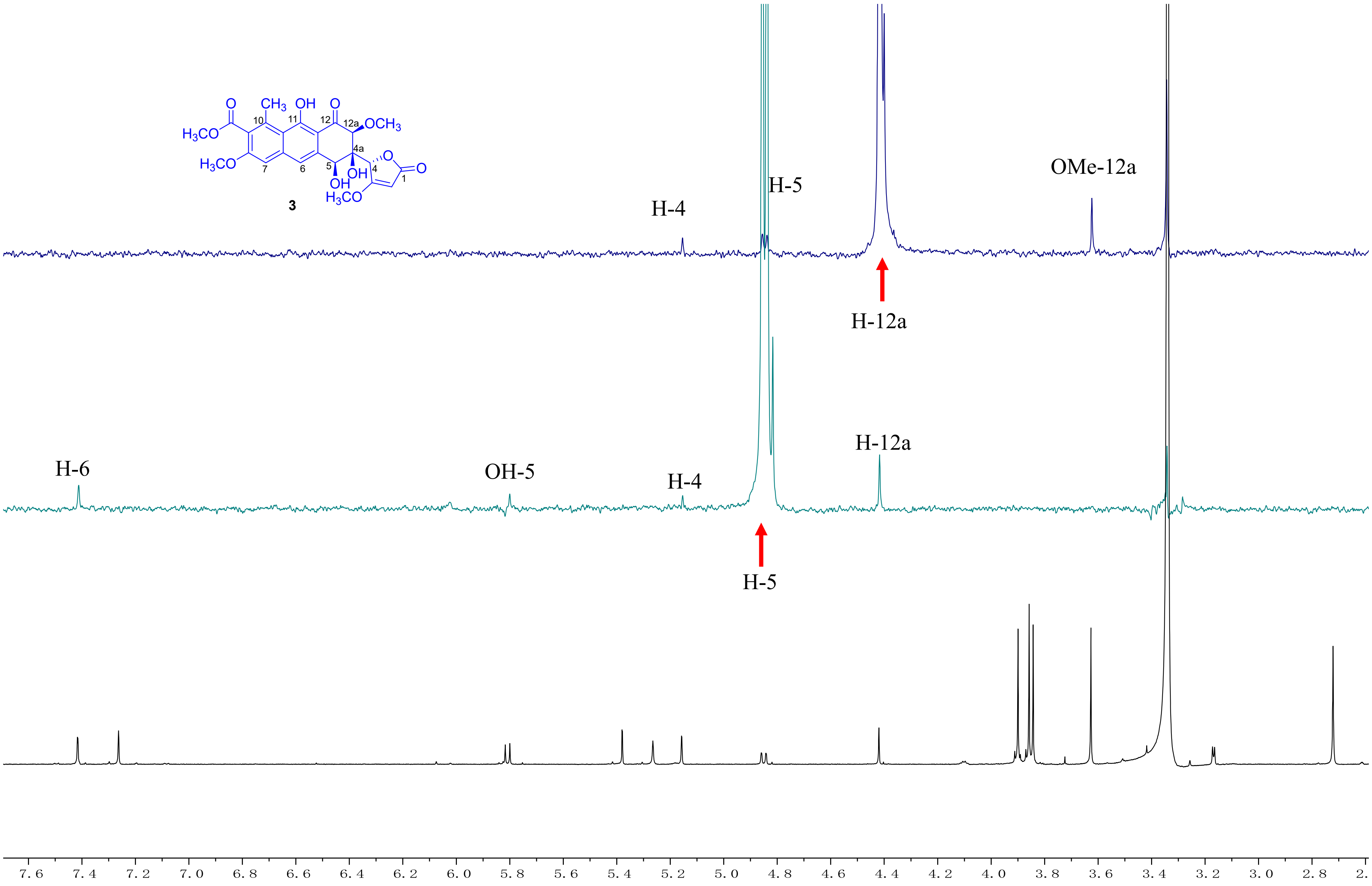

Figure S25. The1D NOE spectrum of saccharothrixone G (**3**) in DMSO-d_6 (600 MHz).

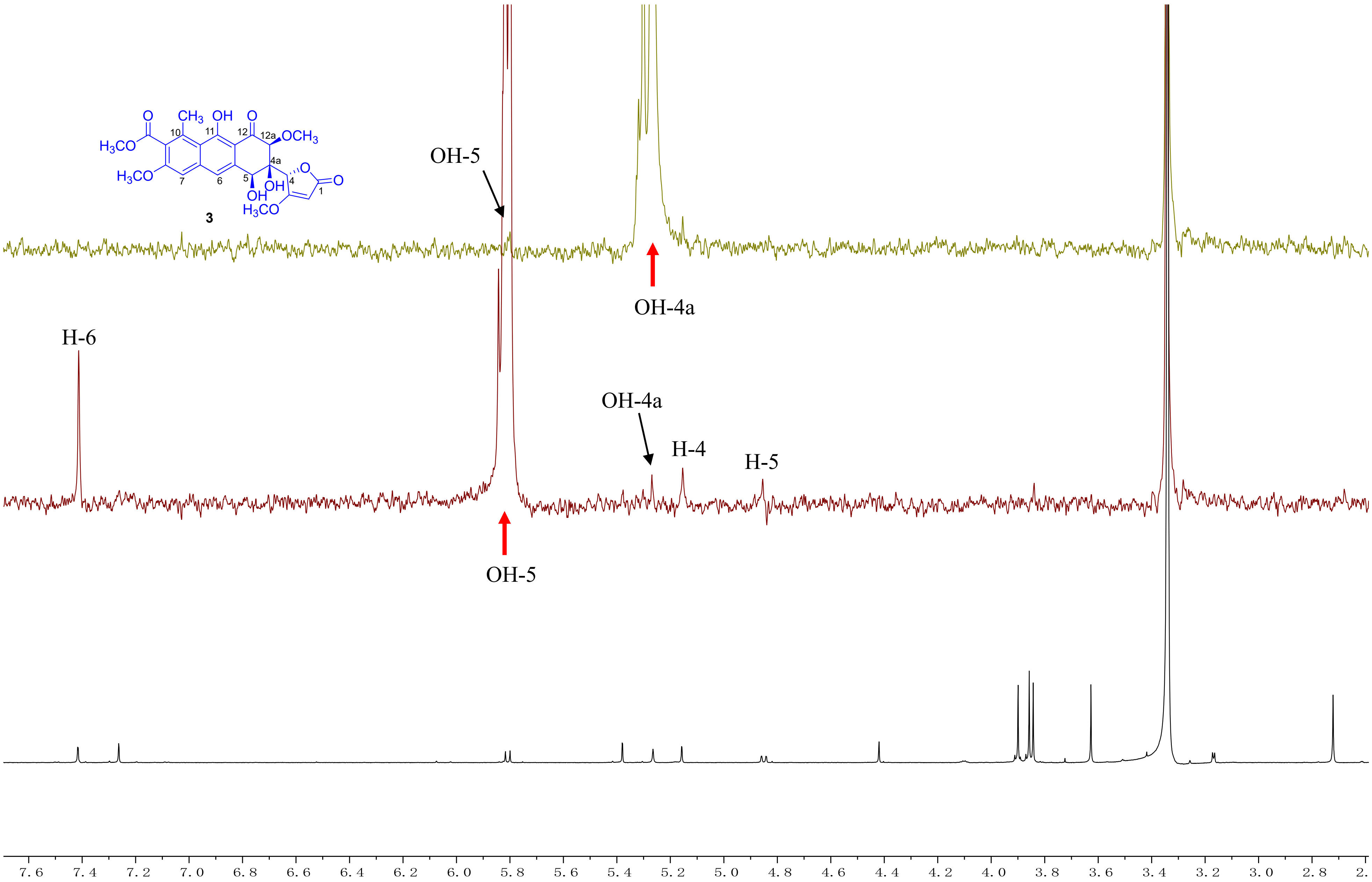

Figure S26. The1D NOE spectrum of saccharothrixone G (**3**) in DMSO-d6 (600 MHz).

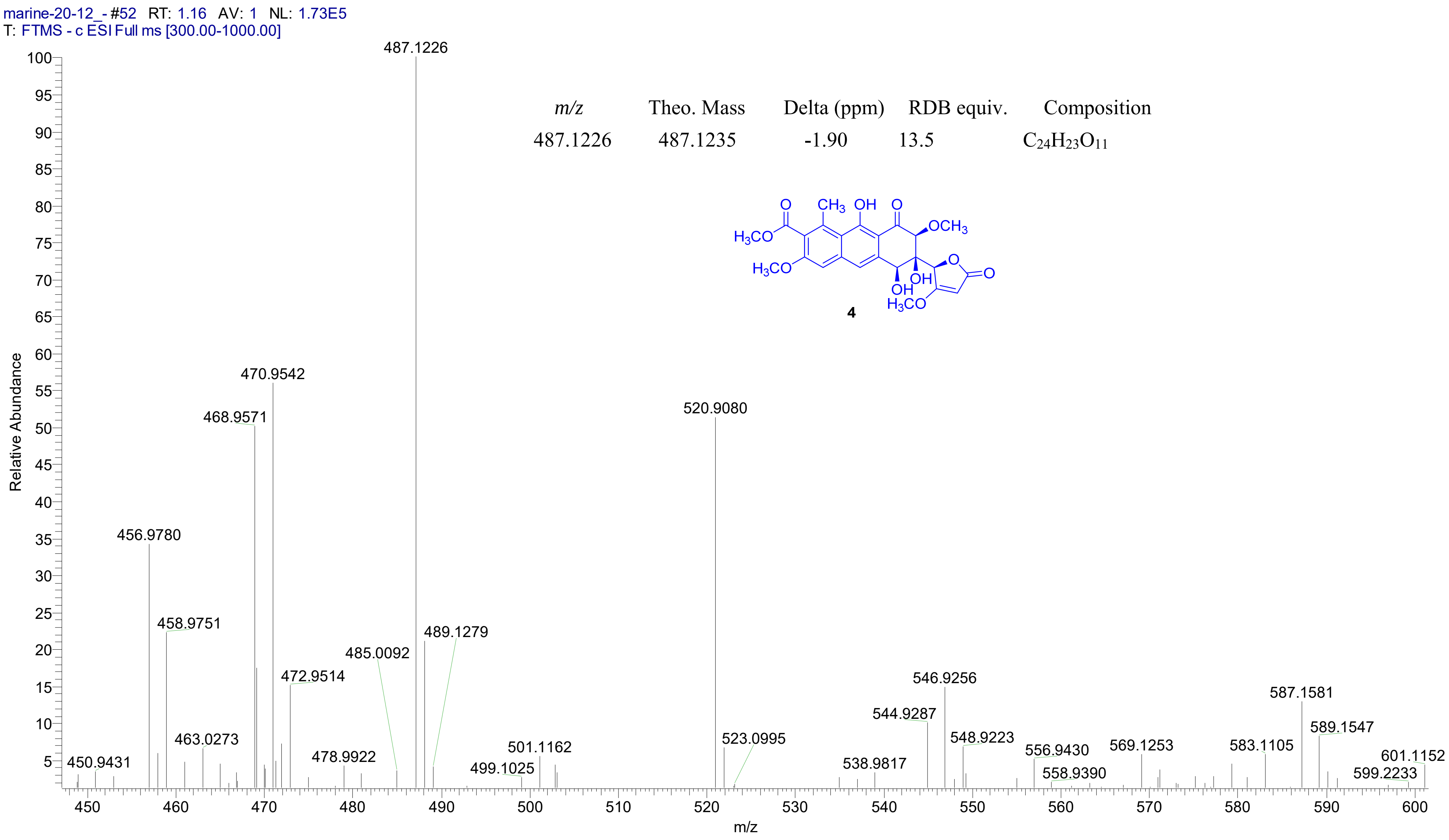

m/z	Theo. Mass	Delta (ppm)	RDB equiv.	Composition
487.1226	487.1235	-1.90	13.5	$C_{24}H_{23}O_{11}$

Figure S27. The (−)-HRESIMS spectrum of saccharothrixone H (**4**).

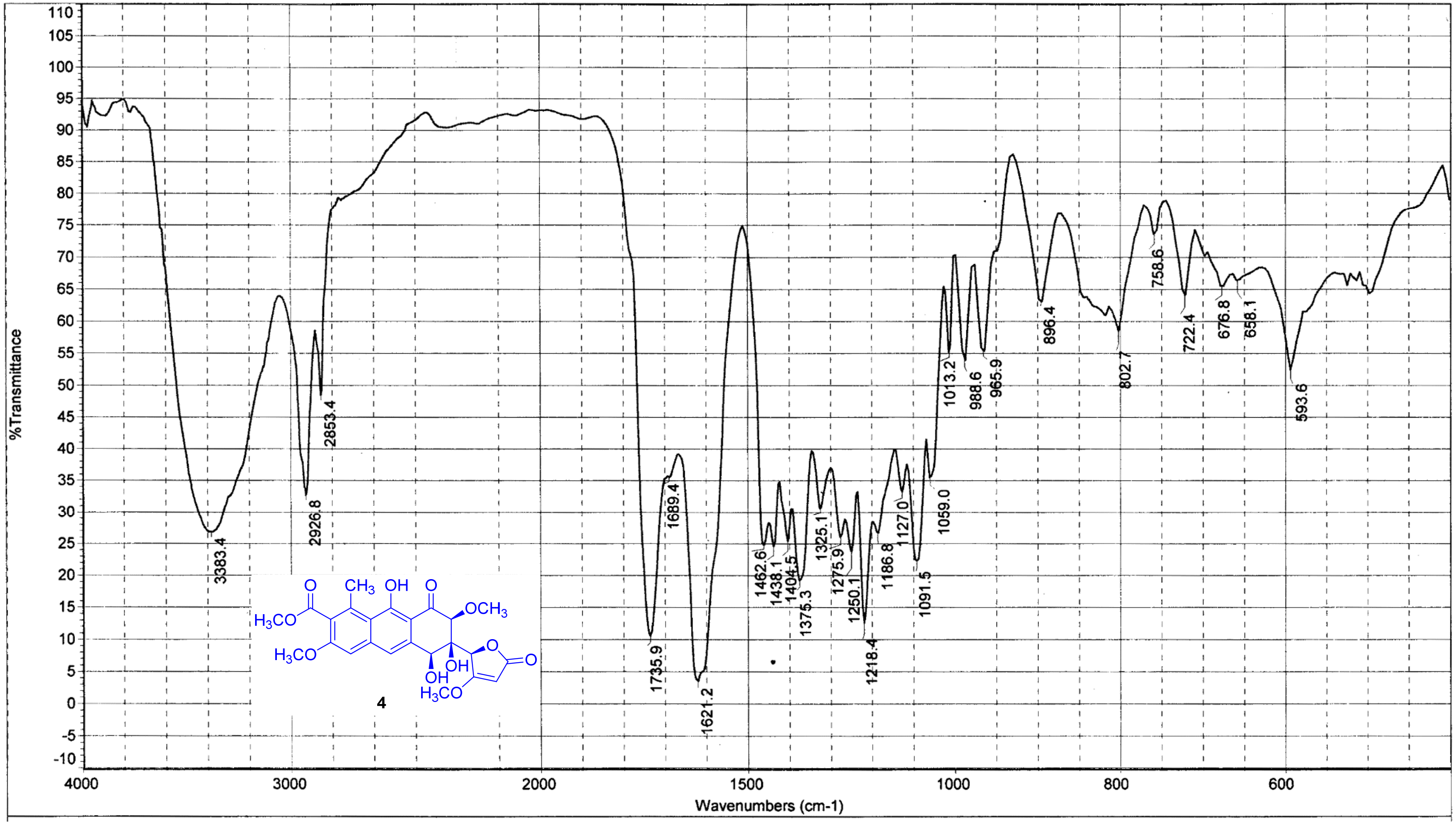

Figure S28. The IR spectrum of saccharothrixone H (**4**).

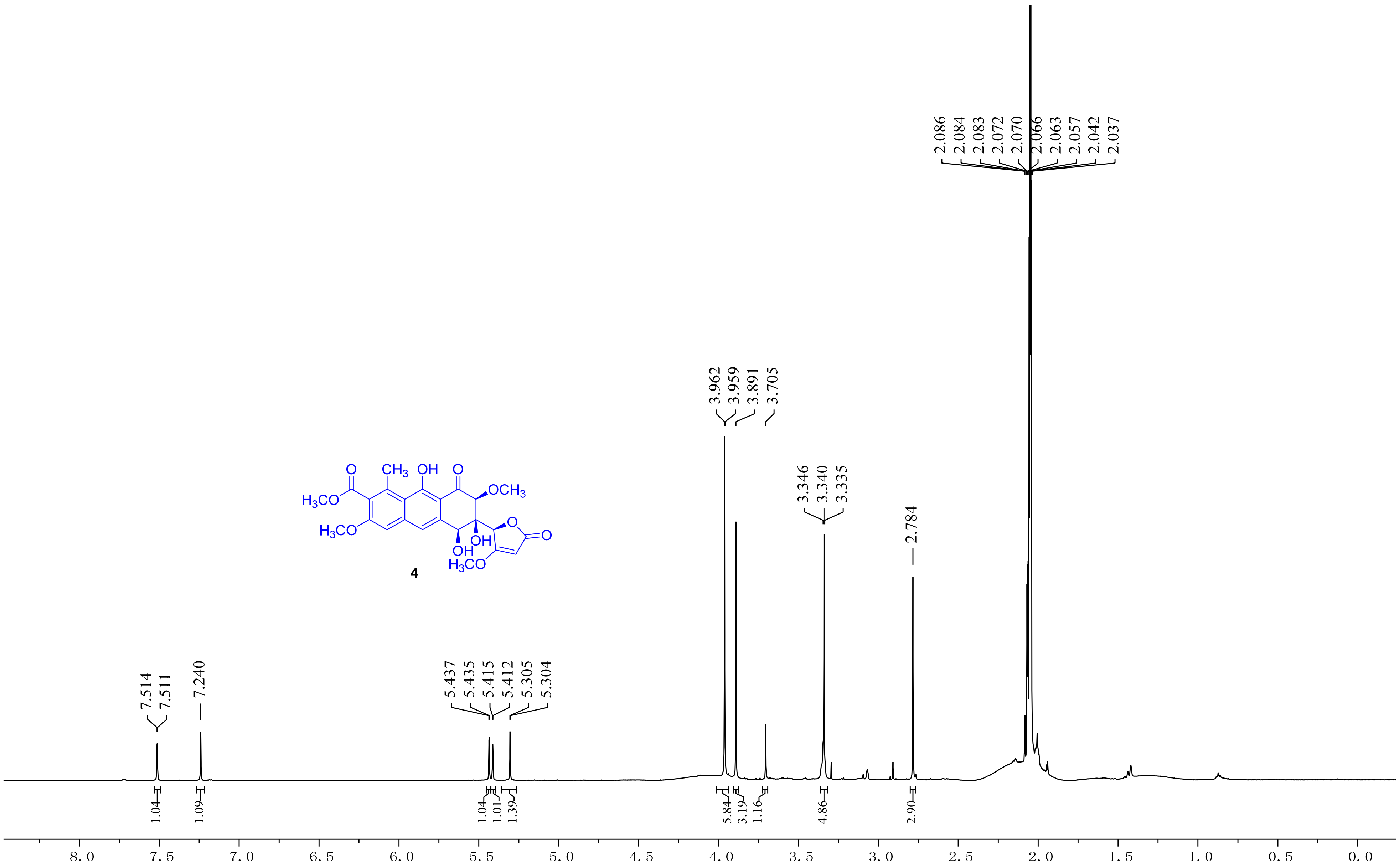

Figure S29. The ^{1}H NMR spectrum of saccharothrixone H (**4**) in acetone-d_6 (600 MHz).

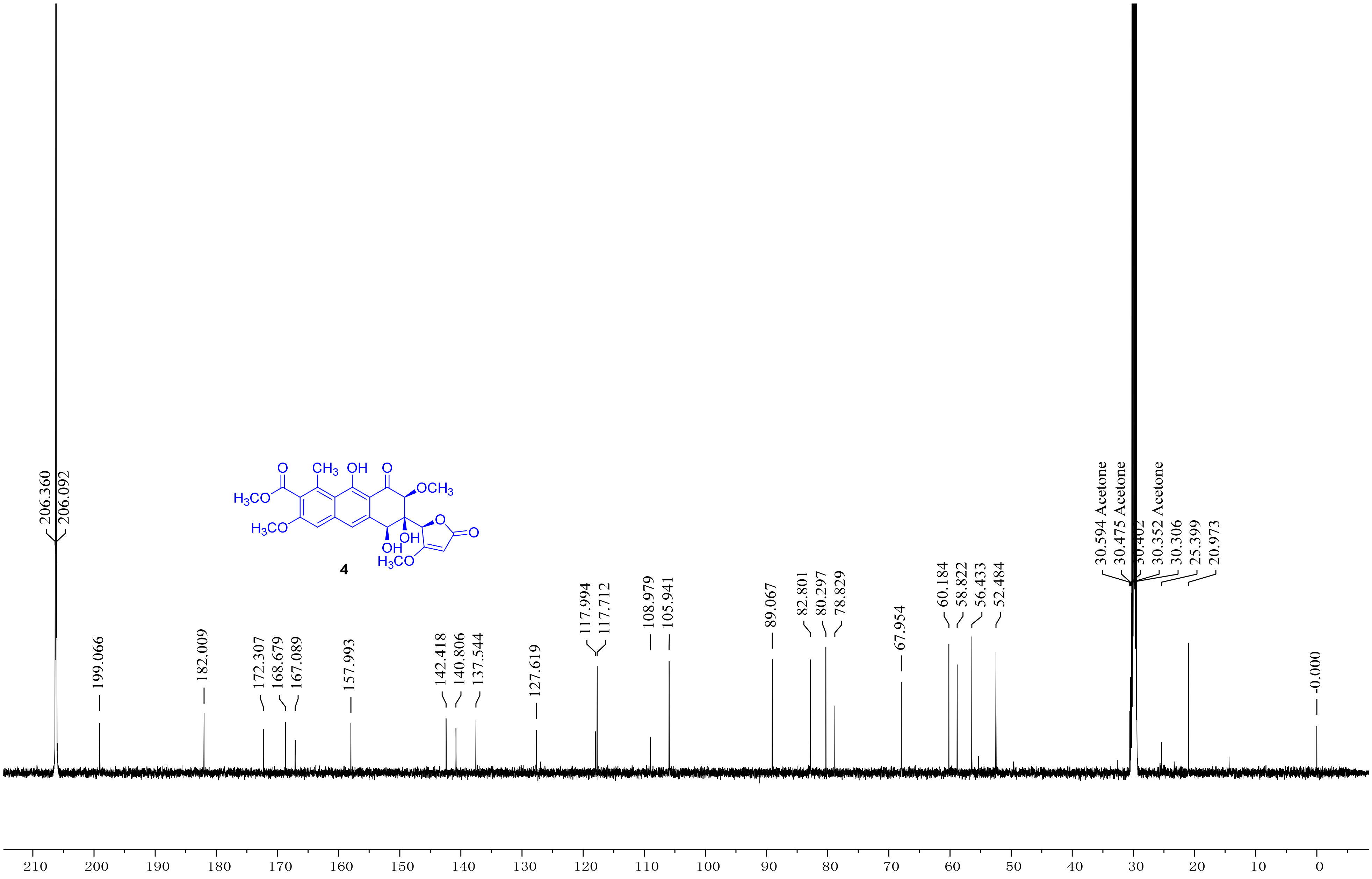

Figure S30. The ^{13}C NMR spectrum of saccharothrixone H (**4**) in acetone-d_6 (150 MHz).

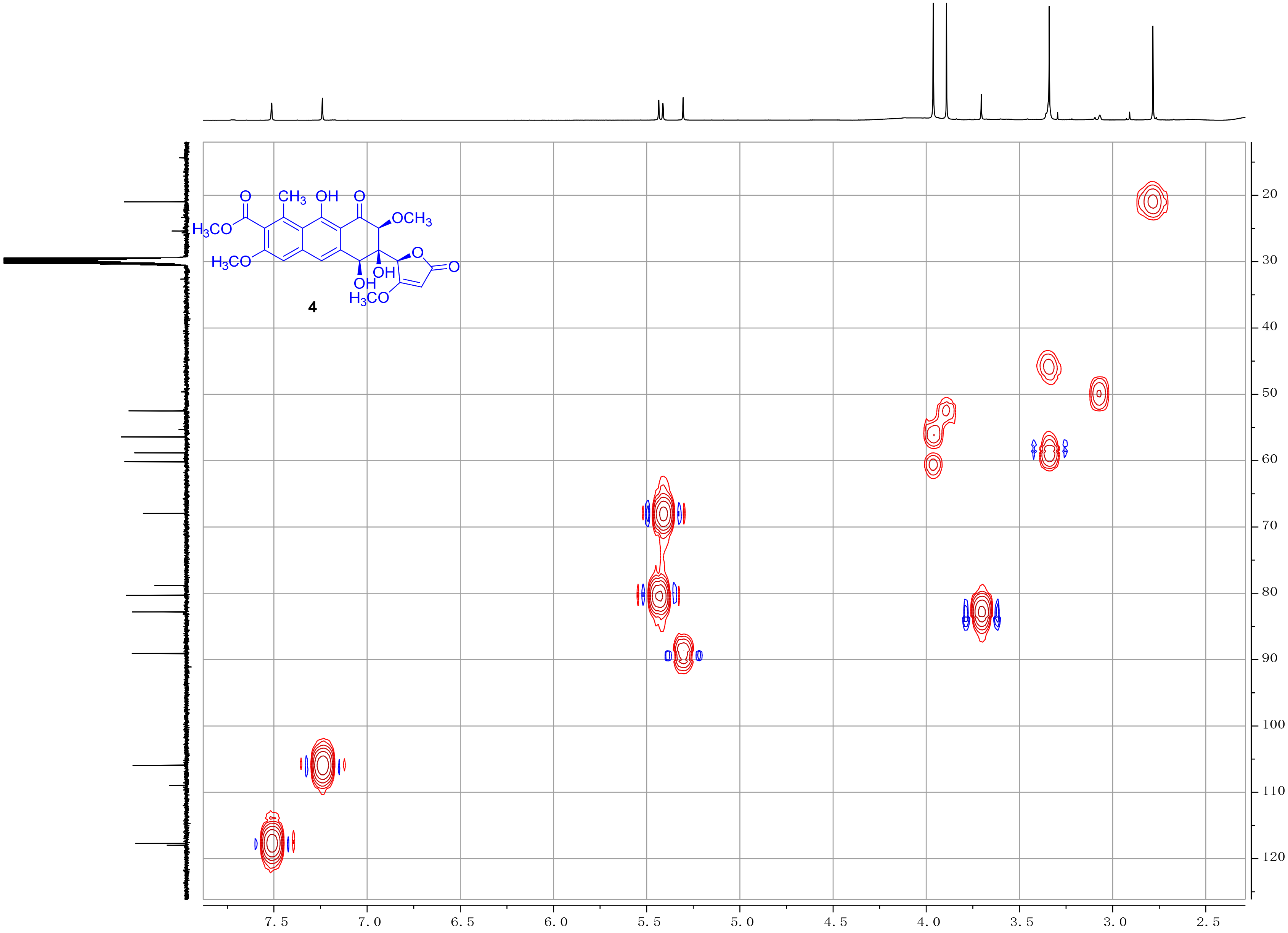

Figure S31. The HSQC spectrum of saccharothrixone H (**4**) in acetone-d_6 (600 MHz).

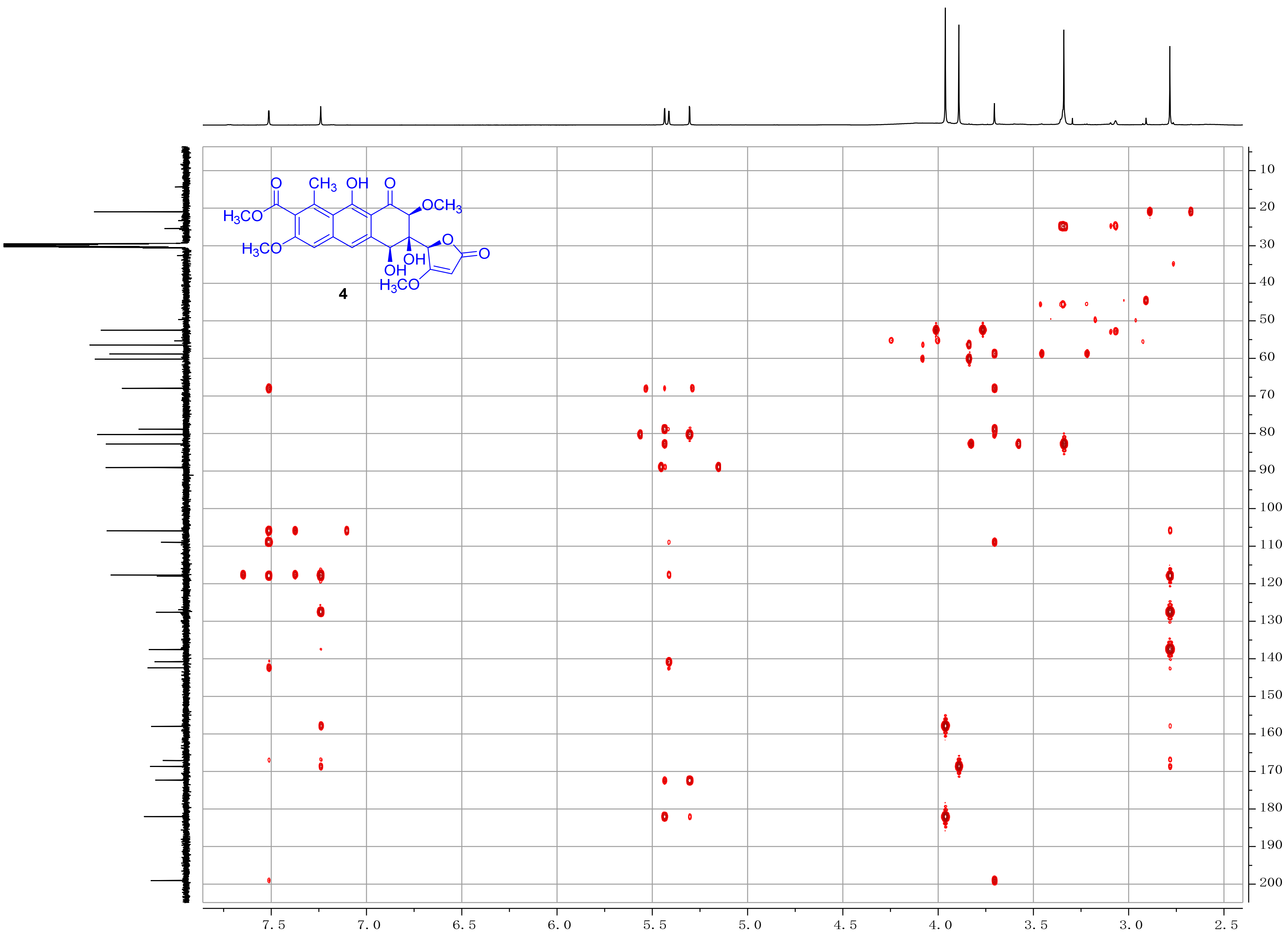

Figure S32. The HMBC spectrum of saccharothrixone H (**4**) in acetone-d_6 (600 MHz).

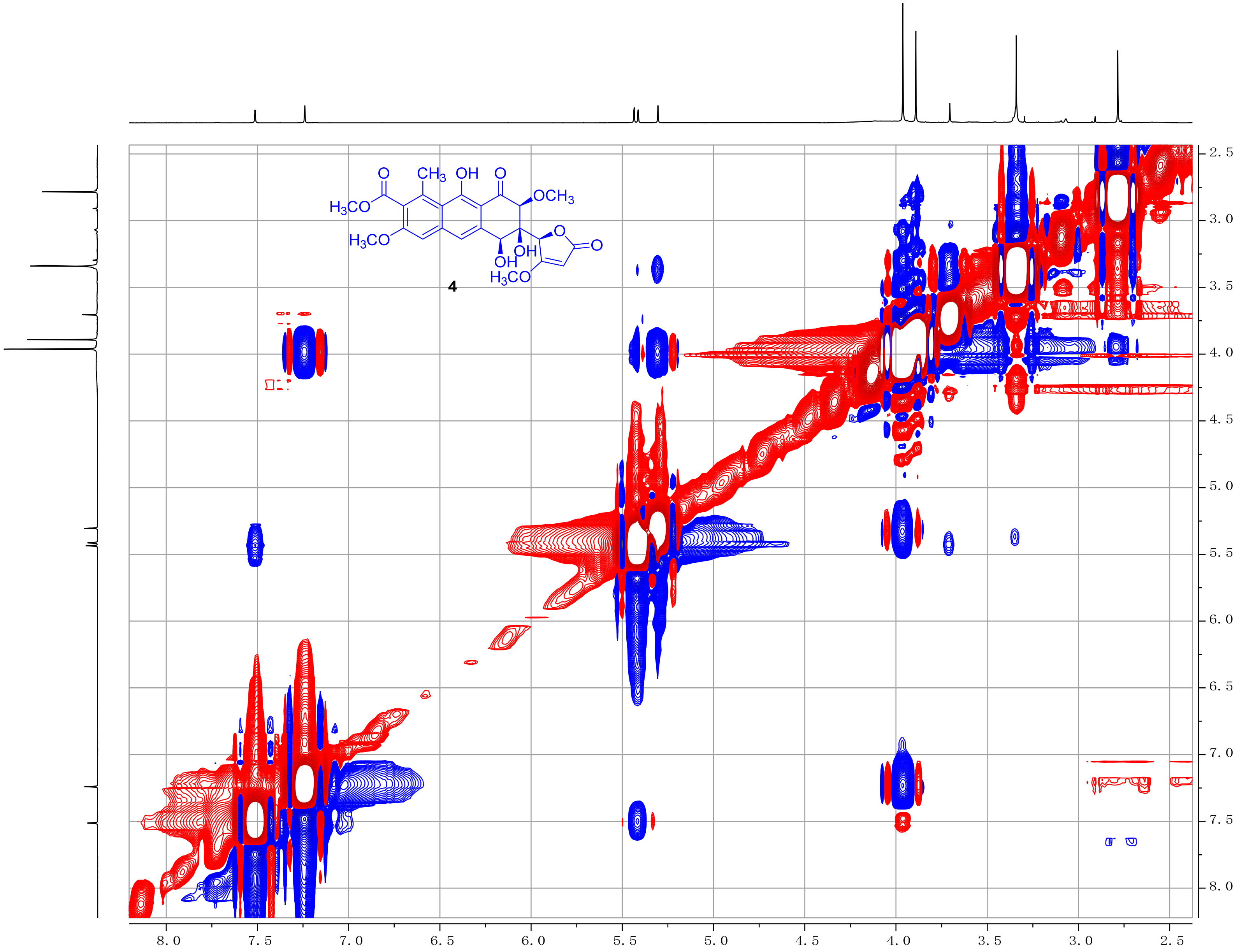

Figure S33. The ROESY spectrum of saccharothrixone H (**4**) in acetone-d_6 (600 MHz).

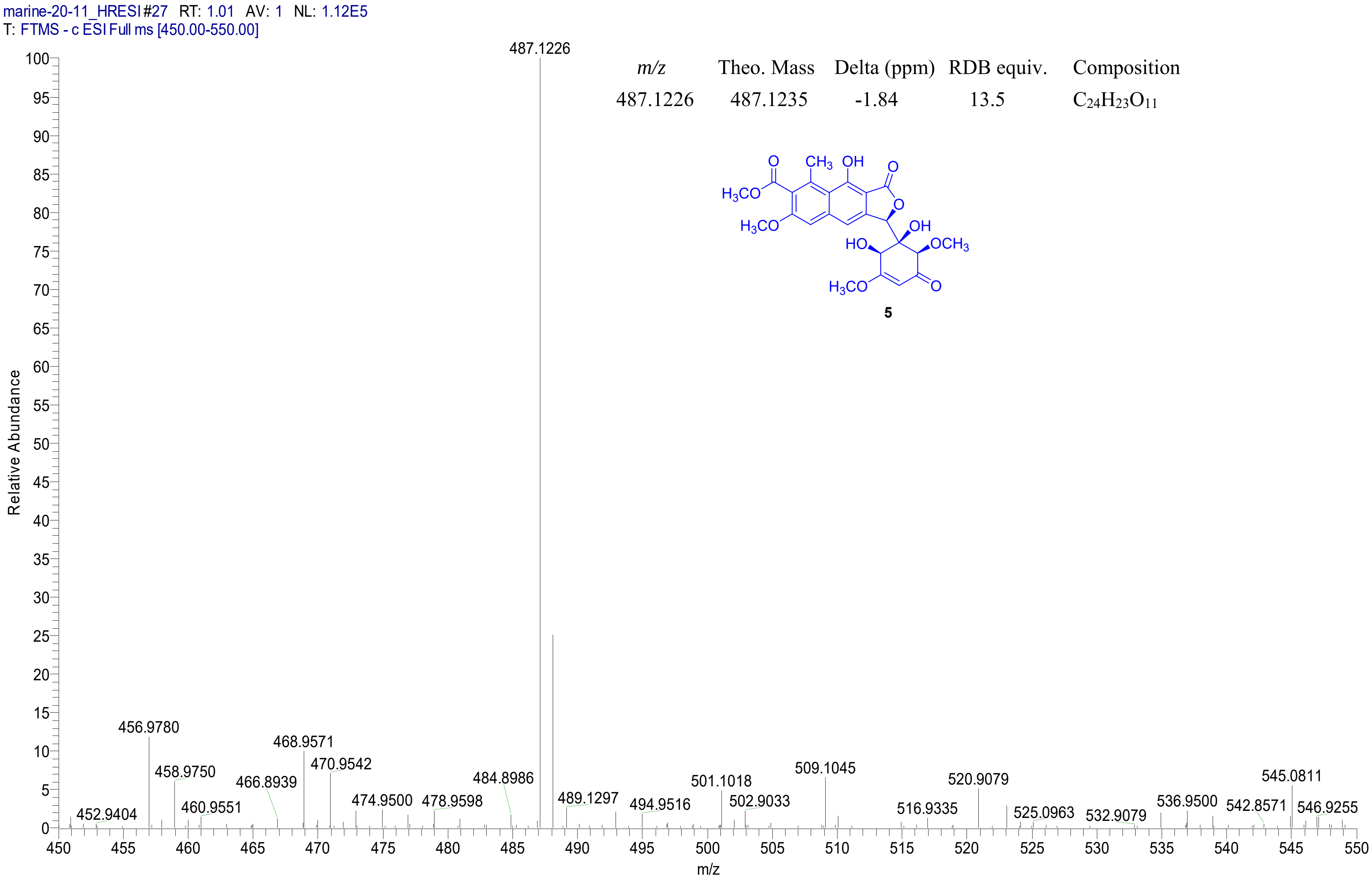

m/z	Theo. Mass	Delta (ppm)	RDB equiv.	Composition
487.1226	487.1235	-1.84	13.5	$C_{24}H_{23}O_{11}$

Figure S34. The (−)-HRESIMS spectrum of saccharothrixone I (**5**).

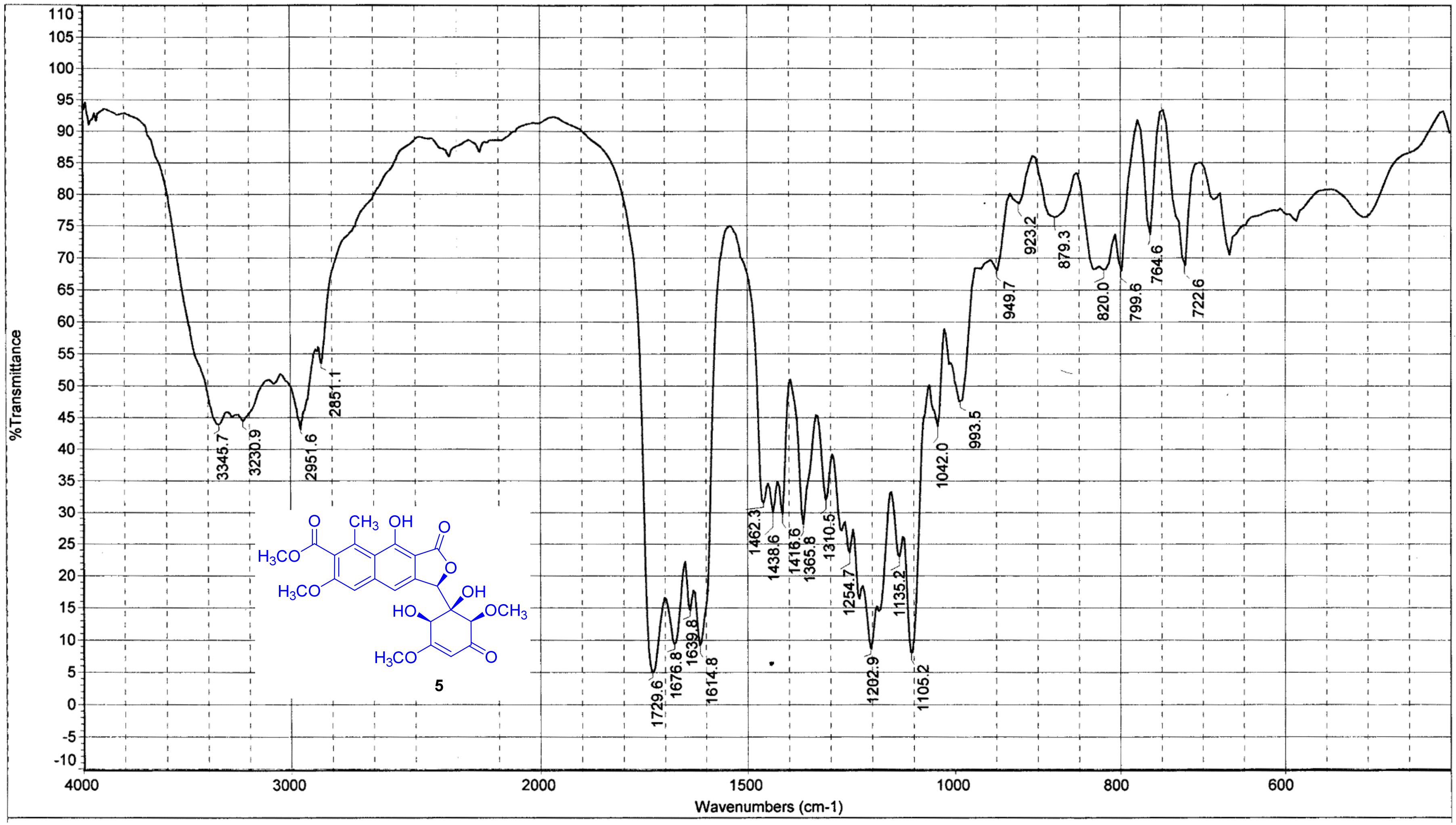

Figure S35. The IR spectrum of saccharothrixone I (**5**).

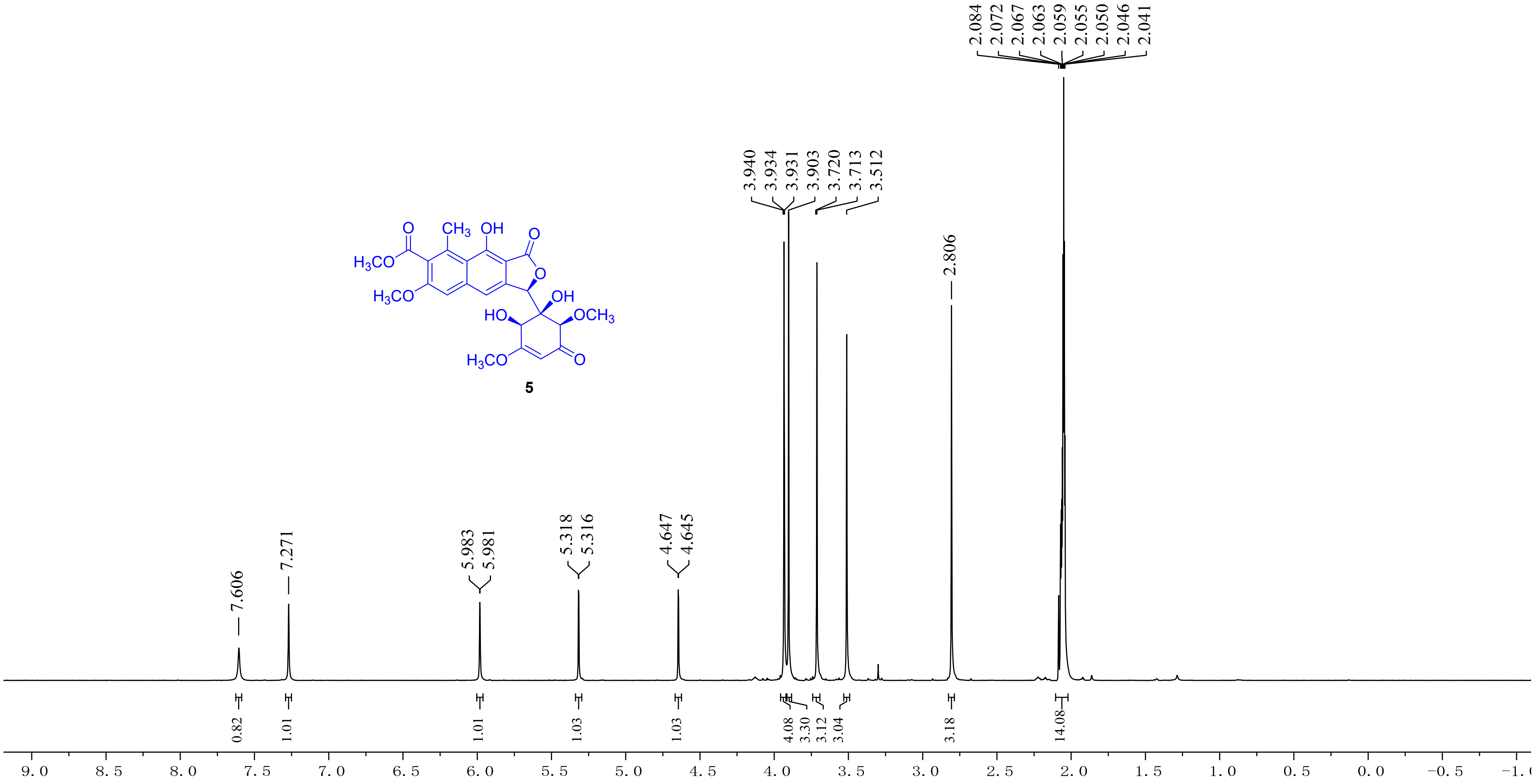

Figure S36. The ^{1}H NMR spectrum of saccharothrixone I (**5**) in acetone-d_6 (500 MHz).

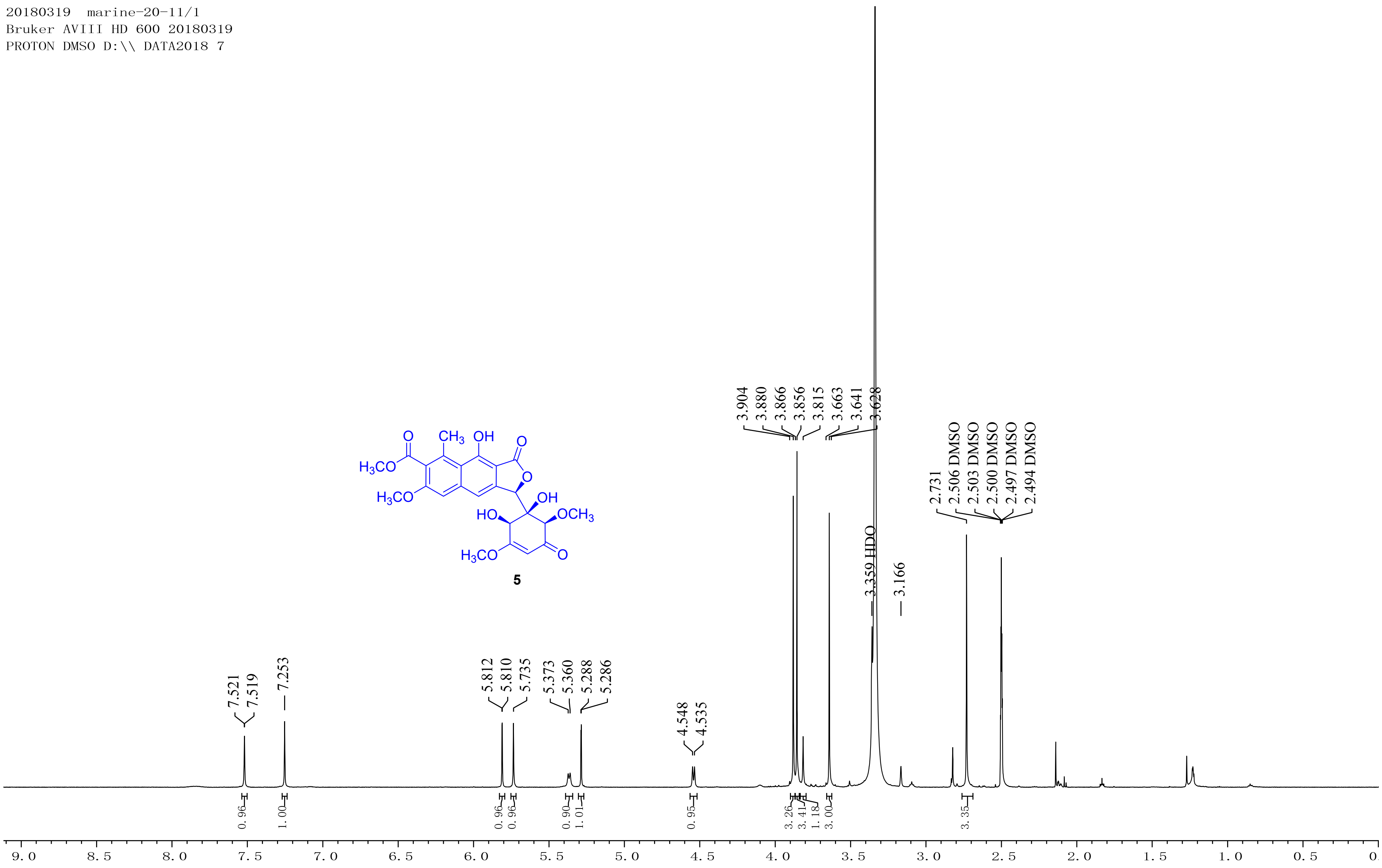

Figure S37. The 1H NMR spectrum of saccharothrixone I (**5**) in DMSO-d6 (600 MHz).

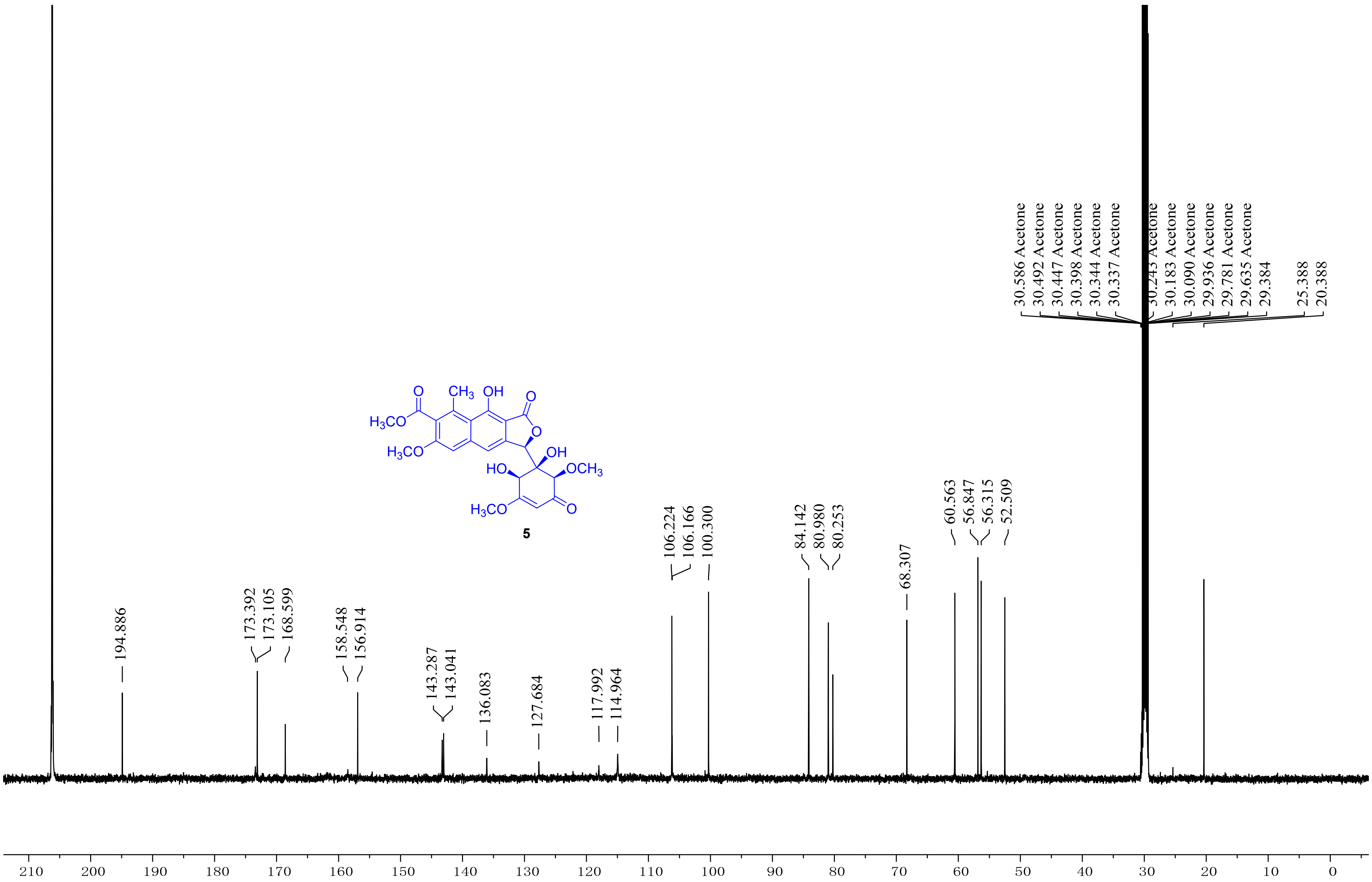

Figure S38. The ^{13}C NMR spectrum of saccharothrixone I (**5**) in acetone-d_6 (125 MHz).

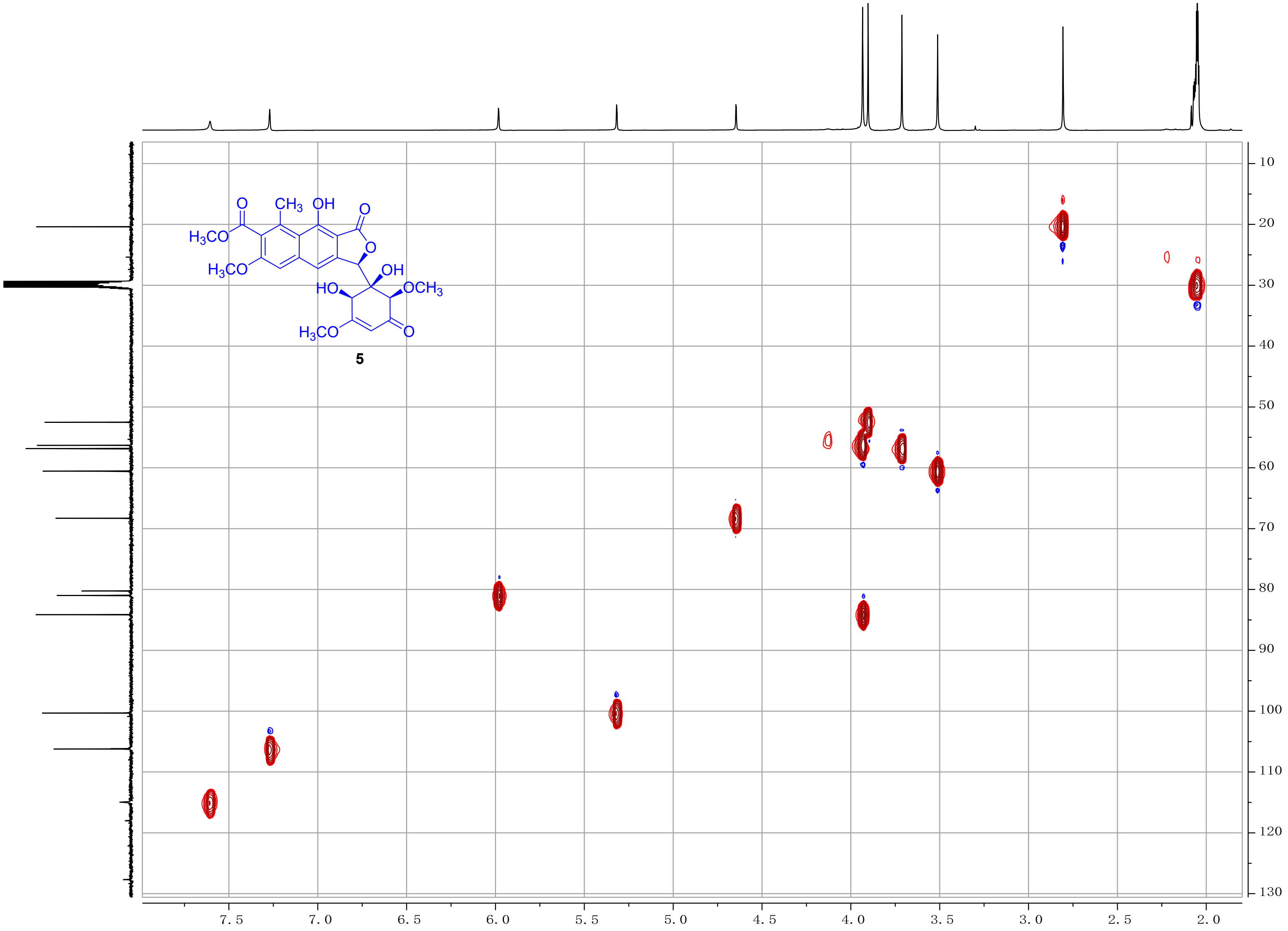

Figure S39. The HSQC spectrum of saccharothrixone I (**5**) in acetone-d_6 (500 MHz).

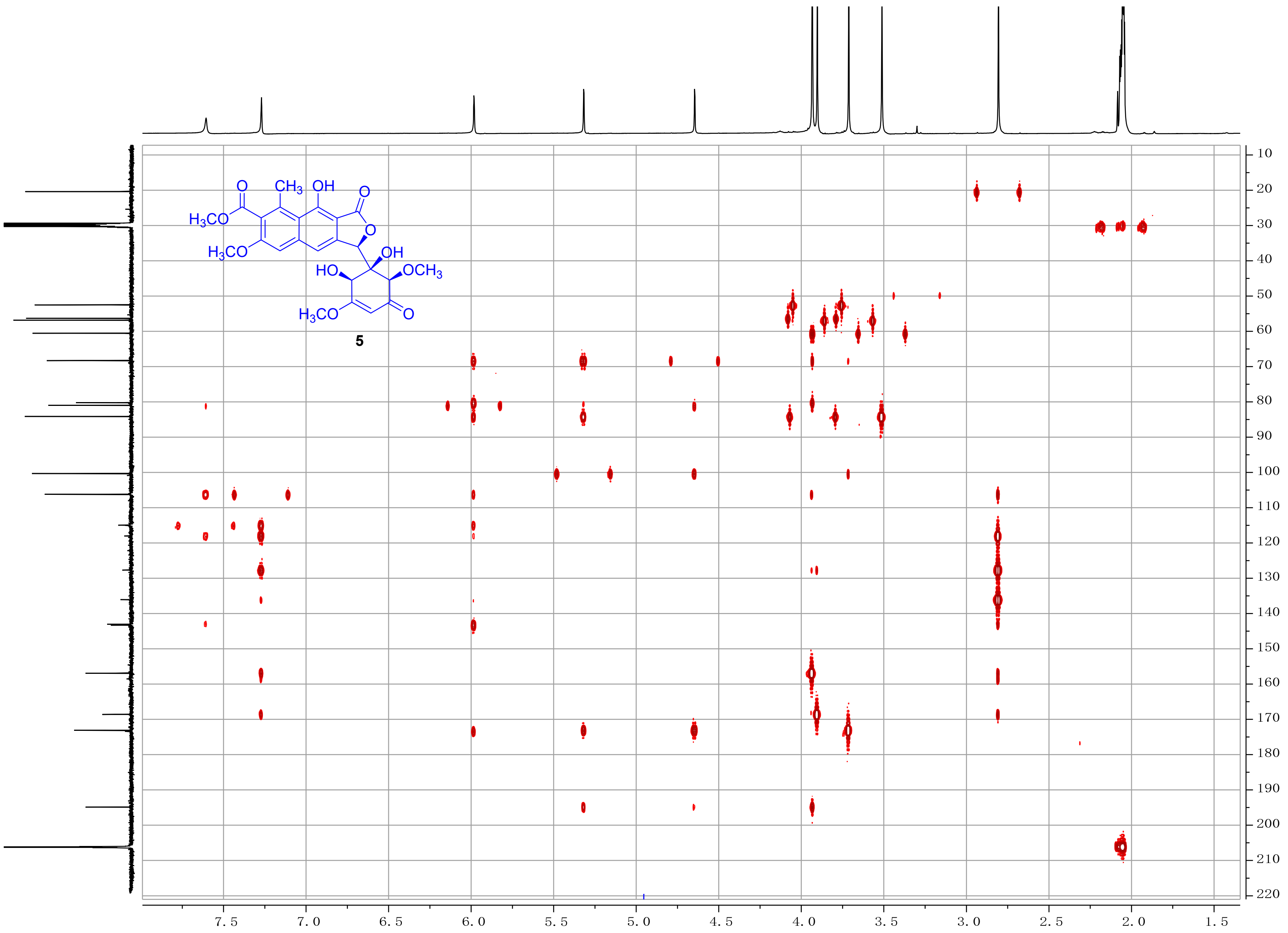

Figure S40. The HMBC spectrum of saccharothrixone I (**5**) inacetone-d_6 (500 MHz).

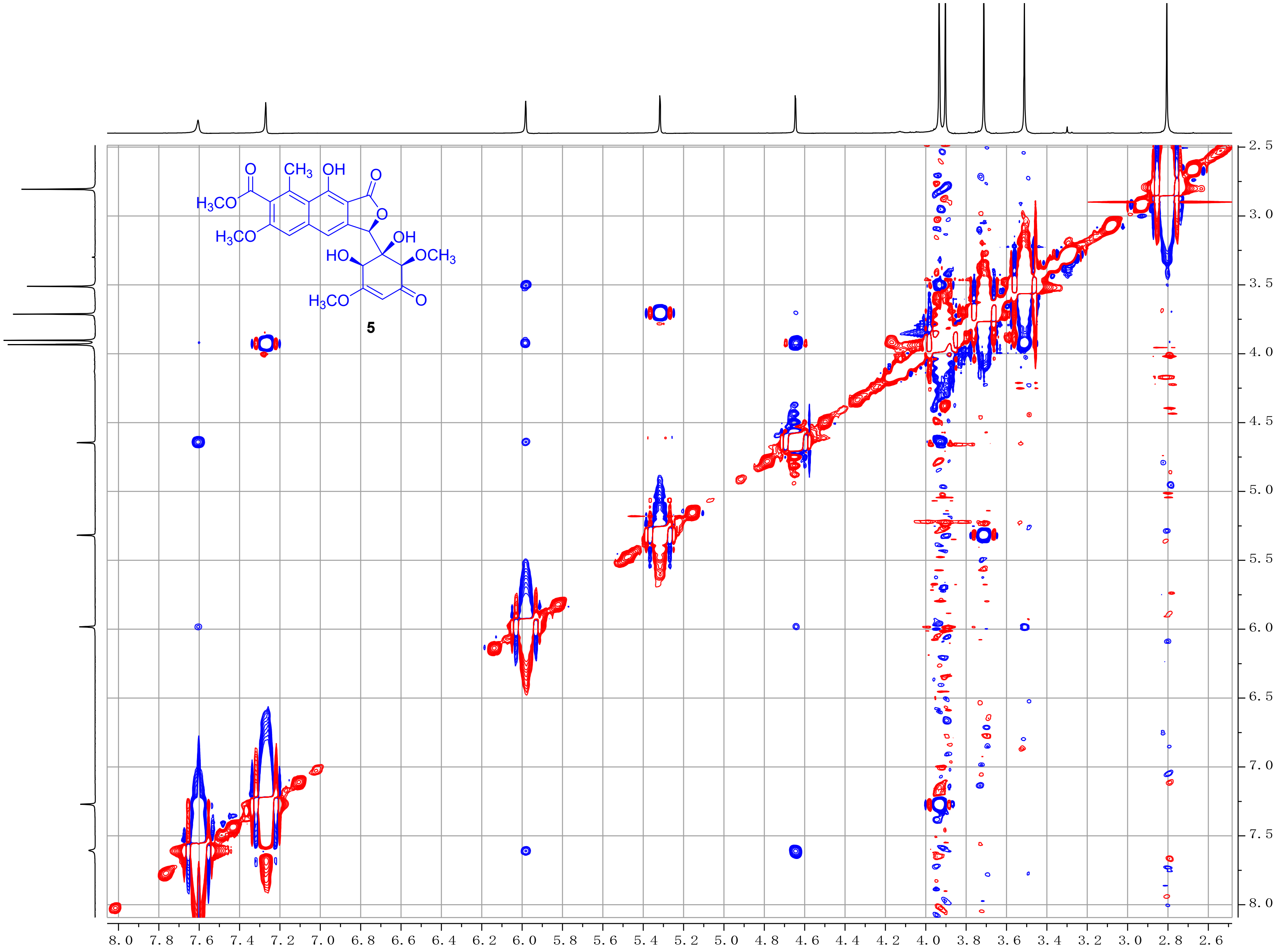

Figure S41. The ROESY spectrum of saccharothrixone I (**5**) in acetone-d_6 (600 MHz).

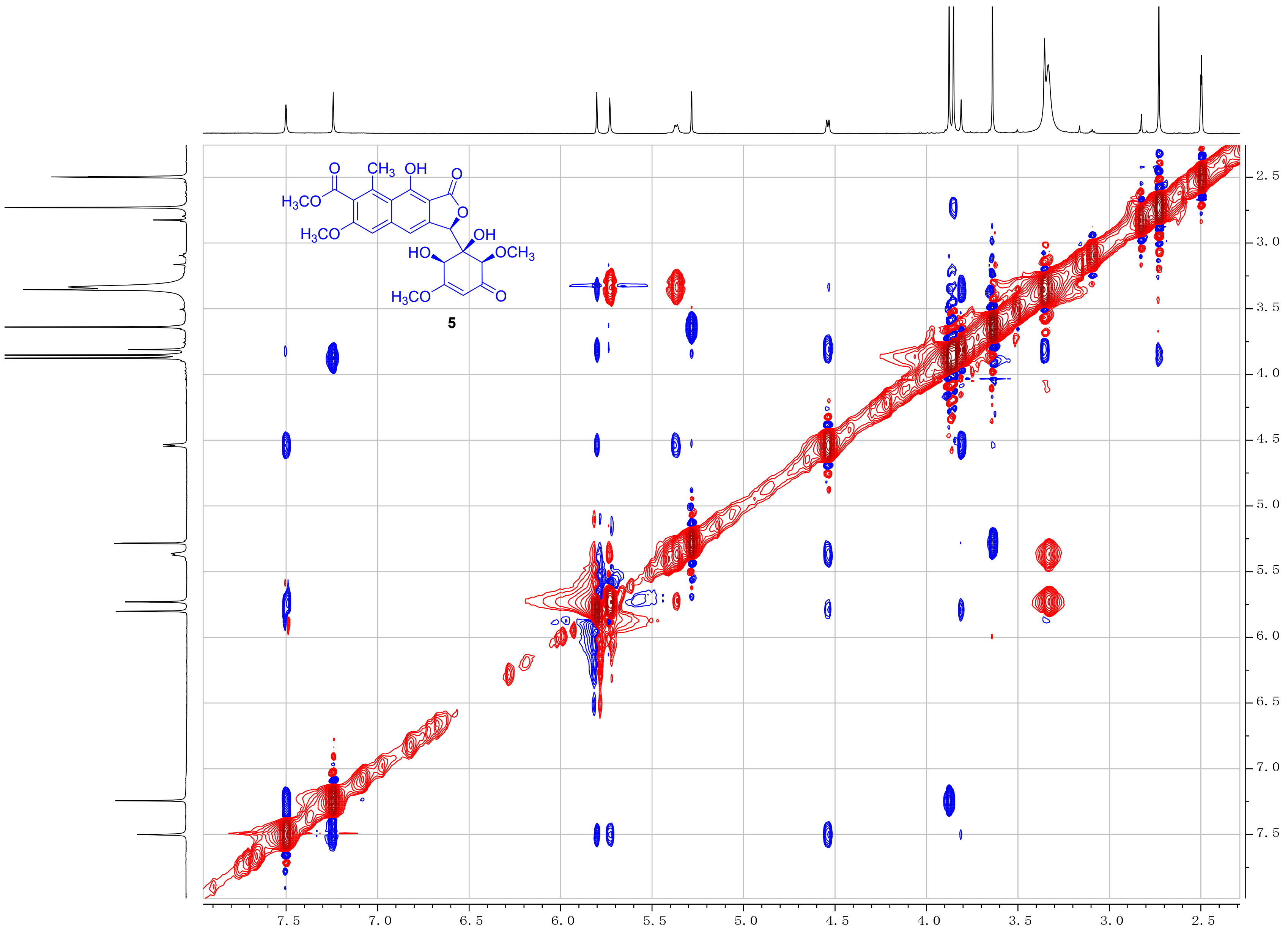

Figure S42. The ROESY spectrum of saccharothrixone I (**5**) in DMSO-d_6 (600 MHz).

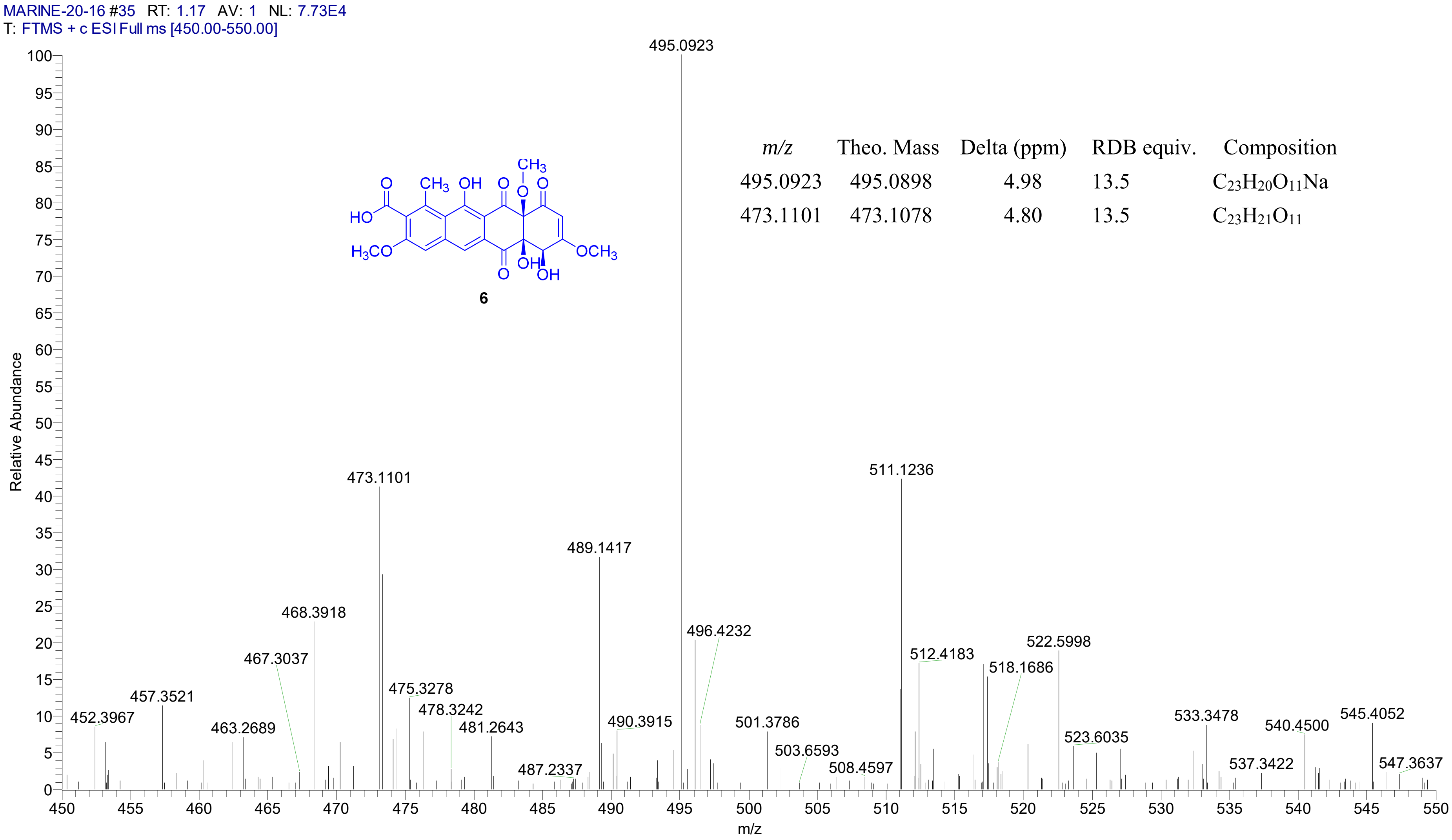

m/z	Theo. Mass	Delta (ppm)	RDB equiv.	Composition
495.0923	495.0898	4.98	13.5	$C_{23}H_{20}O_{11}Na$
473.1101	473.1078	4.80	13.5	$C_{23}H_{21}O_{11}$

Figure S43. The (+)-HR ESIMS spectrum of 13-de-*O*-methyltetracenomycin X (**6**).

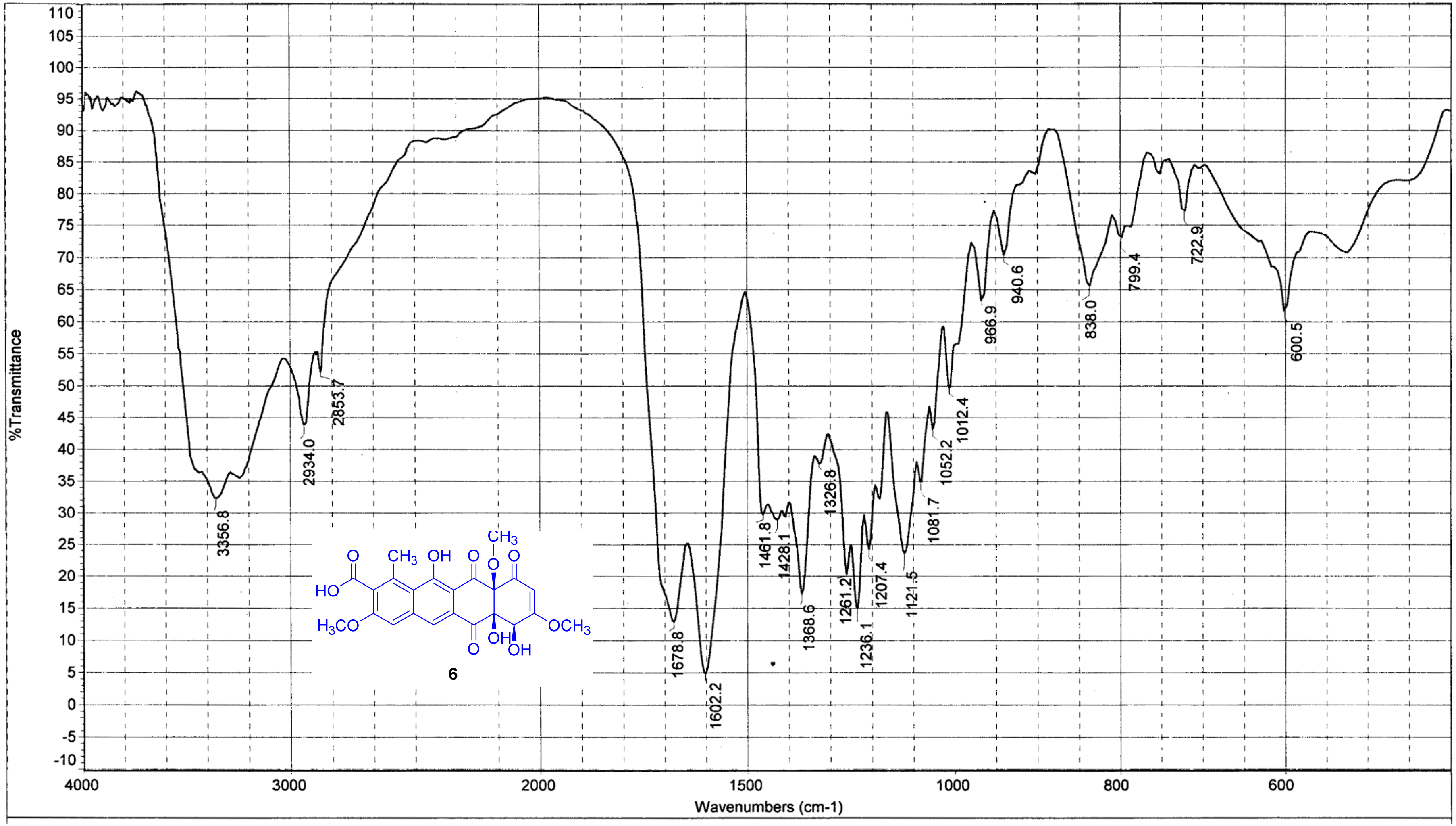

Figure S44. The IR spectrum of 13-de-*O*-methyltetracenomycin X (**6**).

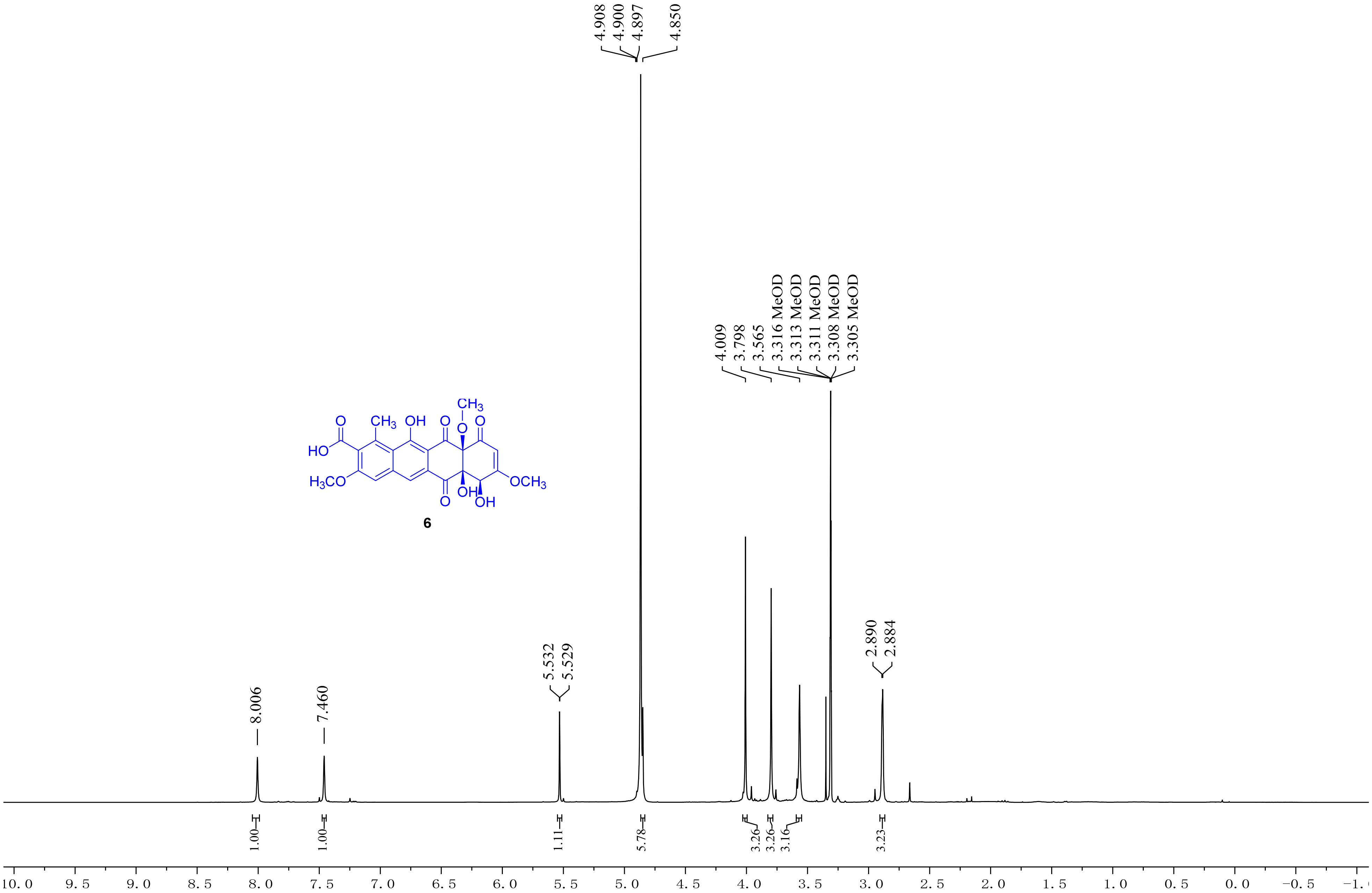

Figure S45. The ^{1}H NMR spectrum of 13-de-*O*-methyltetracenomycin X (**6**) in CD_3OD (600 MHz).

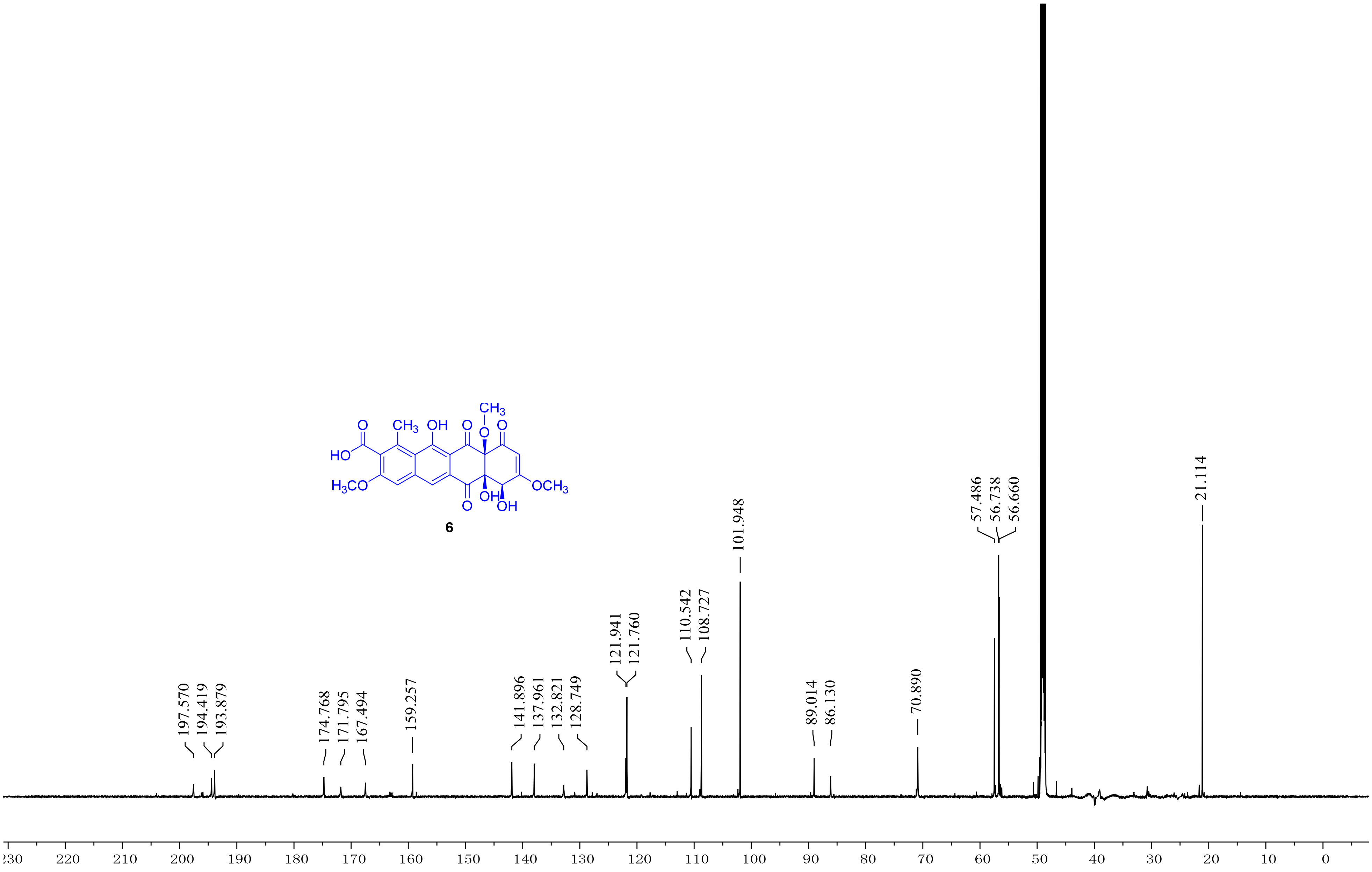

Figure S46. The ^{13}C NMR spectrum of 13-de-*O*-methyltetracenomycin X (**6**) in CD_3OD (150 MHz).

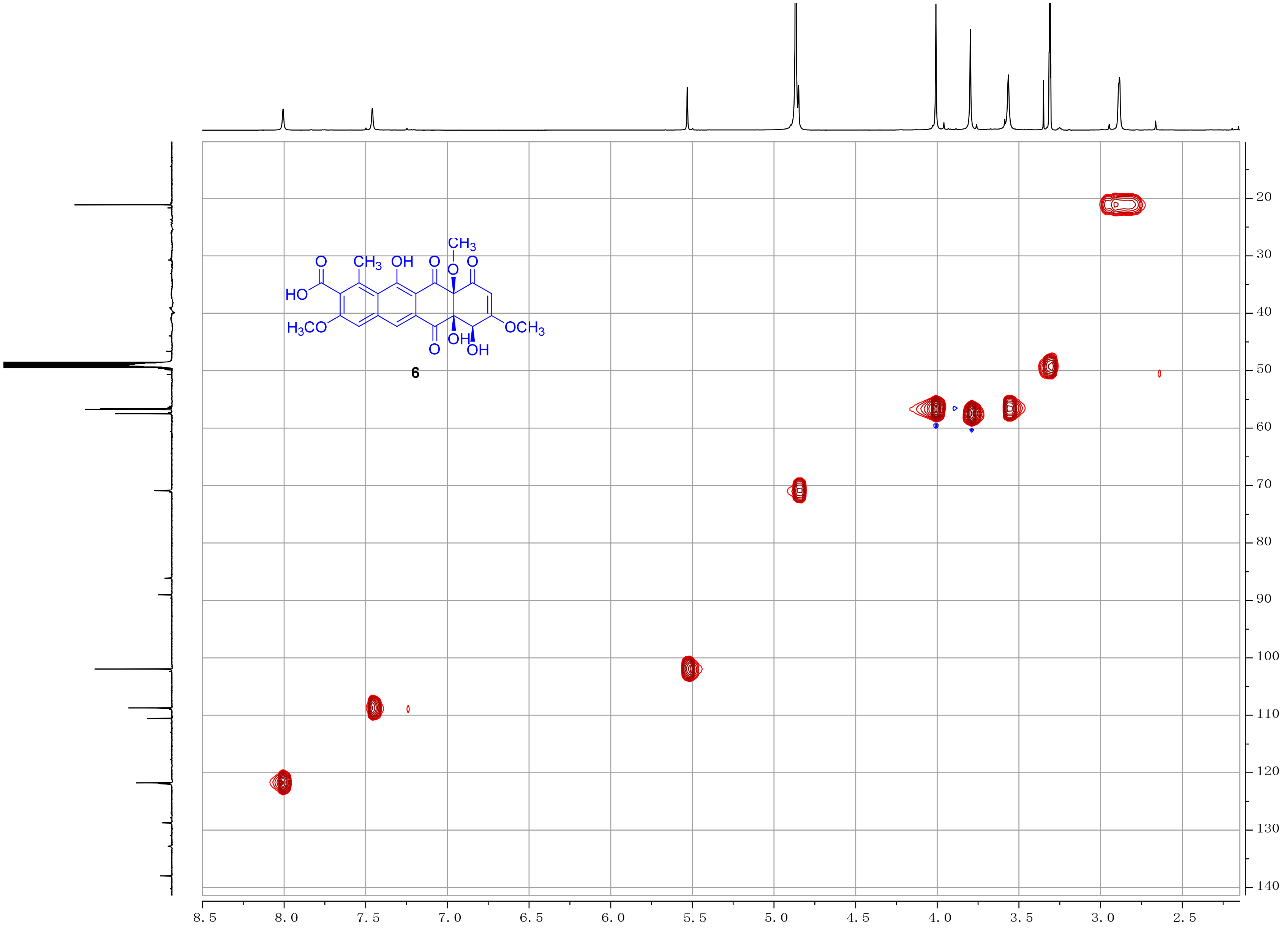

Figure S47. The HSQC spectrum of 13-de-*O*-methyltetracenomycin X (**6**) in CD_3OD (600 MHz).

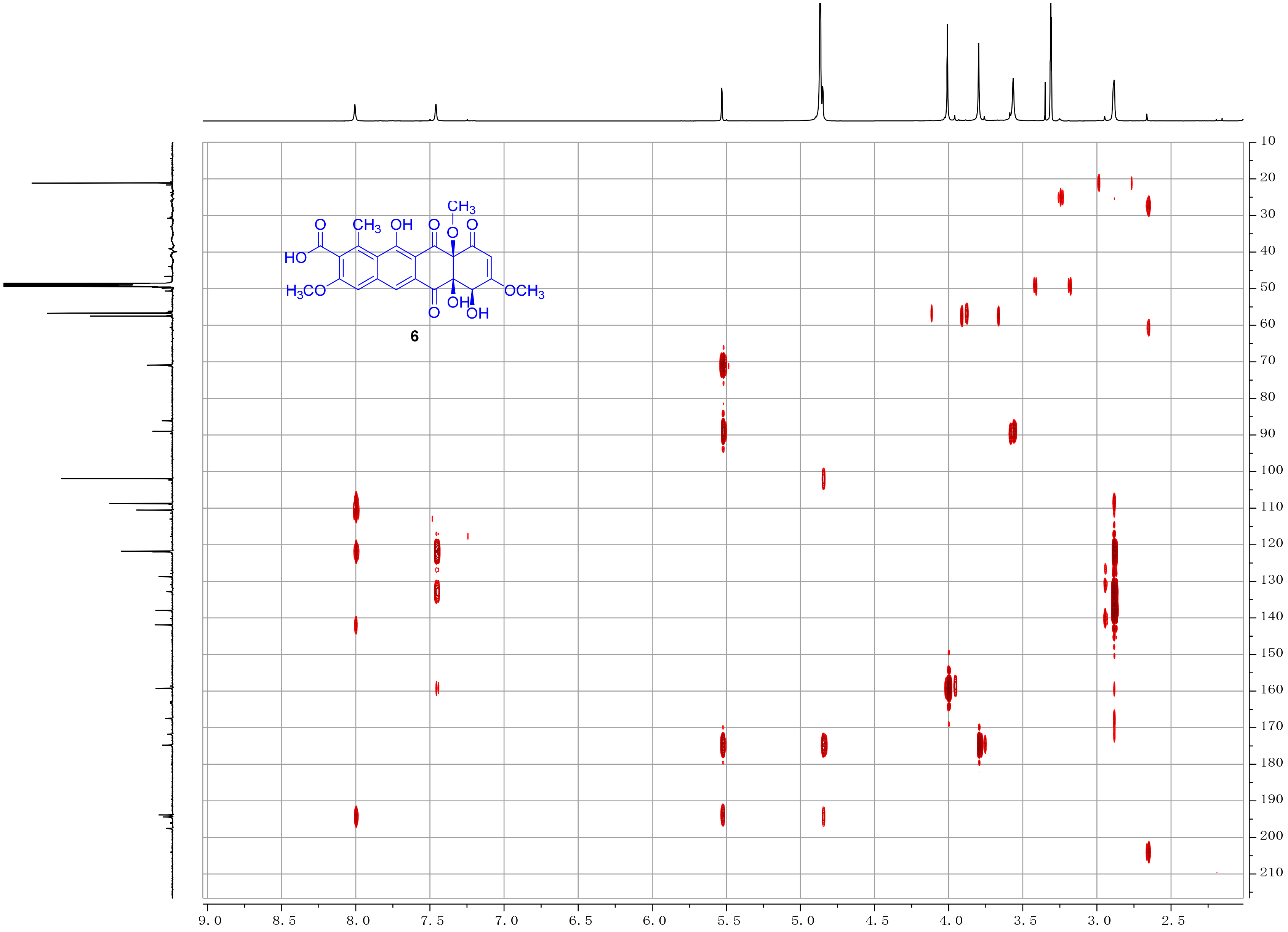

Figure S48. The HMBC spectrum of 13-de-*O*-methyltetracenomycin X (**6**) in CD_3OD (600 MHz).

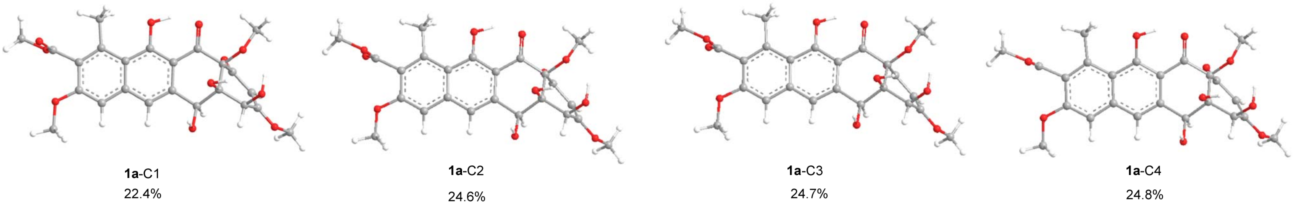

Figure S49. Optimized conformers (≥ 1%) of (4*S*,4a*R*,5*S*,12a*R*)-**1a** at the B3LYP/6-311+G(d,p) level with PCM model in MeOH.

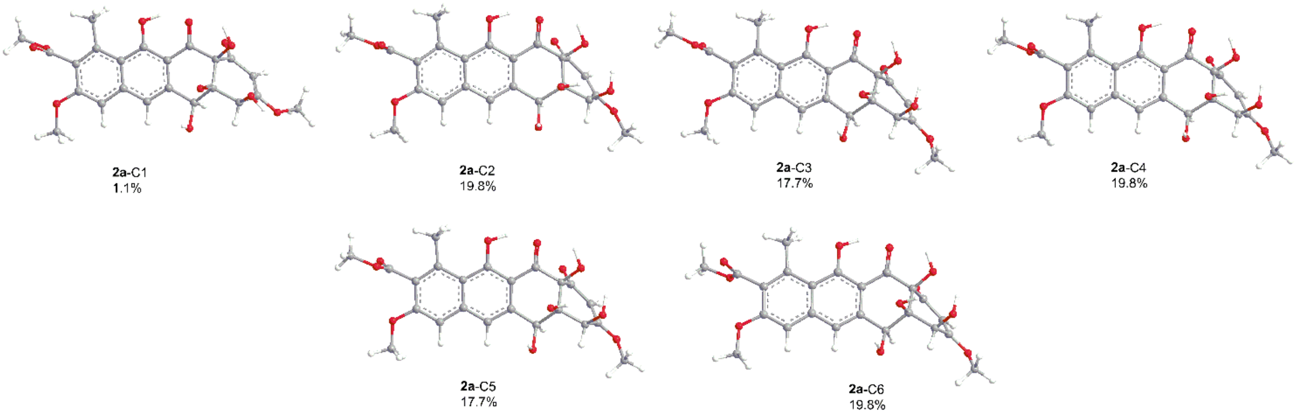

Figure S50. Optimized conformers (≥ 1%) of (4*S*,4a*R*,5*S*,12a*R*)-**2a** at the B3LYP/6-311+G(d,p) level with PCM model in MeOH.

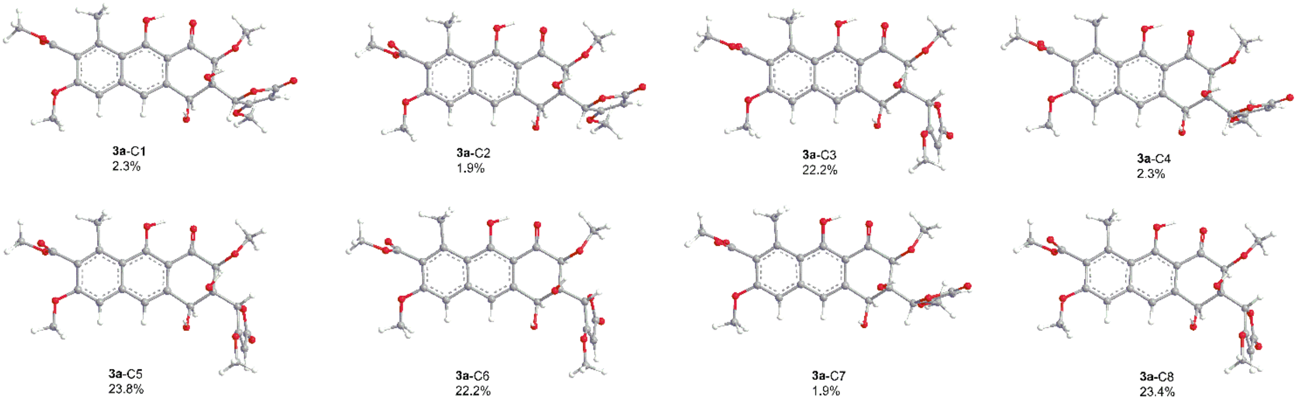

Figure S51. Optimized conformers (≥ 1%) of (4*S*,4a*R*,5*S*,12a*S*)-**3a** at the B3LYP/6-311+G(d,p) level with PCM model in MeOH.

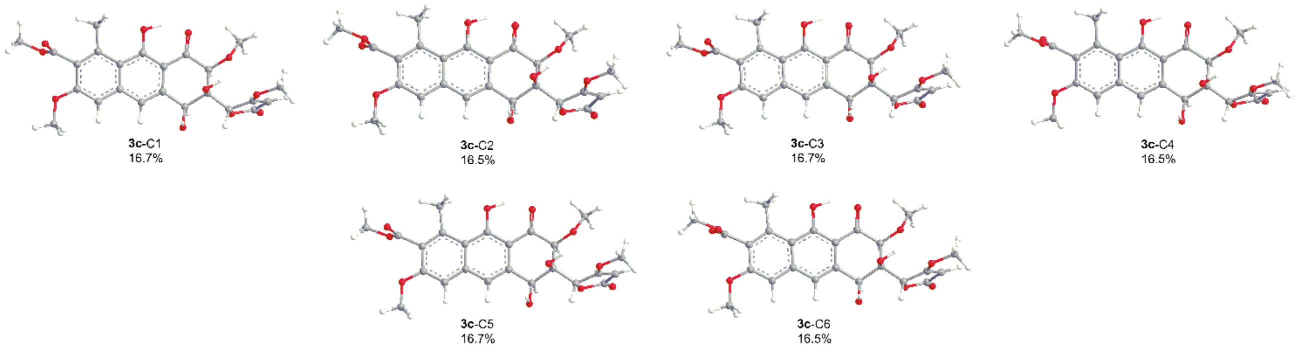

Figure S52. Optimized conformers (≥ 1%) of (4*R*,4a*R*,5*S*,12a*S*)-**3c** at the B3LYP/6-311+G(d,p) level with PCM model in MeOH.

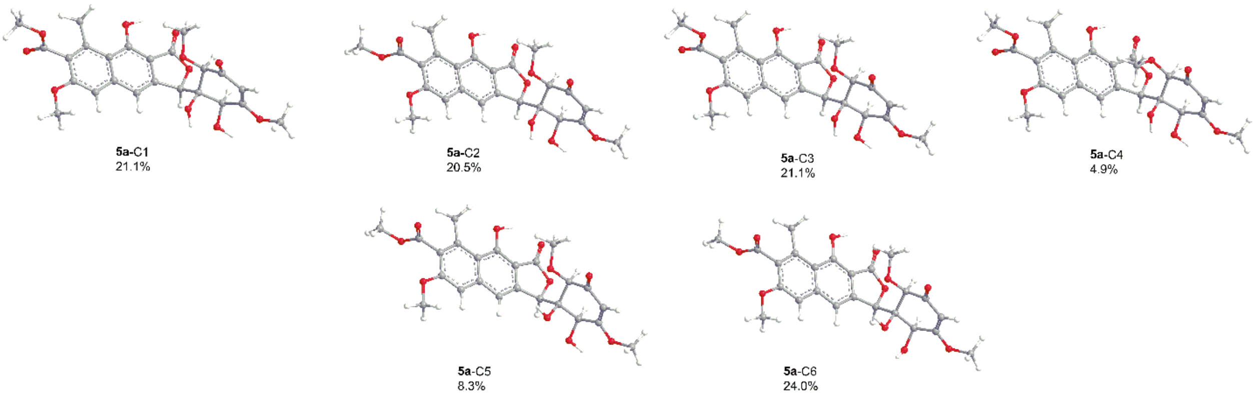

Figure S53. Optimized conformers (≥ 1%) of (4*S*,4a*R*,5*R*,12a*R*)-**5a** at the B3LYP/6-311+G (d,p) level with PCM model in MeOH.
